# Re-examining prophylactic cranial irradiation in small cell lung cancer: a systematic review and meta-analysis

**DOI:** 10.1016/j.eclinm.2023.102396

**Published:** 2024-01-03

**Authors:** Karolina Gaebe, Anders W. Erickson, Alyssa Y. Li, Andrew N. Youssef, Bhagyashree Sharma, Kelvin K.W. Chan, Benjamin H. Lok, Sunit Das

**Affiliations:** a Institute of Medical Science, Faculty of Medicine, University of Toronto, 1 King's College Circle, Toronto, Ontario, Canada; b Department of Laboratory Medicine and Pathobiology, Faculty of Medicine, University of Toronto, 1 King's College Circle, Toronto, Ontario M5S 1A8, Canada; c Division of Medical Oncology, Sunnybrook Health Sciences Centre, Toronto, Ontario, Canada; d Radiation Medicine Program, Princess Margaret Cancer Centre, Toronto, Ontario, Canada; e Division of Neurosurgery, St. Michael's Hospital, University of Toronto, 30 Bond Street, Toronto, Ontario, Canada

**Keywords:** Brain metastases, Small cell lung cancer, SCLC, Prophylactic cranial irradiation, PCI

## Abstract

**Background:**

Patients with small cell lung cancer (SCLC) are at high risk for brain metastases. Prophylactic cranial irradiation (PCI) is recommended in this population to reduce the incidence of brain metastases and prolong survival. We aimed to assesses the efficacy of PCI in this population in the era of routine brain imaging. To our knowledge, this is the first systematic review and meta-analysis to examine the use among patients who were radiographically confirmed not to have brain metastases after completion of first-line therapy.

**Methods:**

In this systematic review and meta-analysis, cohort studies and controlled trials reporting on the use of PCI for patients SCLC were identified in EMBASE, MEDLINE, CENTRAL, and grey literature sources. The literature search was conducted on November 12, 2023. Summary data were extracted. Random-effects meta-analyses pooled hazard ratios (HR) for the primary outcome of overall survival between PCI and no intervention groups. This study is registered with the Open Science Framework, DOI:10.17605/OSF.IO/BC359, and PROSPERO, CRD42021249466.

**Findings:**

Of 4318 identified records, 223 were eligible for inclusion. 109 reported on overall survival in formats amenable to meta-analysis; PCI was associated with longer survival in all patients with SCLC (HR 0.59; 95% CI, 0.55–0.63; p < 0.001; n = 56,770 patients), patients with limited stage disease (HR 0.60; 95% CI, 0.55–0.65; p < 0.001; n = 78 studies; n = 27,137 patients), and patients with extensive stage disease (HR 0.59; 95% CI, 0.51–0.70; p < 0.001; n = 28 studies; n = 26,467 patients). Between-study heterogeneity was significant when pooled amongst all studies (I2 = 73.6%; 95% CI 68.4%–77.9%). Subgroup analysis did not reveal sources of heterogeneity. In a subgroup analysis on studies that used magnetic resonance imaging to exclude presence of brain metastases at restaging among all patients, overall survival did not differ significantly between patients who did or did not receive PCI (HR 0.74; 95% CI, 0.52–1.05; p = 0.08; n = 9 studies; n = 1384 patients).

**Interpretation:**

Our findings suggested that administration of PCI is associated with a survival benefit, but not when considering studies that radiographically confirmed absence of brain metastases, suggesting that the survival benefit conferred by PCI might be therapeutic rather than prophylactic.

**Funding:**

No funding.

**Research in context**

**Evidence before this study**

Patients with small cell lung cancer (SCLC) are at an increased risk for the development of brain metastases. To prevent brain metastases and prolong survival, administration of prophylactic cranial irradiation (PCI) following first-line therapy is recommended, a practice that has been called into question in the current era of improved systemic treatment and imaging. We searched PubMed for systematic reviews published from database inception until November 12, 2023, using search terms related to “small cell lung cancer”, “brain metastases”, and “prophylactic cranial irradiation”, but did not retrieve any meta-analyses that comprehensively assessed survival and incidence of brain metastases with routine screening in relation to PCI in patients with SCLC.

**Added value of this study**

To our knowledge, this is the first systematic review and meta-analysis to survey the literature on PCI and examine survival outcomes in consideration of routine cranial imaging. This study found that PCI was associated with longer survival in patients with SCLC (HR 0.59; 95% CI, 0.55–0.63; p < 0.001; n = 109 studies; n = 56,770 patients), but not in a subgroup analysis of studies that used magnetic resonance imaging to exclude presence of brain metastases following completion of first-line therapy (HR 0.74; 95% CI, 0.52–1.05; p = 0.08; n = 9 studies; n = 1384 patients).

**Implications of all the available evidence**

Our findings suggest that administration of PCI is associated with a survival benefit, but not when limited to studies which radiographically confirmed the absence of brain metastases in all patients regardless of whether they received PCI. This suggests that the survival benefit previously reported in studies may be due to the therapeutic rather than prophylactic effect of cranial irradiation in patients with subclinical brain metastases. Given the concerns regarding neurocognitive effects of PCI and dearth of studies implementing radiographic screening for brain metastases, prospective trials are needed that examine the effect of PCI on survival and intracranial disease outcomes in patients with SCLC.

## Introduction

Nearly 15% of patients with small cell lung cancer (SCLC) present with brain metastases at the time of diagnosis and over half will develop brain metastases within two years.^1^^,^^2^ Despite advances in treatment, survival following development of brain metastases remains guarded, with a median survival estimated to be 3–6 months.^3^, ^4^, ^5^

Many guidelines recommend prophylactic cranial irradiation (PCI) after completion of first-line therapy in all patients with SCLC and good treatment response, as data from randomised controlled trials and meta-analyses have demonstrated prolonged survival and reduced incidence of brain metastases in patients who received prophylactic radiation.^6^, ^7^, ^8^, ^9^, ^10^ However, these favourable outcomes are counterposed by neurotoxicity associated with PCI.^11^^,^^12^ Furthermore, more recent studies incorporating improved systemic disease staging and surveillance brain imaging have failed to replicate the survival benefits seen in patients with SCLC who received PCI in the initial studies.^13^, ^14^, ^15^, ^16^ There is an impetus to re-examine the indications for and the role of PCI in patients with SCLC.

To assess the efficacy and safety of PCI in patients with limited or extensive stage SCLC, we performed this systematic review and meta-analysis to examine survival, disease control, and safety reported in controlled trials and cohort studies.

## Methods

### Search strategy and selection criteria

This systematic review and meta-analysis was conducted in accordance with the PRISMA guidelines and registered in PROSPERO (CRD42021249466).^17^ The complete study protocol is available on Open Science Framework (DOI: 10.17605/OSF.IO/BC359). No individual patient-level data was used.

The literature search was conducted on November 12, 2023, in MEDLINE, EMBASE, the Cochrane Central Register of Controlled Trials (CENTRAL) and grey literature sources using a combination of keywords and MeSH terms related to the terms “small cell lung cancer”, “brain metastases”, and “prophylactic cranial irradiation”. A representative search strategy is available in Supplementary Table S1.

Conference abstracts were eligible. The following grey literature sources were searched: ClinicalTrials.gov, Google Scholar (first 150 results), PROSPERO, and the International Clinical Trials Registry Platform of the World Health Organisation. The home pages of the following societies were searched for relevant conference abstracts: Society for Neuro-oncology (SNO), American Lung Association, American Society of Clinical Oncology (ASCO), and European Society of Medical Oncology (ESMO).

Only articles and abstracts in English were considered due to resource constraints. Case reports, case series, commentaries, and review articles were excluded. Reference lists of identified review articles were scanned to ensure saturation and inclusion of key studies.

Eligible studies included adult patients (age ≥ 18 years) with SCLC in any treatment response and any disease stage who received PCI and reported on comparison with no PCI. Post-hoc, studies that reported on prophylactic cranial irradiation regardless of whether they verified that patients had no evidence of brain metastases in the two comparison cohorts were included to investigate the impact of this covariate on primary and secondary outcomes. No further restrictions were made on patient characteristics, and no limitations were imposed on publication year.

**Fig. 1:**
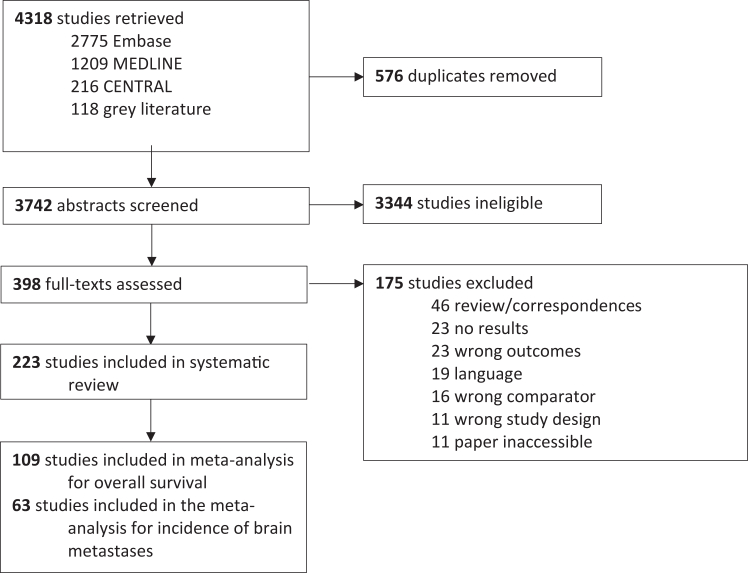
Study selection.

Four reviewers (KG, AL, AY, BS) independently evaluated studies in duplicate by screening abstracts and full texts. Conflicts were resolved through discussion. Cohen's κ statistic was calculated to assess inter-rater reliability at both stages. Reasons for exclusion of full texts are shown in Fig. 1.

### Study outcomes

As in previous meta-analyses, the primary outcome was overall survival. Secondary outcomes were: incidence of intracranial metastatic disease, brain metastasis-free survival, time to brain metastases, progression-free survival, disease-free survival, incidence of brain metastases as first site of recurrence, neurocognitive decline, and adverse events.^7^, ^18^, ^19^, ^20^, ^21^, ^22^ No restrictions were made based on endpoint reporting format, and studies were eligible regardless of whether PCI was the primary intervention under investigation or examined in secondary analyses. All outcomes were prespecified, collected, and reported; outcomes not shown in the manuscript were not reported by included studies.

### Statistical analysis

Four reviewers (KG, AL, AY, BS) extracted study-level data in duplicate using predetermined extraction forms, including study characteristics (author, country, design), patient characteristics (age, sex, smoking status, performance status), treatment characteristics (regimen, response, imaging, treatment following brain metastasis diagnosis), and primary and secondary outcomes. Disagreements were resolved through discussion. Only variables specific to management of brain metastases in patients with SCLC were extracted. Investigators were not contacted due to resource constraints. Quality assessment of observational studies and RCTs was performed using the Newcastle–Ottawa Scale and Cochrane Risk of Bias 2 (RoB 2), respectively.^23^^,^^24^

Where summary survival estimates were not reported, Kaplan–Meier curves were digitised and summary estimates were calculated using the method by Guyot et al.^25^^,^^26^ The extracted pseudo-individual patient data was used to generate HRs for PCI versus no intervention. For any given analysis, all patients were included if their corresponding outcome was reported in a format amenable to pooling.

Meta-analyses using random-effects models were performed to pool hazard ratio (HR) estimates and risk ratio (RR) estimates for time-to-event analyses and incidence rates, respectively. The heterogeneity variance, ***τ***^2^, was calculated using the restricted maximum likelihood estimator, and Knapp-Hartung adjustments were used to calculate the confidence interval (CI) around the pooled effect.^27^, ^28^, ^29^ Analyses were performed for the overall patient population and stratified according to disease stage whenever outcomes were reported on by more than three studies.

Subgroup analyses and meta-regression were performed according to pre-specified covariates. Due to underreporting and inconsistent reporting, pre-specified subgroup analysis by type of systemic therapy regimen, SCLC disease subtypes, treatment after prophylactic cranial irradiation and/or development of brain metastases, and observation frequency were not performed. The protocol also pre-specified analysis of studies comparing prophylactic cranial irradiation with or without hippocampal avoidance. However, only three studies were identified that compared these two interventions and were not meta-analysed due to limited sample size and heterogeneity of study design.^30^, ^31^, ^32^

Statistical heterogeneity was assessed using *I*^2^ and *Q* statistics, with *I*^2^ values above and below 50% signifying high and low between-study heterogeneity, respectively.^33^ Influence analysis to identify potential sources of heterogeneity was performed based on outlier identification and leave-one-out analysis.^34^ Egger's test and funnel plot inspection were performed to assess for publication bias.^35^

All statistical analyses were performed using the R programming language (version 4.0.3) using the package {meta}.^36^^,^^37^ An ***α*** of 0.05 was considered statistically significant. All tests were two-sided.

### Ethics

As this article is a systematic review, it was exempt from ethical approval and need for participant informed consent.

### Role of the funding source

There was no funding for this study. All authors had full access to all data, critically revised the report for important intellectual content, gave final approval of the version to be published, and agree to be accountable for all aspects of the work.

## Results

### Study population

A total of 223 studies reporting on 73,777 patients with SCLC were identified that compared outcomes between patients who did and did not receive PCI (Fig. 1). The majority were retrospective cohort studies (n = 199); other study designs included randomised controlled trials (n = 13), prospective cohort studies (n = 10), and non-randomised controlled trials (n = 1); 163 were published as full-text manuscripts, and 60studies were reported as abstracts. Median follow-up duration ranged from 5.6–165 months.

### Overall survival

**Fig. 2:**
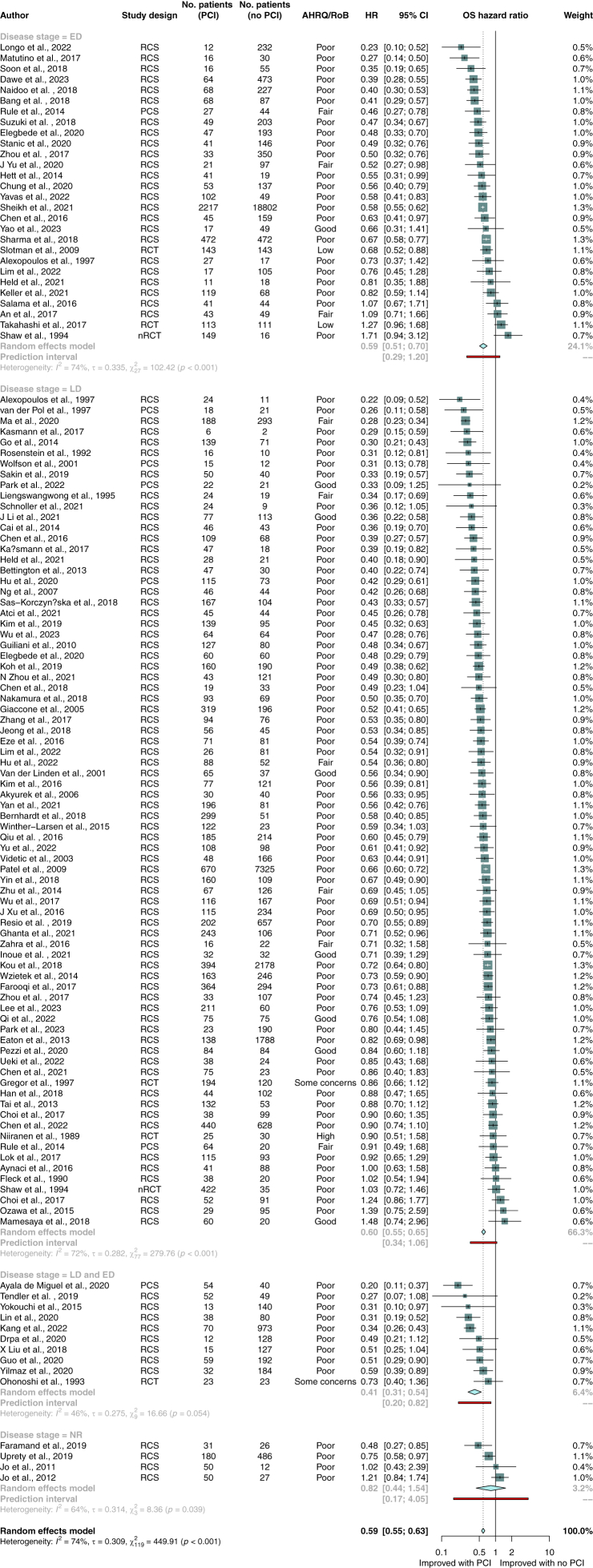
Random-effects meta-analysis of PCI versus no PCI for primary outcome of overall survival. Studies are stratified by disease stage. Boxes represent individual study effect sizes. The vertical solid line represents the point of equivalence between PCI and no PCI. The vertical dotted line represents the points of summary for the random effects model. The diamonds represent 95% CI for the summary hazard ratios. The red box represents the summary prediction interval. 95% CI = 95% confidence interval; AHRQ = Agency for Health Research and Quality; ED = extensive stage disease; HR = hazard ratio; LD = limited stage disease; NR = not reported; nRCT = non-randomised controlled trial; OS = overall survival; PCI = prophylactic cranial irradiation; RCS = retrospective cohort study; RCT = randomised controlled trial; RoB = risk of bias.

Out of 201 studies that reported overall survival, more than half (n = 109 studies, n = 56,770 patients) reported adjusted or unadjusted hazard ratios and were eligible for meta-analysis (Fig. 2). PCI was associated with longer survival in all patients with SCLC (HR 0.59; 95% CI, 0.55–0.63; p < 0.001), patients with limited stage disease (HR 0.60; 95% CI, 0.55–0.65; p < 0.001; n = 78 studies; n = 27,137 patients), and patients with extensive stage disease (HR 0.59; 95% CI, 0.51–0.70; p < 0.001; n = 28 studies; n = 26,467 patients). On post-hoc sensitivity analyses of unadjusted and adjusted hazard ratios, PCI was associated with longer survival (unadjusted HR 0.60; 95% CI, 0.55–0.65; p < 0.001; n = 91 studies; n = 52,928 patients; adjusted HR 0.59; 95% CI, 0.55–0.64; p < 0.001; n = 72 studies; n = 32,879 patients; Supplementary Figs. S1 and S2).

**Fig. 3:**
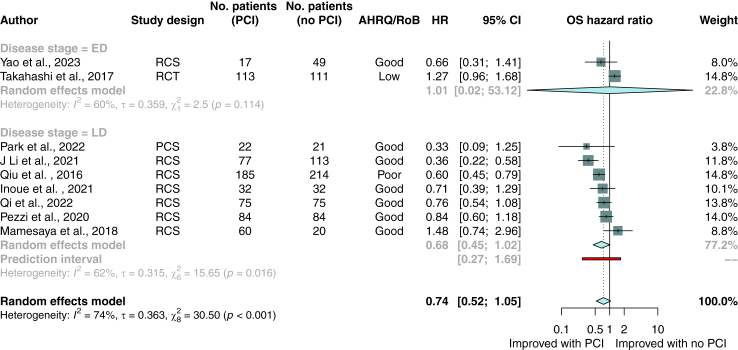
Random-effects meta-analysis of PCI versus no PCI for primary outcome of overall survival among patients confirmed not to have brain metastases via MRI after completion of first-line therapy. Studies are stratified by disease stage. Boxes represent individual study effect sizes. The vertical solid line represents the point of equivalence between PCI and no PCI. The vertical dotted line represents the points of summary for the random effects model. The diamonds represent 95% CI for the summary hazard ratios. The red box represents the summary prediction interval. 95% CI = 95% confidence interval; AHRQ = Agency for Health Research and Quality; ED = extensive stage disease; HR = hazard ratio; LD = limited stage disease; MRI = magnetic resonance imaging; NR = not reported; OS = overall survival; PCI = prophylactic cranial irradiation; RCS = retrospective cohort study; RCT = randomised controlled trial; RoB = risk of bias.

Post-hoc subgroup analysis was performed on studies that used brain magnetic resonance imaging to exclude patients with radiographic evidence of brain metastases at restaging. One of these studies was a randomised controlled trial; the remaining studies were cohort studies reporting unadjusted hazard ratios. In this analysis, overall survival did not differ significantly between patients who did or did not receive PCI (HR 0.74; 95% CI, 0.52–1.05; p = 0.08; n = 9 studies; n = 1384 patients; Fig. 3).

Pre-specified subgroup analyses were performed on all included studies, as well as separated by studies consisting exclusively of patients with limited and extensive stage SCLC, evaluating study design, cohort treatment response to first-line therapy, use of baseline and restaging imaging, use of platinum-based chemotherapy, and risk of bias assessments (Table 1). Subgroup analysis did not reveal any sources of heterogeneity. Meta-regression was performed on publication year, total study sample size, and median age of participants (Appendix 1, p. 10). Neither predictor led to a significant reduction in study heterogeneity when considering all included studies (p = 0.09, p = 0.80, and p = 0.16, respectively), or when investigated separately for limited (p = 0.55, p = 0.14, and p = 0.08, respectively) or extensive stage patients (p = 0.06, p = 0.86, and p = 0.51, respectively). Further results for sensitivity and subgroup analysis, and narrative synthesis of studies not amenable to meta-analysis can be found in the appendix (Appendix 1, pp. 13–23).

**Table 1:** Pooled hazard ratios for subgroup analysis of overall survival.

Study characteristic	All included studies	Limited stage disease patients only	Extensive stage disease patients only
Study design			
RCT	0.88 (95% CI 0.65–1.18), n = 5 studies	0.87 (95% CI 0.65–1.12), n = 2 studies	0.93 (95% CI 0.02–48.93), n = 2 studies
RCS	0.58 (95% CI 0.54–0.62), n = 106 studies	0.56 (95% CI 0.5–0.64), n = 70 studies	0.55 (95% CI 0.48–0.64), n = 24 studies
PCS	0.39 (95% CI 0.28–0.54), n = 7 studies	0.44 (95% CI 0.24–0.81), n = 5 studies	0.46 (95% CI 0.22–0.94), n = 1 study
nRCT	1.25 (95% CI 0.75–2.09), n = 2 studies	1.03 (95% CI 0.54–1.96), n = 2 studies	1.71 (95% CI 0.79–3.72), n = 1 study
Treatment response to first-line therapy			
CR	0.70 (95% CI 0.48–0.98), n = 13 studies	0.57 (95% CI 0.45–0.71), n = 10 studies	1.14 (95% CI 0.00–263.19), n = 2 studies
CR/PR	0.65 (95% CI 0.56–0.75), n = 26 studies	0.55 (95% CI 0.48–0.61), n = 19 studies	0.71 (95% CI 0.47–1.08), n = 6 studies
CR/PR/SD	0.55 (95% CI 0.42–0.72), n = 12 studies	0.59 (95% CI 0.42–0.82), n = 7 studies	0.49 (95% CI 0.25–0.97), n = 5 studies
CR/PR/SD/PD	0.51 (95% CI 0.34–0.78), n = 7 studies	0.50 (95% CI 0.29–0.87), n = 5 studies	0.48 (95% CI 0.46–4.172), n = 2 study
NR	0.57 (95% CI 0.52–0.62), n = 62 studies	0.56 (95% CI 0.53–0.60), n = 37 studies	0.56 (95% CI 0.46–0.68), n = 13 studies
Use of brain baseline brain CT/MRI			
Yes	0.60 (95% CI 0.53–0.68), n = 49 studies	0.56 (95% CI 0.52–0.60), n = 34 studies	0.60 (95% CI 0.42–0.86), n = 13 studies
No, NR, only in a subset of patients	0.58 (95% CI 0.51–0.64), n = 171 studies	0.59 (95% CI 0.52–0.66), n = 44 studies	0.61 (95% CI 0.50–0.73), n = 14 studies
MRI confirmation of no brain metastases at restaging			
Yes	0.75 (95% CI, 0.50–1.12), n = 9 studies	0.68 (95% CI 0.45–1.02), n = 7 studies	1.27 (95%CI 0.70–2.29), n = 1 study
No	0.62 (95% CI 0.54–0.71), n = 19 studies	0.59 (95% CI 0.50–0.71), n = 14 studies	0.69 (95% CI 0.52–0.91), n = 4 studies
NR	0.57 (95% CI 0.52–0.62), n = 84 studies	0.58 (95% CI 0.52–0.65), n = 51 studies	0.57 (95% CI 0.48–0.69), n = 20 studies
Use of platinum-based therapy			
Yes	0.56 (95% CI 0.51–0.62); n = 74	0.58 (95% CI 0.50–0.63), n = 51 studies	0.65 (95% CI 0.51–0.83), n = 14 studies
No	0.59 (95% CI 0.27–1.30); n = 4 studies	0.55 (95% CI 0.13–2.32), n = 3 studies	NA
Not administered to all patients	0.58 (95% CI 0.45–0.74); n = 15 studies	0.57 (95% CI 0.43–0.76), n = 10 studies	0.61 (95% CI 0.29–1.29), n = 5 studies
NR	0.65 (95% CI 0.56–0.75); n = 27 studies	0.72 (95% CI 0.64–0.85), n = 14 studies	0.56 (95% CI 0.45–0.69), n = 6 studies
AHRQ^a^			
Good	0.67 (95% CI 0.47–0.96), n = 7 studies	0.68 (95% CI 0.45–1.02), n = 7 studies	0.66 (95% CI 0.24–1.75), n = 3 studies
Fair	0.55 (95% CI 0.38–0.78), n = 9 studies	0.62 (95% CI 0.49–0.78), n = 6 studies	0.67 (95% CI 0.20–2.24), n = 3 studies
Poor	0.57 (95% CI 0.53–0.62), n = 99 studies	0.56 (95% CI 0.54–0.59), n = 63 studies	0.55 (95% CI 0.47–0.66), n = 22 studies
RoB^b^			
Low	0.93 (95% CI 0.02–49.90), n = 2 studies	NA	0.93 (95% CI 0.02–48.99), n = 2 studies
Some concerns	0.82 (95% CI 0.31–2.13), n = 2 studies	0.86 (95% CI 0.57–1.58), n = 1 study	NA
High	0.90 (95% CI 0.40–2.05), n = 1 study	0.90 (95% CI 0.41–1.98), n = 1 study	NA

AHRQ = Agency for Health Research and Quality; CR = complete response; CT = computed tomography; MRI = magnetic resonance imaging; NA = not applicable; NR = not reported; nRCT = non-randomised controlled trial; PCS = prospective cohort study; PR = partial response; SD = stable disease; RCS = retrospective cohort study; RCT = randomised controlled trial; RoB = risk of bias.

a AHRQ only reported for non-randomised trials.

b RoB only reported for randomised controlled trials.

Study quality assessment of randomised controlled trials found two studies each at low risk of bias or with some concerns, and one study at high risk of bias. Study quality based on the Agency of Health Research and Quality rating among retrospective cohort studies was good, fair, and poor in eight, nine, and 98 studies respectively (Supplementary Figs. S6 and S7).

### Incidence of brain metastases

**Fig. 4:**
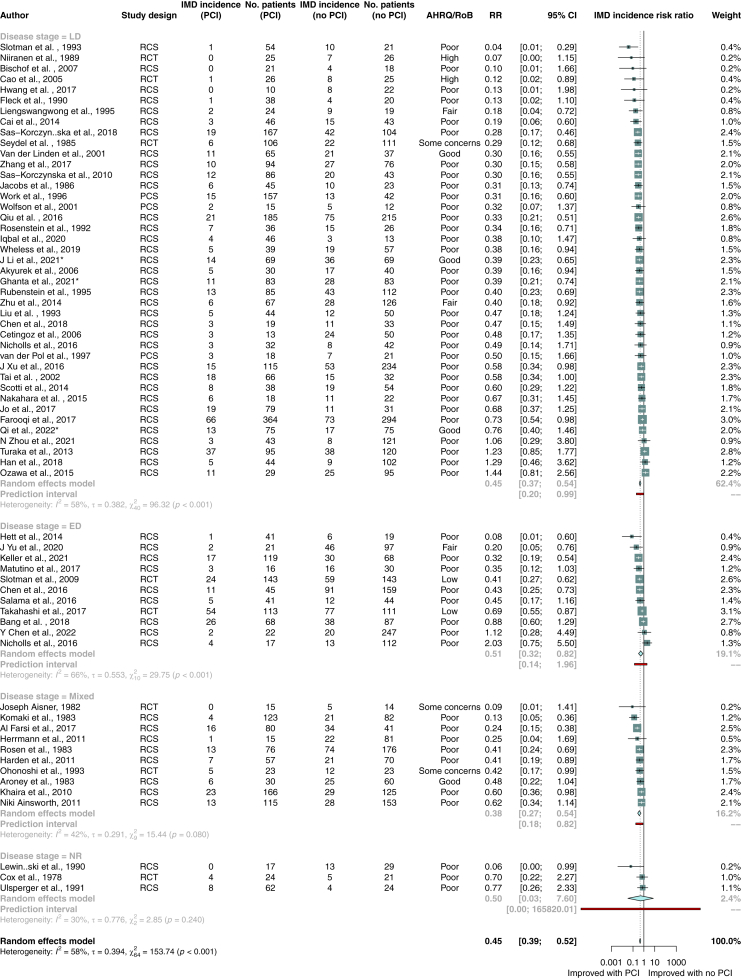
Random-effects meta-analysis of PCI versus no PCI for secondary outcome of brain metastases incidence. Studies are stratified by disease stage. Boxes represent individual study effect sizes. The vertical solid line represents the point of equivalence between PCI and no PCI. The vertical dotted line represents the points of summary for the random effects model. The diamonds represent 95% CI for the summary hazard ratios. The red box represents the summary prediction interval. 95% CI = 95% confidence interval; AHRQ = Agency for Health Research and Quality; ED = extensive stage disease; HR = hazard ratio; LD = limited stage disease; NR = not reported; nRCT = non-randomised controlled trial; OS = overall survival; PCI = prophylactic cranial irradiation; RCS = retrospective cohort study; RCT = randomised controlled trial; RoB = risk of bias.

The incidence of brain metastases was lower in all patients with SCLC who received PCI (RR 0.45; 95% CI, 0.39–0.52; p < 0.001; n = 63 studies; n = 8906 patients), and in patients with limited stage (RR 0.45; 95% CI, 0.37–0.54; p < 0.0001; n = 41 studies; n = 5470 patients) or extensive stage disease (RR 0.51; 95% CI, 0.32–0.82; p = 0.01; n = 11 studies; n = 1763 patients; Fig. 4). Four studies used magnetic resonance imaging to confirm absence of brain metastases at restaging and found a lower incidence of brain metastases in patients who received PCI (RR 0.51; 95% CI, 0.28–0.99; p = 0.047; n = 912 patients; p = 0.047). Further analysis results can be found in the appendix (Appendix 1, pp. 24–39).

### Other secondary outcomes

Results from the analyses of other secondary outcomes: brain metastasis-free survival, progression-free survival, disease-free survival, and incidence of brain metastases as first site of recurrence are described in the appendix (Appendix 1, pp. 30–54). Adverse event outcomes were reported in various formats, which precluded meta-analysis, and are listed in the appendix (Appendix 1, p. 55).

## Discussion

In this meta-analysis, PCI was associated with improved overall survival and reduced cumulative incidence of brain metastases in patients with SCLC, irrespective of disease stage. However, in a subgroup analysis limited to studies in which all patients underwent magnetic resonance imaging after completion of initial chemotherapy or chemoradiotherapy and excluded patients found to have brain metastases, PCI was associated with decreased incidence of brain metastases but had no effect on survival. This systematic review and meta-analysis captures the entire literature on PCI, making it feasible for the first time to address concerns around the efficacy of routine brain imaging as part of restaging investigations.

Our findings are discordant with those of prior meta-analyses that have found a protective effect of the use of PCI on survival.^7^^,^^18^^,^^20^, ^21^, ^22^^,^^38^ Several meta-analyses have also been published in an attempt to assess the results by Takahashi et al., however these only examined the impact of restaging modality^39^ or did not control for absence of brain metastases among patients who did not receive PCI.^21^^,^^40^^,^^41^ None of these studies, therefore, excluded the presence of brain metastases among the control cohort of patients who did not receive PCI. In a previous study, 13% of patients presented with asymptomatic brain metastases at the time of diagnosis, which remained asymptomatic in a substantial portion of patients after completion of chemotherapy.^42^ In another report, asymptomatic brain metastases were identified in 33% of patients with limited stage SCLC initially thought to have good response to chemoradiotherapy.^43^ Consequently, previous evidence likely included patients in the treatment or control groups who harboured asymptomatic brain metastases and received a therapeutic benefit from PCI in the treatment groups or showed shortened survival confounded by the presence of brain metastases in the control groups. The current study is the first to control for the absence of brain metastases among all patients irrespective of PCI receipt—a pivotal differentiating feature between study by Takahashi et al. and previous trials—and suggest a need to re-examine the conclusions derived from remote studies while we await results from prospective trials.

Nearly 25% of patients with SCLC present with synchronous brain metastases and a large proportion of patients may further develop brain metastases between diagnosis and completion of first-line therapy with the majority of patients remaining asymptomatic from their intracranial progression.^42^, ^43^, ^44^ Conversely, more than half of patients with SCLC will not have intracranial disease following completion of first-line therapy. In this context, one reasonable alternative to treatment with PCI is active surveillance imaging to limit unnecessary exposure to cranial radiation and the attendant risk of neurotoxicity. Previous cost-benefit analyses have demonstrated no cost benefit to the use of PCI compared with magnetic resonance imaging surveillance in extensive disease SCLC.^45^ To this effect, the American Society for Radiation Oncology (ASTRO) guidelines and the Canadian Consensus recommendations suggest magnetic resonance surveillance as an alternative in patients with extensive disease SCLC.^8^^,^^46^ Although prospective data in limited disease SCLC are inconclusive, the benefit of this approach could be longstanding, as patients with SCLC found to have brain metastases on surveillance imaging may be treated with stereotactic radiosurgery instead of whole brain irradiation, thus further improving quality of life in these patients without compromising survival outcomes.^47^

At the same time, access to magnetic resonance imaging technology is not uniformly available across the world.^48^ In clinical contexts where consistent follow-up with brain imaging is not a viable option, the decision to omit PCI needs to be approached with caution. This is particularly important given the potential risk of developing functionally impairing brain metastases, regardless of whether the primary benefit of PCI is prophylactic or therapeutic in nature. To our knowledge, there is no real-world data on the implementation on surveillance guideline recommendations for brain imaging. This lack of data limits our analysis of the feasibility of offering routine brain imaging as an alternative to PCI outside the context of clinical trials. Based on current treatment guidelines, the omission of PCI should be contemplated only in scenarios where regular, systematic brain imaging follow-up can be reliably implemented. This approach is crucial to ensure optimal patient outcomes and to minimise the risk of late-stage diagnosis of brain metastases, which can significantly impair patient quality of life and limit treatment options.

A major shortcoming of most prior studies on PCI in patients with SCLC has been the dearth of radiographic screening for brain metastases after completion of chemotherapy, without which it is impossible to discern if radiation is therapeutic or preventive^7^^,^^49^: of 109 reports that investigated associations between PCI and overall survival, as reflected in our subgroup analysis only nine ensured that all patients had no brain metastases after initial therapy, which is historically when PCI is delivered. Additionally, of 95 studies that reported survival outcomes and were not amenable to meta-analysis, only five excluded patients with radiographic evidence for brain metastases. The present analysis is the first to include all available published evidence on the use of PCI in SCLC, raising the concern that survival estimates following PCI in patients with SCLC might be inflated by inclusion of patients with asymptomatic intracranial disease.

In the present study, the incidence of brain metastases was lower in patients who received prophylactic cranial radiation irrespective of subgroup analysis. The reduction in brain metastases in the light of non-equivocal survival is not surprising, given similar outcomes following PCI in patients with non-small cell lung cancer.^50^ A possible explanation is that intracranial progression may not be a primary driver of mortality. In a recent meta-analysis, our group has shown that patients with stable extracranial disease have superior survival outcomes compared with those who have progressive extracranial disease.^51^ This suggests that the reduced overall survival in patients with brain metastases may be a consequence of extracranial rather than intracranial disease progression and would therefore be independent of administration of PCI. Assessing the relationship between overall survival and development of intracranial disease is further confounded by the fact that PCI is preferentially administered to patients with response to first-line treatment and better performance status.^9^, ^10^, ^52^

This systematic review and meta-analysis has several limitations. First, the majority of studies included were retrospective observational studies with poor study quality and limited reporting on patient eligibility for PCI, leading to confounding and selection bias. However, sensitive search criteria including retrospective studies allowed for description and analysis of real-world practices and comprehensive subgroup analysis. Second, only one randomised controlled trial was available reporting on outcomes on patients who were restaged using magnetic resonance imaging, limiting our ability to rely on this highest level of evidence. The remaining studies included in this subgroup analysis were retrospective cohort studies with an overall “good” Newcastle Ottawa Score, the highest one possible. We eagerly await the results of ongoing trials that investigate the use of PCI versus routine surveillance in patients who were confirmed to have no brain metastases using magnetic resonance imaging (MAVERICK, NCT04155034; PRIMALung, NCT04790253). Third, there was significant in-between study heterogeneity in terms of study design, inclusion criteria, follow-up, and treatment schedules. Despite extensive subgroup analysis, we were unable to identify a statistical source for this heterogeneity, an expected finding given the large number of included studies, which makes it unlikely to identify a single causal variable. Fourth, neurotoxicity and quality of life, key outcomes that inform patient-physician discussion of administration of PCI, were underreported. Given that PCI in patients with SCLC with imaging evidence confirming absence of brain metastases following first-line therapy was associated with a reduction in brain metastases but no survival advantage, the issue of neurotoxic sequelae, which has prompted the field otherwise toward stereotactic radiosurgery rather than whole brain radiation therapy, is in critical need of reassessment in prospective studies. Last, PCI was assessed only in subgroup or multivariable analysis in many studies, leading to disparate confounders included in multivariable models. In consideration of this concern, we performed a sensitivity analysis of only adjusted and unadjusted hazard ratios and found no change in overall hazard ratio.

In summary, in patients with SCLC, PCI was associated with improved overall survival and reduced cumulative incidence of brain metastases in patients with SCLC, irrespective of disease stage. However, following exclusion of patients with radiographic evidence of intracranial disease on imaging performed at the completion of first-line therapy, PCI was associated with decreased incidence of brain metastases but had no effect on survival. Our findings suggest that the survival benefit conferred by PCI might be therapeutic rather than prophylactic in nature and should prompt reconsideration in its default role in clinical care in the modern context of magnetic resonance imaging.

## Contributors

KG, AE, and SD conceived and designed the study. KG, AL, AY, and BS independently assessed studies for possible inclusion and collected the data. KG analysed the data with results checked and verified by AE. All authors had full access to all data, critically revised the report for important intellectual content, gave final approval of the version to be published, and agree to be accountable for all aspects of the work. KG had final responsibility to submit for publication.

## Data sharing statement

Extracted data are available on request to the corresponding author.

## Declaration of interests

BHL declares institution grants from AstraZeneca and Pfizer. BHL declares honoraria and support for travel/attending meetings from AstraZeneca.

SD has received grant funding from the Canadian Institute for Health Research, Gratitude 10, the Canadian Cancer Society, and the Calum Macbeth fund and has received funding as Keenan Chair in Surgery. SD received royalties from Oxford University Press. SD reports support for travel and accommodation from the Congress of Neurological Surgeons and the American Association of Neurological Surgeons. SD participated on a data safety monitoring board or advisory board for the Subcortical Surgery Group and XPan Medical. SD as received a stipend as the provincial lead Provincial Lead for CNS Cancers, Ontario Health, and Cancer Care Ontario. SD reports a research grant from Alkermes.

## Appendix ASupplementary data

Appendix 2

Study Protocol

## References

[1] Hochstenbag M.M., Twijnstra A., Wilmink J.T., Wouters E.F., ten Velde G.P. (2000). Asymptomatic brain metastases (BM) in small cell lung cancer (SCLC): MR-imaging is useful at initial diagnosis. J Neuro Oncol.

[2] Patchell R.A. (1991). Brain metastases. Neurol Clin.

[3] Howlader N., Forjaz G., Mooradian M.J. (2020). The effect of advances in lung-cancer treatment on population mortality. N Engl J Med.

[4] Bernhardt D., Bozorgmehr F., Adeberg S. (2016). Outcome in patients with small cell lung cancer re-irradiated for brain metastases after prior prophylactic cranial irradiation. Lung Cancer.

[5] Postmus P.E., Haaxma-Reiche H., Gregor A. (1998). Brain-only metastases of small cell lung cancer; efficacy of whole brain radiotherapy. An EORTC phase II study. Radiother Oncol.

[6] Slotman B., Faivre-Finn C., Kramer G. (2007). Prophylactic cranial irradiation in extensive small-cell lung cancer. N Engl J Med.

[7] Aupérin A., Arriagada R., Pignon J.P. (1999). Prophylactic cranial irradiation for patients with small-cell lung cancer in complete remission. Prophylactic Cranial Irradiation Overview Collaborative Group. N Engl J Med.

[8] Simone C.B., Bogart J.A., Cabrera A.R. (2020). Radiation therapy for small cell lung cancer: an ASTRO clinical practice guideline. Pract Radiat Oncol.

[9] Network NCC (2022). Small cell lung cancer.

[10] Jett J.R., Schild S.E., Kesler K.A., Kalemkerian G.P. (2013). Treatment of small cell lung cancer: diagnosis and management of lung cancer, 3rd ed: American College of Chest Physicians evidence-based clinical practice guidelines. Chest.

[11] Lee J.J., Bekele B.N., Zhou X., Cantor S.B., Komaki R., Lee J.S. (2006). Decision analysis for prophylactic cranial irradiation for patients with small-cell lung cancer. J Clin Oncol.

[12] Wolfson A.H., Bae K., Komaki R. (2011). Primary analysis of a phase II randomized trial Radiation Therapy Oncology Group (RTOG) 0212: impact of different total doses and schedules of prophylactic cranial irradiation on chronic neurotoxicity and quality of life for patients with limited-disease small-cell lung cancer. Int J Radiat Oncol Biol Phys.

[13] Takahashi T., Yamanaka T., Seto T. (2017). Prophylactic cranial irradiation versus observation in patients with extensive-disease small-cell lung cancer: a multicentre, randomised, open-label, phase 3 trial. Lancet Oncol.

[14] Pezzi T.A., Fang P., Gjyshi O. (2020). Rates of overall survival and intracranial control in the magnetic resonance imaging era for patients with limited-stage small cell lung cancer with and without prophylactic cranial irradiation. JAMA Netw Open.

[15] Ghanta S., Keller A., Rodríguez-López J.L., Patel A., Beriwal S. (2021). Utility of prophylactic cranial irradiation for limited stage small cell lung cancer in the modern era with magnetic resonance imaging surveillance. Clin Oncol.

[16] Farris M.K., Wheless W.H., Hughes R.T. (2019). Limited-stage small cell lung cancer: is prophylactic cranial irradiation necessary?. Pract Radiat Oncol.

[17] Moher D., Liberati A., Tetzlaff J., Altman D.G. (2009). Preferred reporting items for systematic reviews and meta-analyses: the PRISMA statement. BMJ.

[18] Maeng C.H., Song J.U., Shim S.R., Lee J. (2018). The role of prophylactic cranial irradiation in patients with extensive stage small cell lung cancer: a systematic review and meta-analysis. J Thorac Oncol.

[19] Meert A.P., Paesmans M., Berghmans T. (2001). Prophylactic cranial irradiation in small cell lung cancer: a systematic review of the literature with meta-analysis. BMC Cancer.

[20] Tomassen M.L., Pomp J., van der Stap J. (2022). The overall survival impact of prophylactic cranial irradiation in limited-stage small-cell lung cancer: a systematic review and meta-analysis. Clin Transl Radiat Oncol.

[21] Yin X., Yan D., Qiu M., Huang L., Yan S.X. (2019). Prophylactic cranial irradiation in small cell lung cancer: a systematic review and meta-analysis. BMC Cancer.

[22] Zhang W., Jiang W., Luan L., Wang L., Zheng X., Wang G. (2014). Prophylactic cranial irradiation for patients with small-cell lung cancer: a systematic review of the literature with meta-analysis. BMC Cancer.

[23] Wells G.A., Shea B., O'Connell D. (2021). http://www.ohri.ca/programs/clinical_epidemiology/oxford.asp.

[24] Sterne J.A.C., Savović J., Page M.J. (2019). RoB 2: a revised tool for assessing risk of bias in randomised trials. BMJ.

[25] Guyot P., Ades A.E., Ouwens M.J., Welton N.J. (2012). Enhanced secondary analysis of survival data: reconstructing the data from published Kaplan-Meier survival curves. BMC Med Res Methodol.

[26] Saluja R., Cheng S., Delos Santos K.A., Chan K.K.W. (2019). Estimating hazard ratios from published Kaplan-Meier survival curves: a methods validation study. Res Synth Methods.

[27] Knapp G., Hartung J. (2003). Improved tests for a random effects meta-regression with a single covariate. Stat Med.

[28] Langan D., Higgins J.P.T., Jackson D. (2019). A comparison of heterogeneity variance estimators in simulated random-effects meta-analyses. Res Synth Methods.

[29] Viechtbauer W. (2005). Bias and efficiency of meta-analytic variance estimators in the random-effects model. J Educ Behav Stat.

[30] Belderbos J.S.A., De Ruysscher D.K.M., De Jaeger K. (2021). Phase 3 randomized trial of prophylactic cranial irradiation with or without Hippocampus avoidance in SCLC (NCT01780675). J Thorac Oncol.

[31] De Dios N.R., Murcia M., Counago F. (2019). Phase III trial of prophylactic cranial irradiation with or without hippocampal avoidance for SMALL-CELL LUNG cancer. Int J Radiat Oncol Biol Phys.

[32] Harden S.V., Ainsworth N., Saunders D., Magee L. (2011). Small cell lung cancer overall survival, incidence of brain metastases and use of prophylactic cranial irradiation for patients treated in Cambridge during 2005-2009. Lung Cancer.

[33] Higgins J.P., Thompson S.G., Deeks J.J., Altman D.G. (2003). Measuring inconsistency in meta-analyses. BMJ.

[34] Viechtbauer W., Cheung M.W.L. (2010). Outlier and influence diagnostics for meta-analysis. Res Synth Methods.

[35] Egger M., Davey Smith G., Schneider M., Minder C. (1997). Bias in meta-analysis detected by a simple, graphical test. BMJ.

[36] R Development Core Team (2020).

[37] Schwarzer G. (2007). meta: an R package for meta-analysis. R News.

[38] Wen P., Wang T.F., Li M., Yu Y., Zhou Y.L., Wu C.L. (2020). Meta-analysis of prophylactic cranial irradiation or not in treatment of extensive-stage small-cell lung cancer: the dilemma remains. Cancer Radiother.

[39] Maroufi S.F., Fallahi M.S., Kankam S.B., Sheehan J.P. (2023). Prophylactic cranial irradiation effect on survival in patients with small cell lung cancer: a comprehensive systematic review and meta-analysis. Neurosurg Focus.

[40] Liu J., Shen B., Yang Y. (2023). Survival benefit of prophylactic cranial irradiation in limited-stage small-cell lung cancer in modern magnetic resonance imaging staging: a systematic review and meta-analysis. Acta Oncol.

[41] Ge W., Xu H., Yan Y., Cao D. (2018). The effects of prophylactic cranial irradiation versus control on survival of patients with extensive-stage small-cell lung cancer: a meta-analysis of 14 trials. Radiat Oncol.

[42] Seute T., Leffers P., Wilmink J.T., ten Velde G.P., Twijnstra A. (2006). Response of asymptomatic brain metastases from small-cell lung cancer to systemic first-line chemotherapy. J Clin Oncol.

[43] Manapov F., Klautke G., Fietkau R. (2008). Prevalence of brain metastases immediately before prophylactic cranial irradiation in limited disease small cell lung cancer patients with complete remission to chemoradiotherapy: a single institution experience. J Thorac Oncol.

[44] Seute T., Leffers P., ten Velde G.P., Twijnstra A. (2008). Detection of brain metastases from small cell lung cancer: consequences of changing imaging techniques (CT versus MRI). Cancer.

[45] Kim H., Keller A., Beriwal S., Smith K.J., Vargo J.A. (2021). Cost-effectiveness of prophylactic cranial irradiation versus MRI surveillance for extensive-stage small cell lung cancer. Int J Radiat Oncol Biol Phys.

[46] Melosky B.L., Leighl N.B., Dawe D. (2023). Canadian Consensus recommendations on the management of extensive-stage small-cell lung cancer. Curr Oncol.

[47] Gaebe K., Li A.Y., Park A. (2022). Stereotactic radiosurgery versus whole brain radiotherapy in patients with intracranial metastatic disease and small-cell lung cancer: a systematic review and meta-analysis. Lancet Oncol.

[48] Ogbole G.I., Adeyomoye A.O., Badu-Peprah A., Mensah Y., Nzeh D.A. (2018). Survey of magnetic resonance imaging availability in West Africa. Pan African Medical Journal.

[49] Schild S.E., Foster N.R., Meyers J.P. (2012). Prophylactic cranial irradiation in small-cell lung cancer: findings from a north central cancer treatment group pooled analysis. Ann Oncol.

[50] Li M., Wang T., Wen P., Wang X., Wu C. (2021). Treatment and toxic effects of prophylactic cranial irradiation in stage II-III non-small cell lung cancer: a meta-analysis. Asia Pac J Clin Oncol.

[51] Li A.Y., Gaebe K., Zulfiqar A. (2023). Association of brain metastases with survival in patients with limited or stable extracranial disease: a systematic review and meta-analysis. JAMA Netw Open.

[52] Tomassen M.L., Aarts M.J., Peters M. (2021). Prophylactic cranial irradiation in patients with small cell lung cancer in The Netherlands: a population-based study. Clin Transl Radiat Oncol.

