# Supplementary material for: Re-examining prophylactic cranial irradiation in small cell lung cancer: a systematic review and meta-analysis
Source: eClinicalMedicine. 2024 Jan 3;67:102396. doi: 10.1016/j.eclinm.2023.102396 (PMC10796984; doi:10.1016/j.eclinm.2023.102396)
Supplement: Appendix 1 [file mmc1.pdf]

Table of Contents

List of included studies *3*
Primary outcome: overall survival *8*
Subgroup and heterogeneity analysis 8
Narrative synthesis 18
Secondary outcome: incidence of intracranial metastatic disease *20*
Subgroup and heterogeneity analysis 20
Narrative synthesis 25
Secondary outcome: intracranial progression-free survival *26*
Subgroup and heterogeneity analysis 28
Narrative synthesis 32
Secondary outcome: progression-free survival *33*
Subgroup and heterogeneity analysis 33
Narrative synthesis 38
Secondary outcome: disease-free survival *39*
Subgroup and heterogeneity analysis 39
Narrative synthesis 44
Secondary outcome: incidence of brain metastases as first site of recurrence *45*
Subgroup analysis and between-study heterogeneity 45
Narrative synthesis 50
Secondary outcomes: neurocognitive decline and adverse events *51*
References *52*

Table S1. Full electronic search strategy in EMBASE (Nov 12, 2023).

ID	Search term	Results
1	("small cell lung cancer" or "small-cell lung cancer" or "small-cell lung carcinoma" or "small cell lung carcinoma" or "SCLC").tw,kf.	154842
2	Small Cell Lung Carcinoma/	12126
3	Carcinoma, Small Cell/	10486
4	Lung Neoplasms/	33012
5	3 and 4	1070
6	1 or 2 or 5	159259
7	(("prophyla*" and ("crani*" or "intracrani*" or "brain" or "CNS") and ("radiation" or "irradiation" or "radiotherapy")) or "PCI").tw,kf.	78643
8	Brain Neoplasms/	45489
9	Central Nervous System Neoplasms/	8220
10	exp Brain/	1762478
11	8 or 9 or 10	1805470
12	exp Neoplasm Metastasis/	847214
13	11 and 12	20870
14	exp Lung Diseases/ or exp Lung/	2400069
15	exp Neoplasms/	6045083
16	14 and 15	754089
17	3 and 16	6362
18	6 or 17	163007
19	exp Radiotherapy/	735269
20	13 and 19	6335
21	7 or 20	84680
22	18 and 21	2760

List of included studies

Ago et al., 2002[1]
Ahn et al., 2018[2]
Ainsworth, 2011[3]
Akyurek et al., 2006[4]
Al Farsi et al., 2017[5]
Alexopoulos et al., 1997[6]
Alexopoulos et al., 1997
Alexopoulos et al., 1997
An et al., 2017[7]
Aroney et al., 1983[8]
Arriagada et al., 1995[9]
Arriagada et al., 2002[10]
Asaad et al., 2018[11]
Atci et al., 2021[12]
Aufderstrasse et al., 2018[13]
Ayala de Miguel et al., 2020[14]
Aynaci et al., 2016[15]

Bains et al., 2001[16]
Bang et al., 2018[17]
Bayman et al., 2014[18]
Bettington et al., 2013[19]
Bischof et al., 2007[20]
Boskovic et al., 2019[21]
Bravo et al., 2019[22]
Bruni et al., 2015[23]

Cai et al., 2014[24]
Cao et al., 2005[25]
Cetingoz et al., 2006[26]
Chen et al., 2014[27]
Chen et al., 2016[28]
Chen et al., 2016[29]
Chen et al., 2018[30]
Chen et al., 2021[31]
Chen et al., 2022[32]
Chen et al., 2022[33]
Choi et al., 2017[34]
Chudyba et al., 2018[35]
Chung et al., 2020[36]
Cimen et al., 2022[37]
Clark et al., 2010[38]
Cordeiro et al., 2019[39]
Cox et al., 1978[40]

Dawe et al., 2022[41]
Dawe et al., 2023[42]
De Almeida et al., 2021[43]
Depierre et al., 1997[44]
Devisetty et al., 2011[45]
Drpa et al., 2020[46]

Eagan et al., 1981[47]

Eaton et al., 2013[48]
Elegbede et al. 2020[49]
Elegbede et al., 2021[50]
Ergen et al., 2017[51]
Eze et al., 2016[52]

Faramand et al., 2019[53]
Farooqi et al., 2017[54]
Farris et al., 2023[55]
Fleck et al., 1990[56]
Frytak et al., 1989[57]

Geddes et al., 1990[58]
Ghanta et al., 2021[59]
Giaccone et al., 2005[60]
Go et al., 2014[61]
Gross et al., 2020[62]
Gregor et al., 1997[63]
Guiliani et al., 2010[64]
Guo et al., 2020[65]

Han et al., 2018[66]
Harden et al., 2011[67]
Held et al., 2021[68]
Herrmann et al., 2011[69]
Hett et al., 2014[70]
Hu et al., 2020[71]
Hu et al., 2022[72]
Hwang et al., 2017[73]

Inoue et al., 2021[74]
Iqbal et al., 2020[75]

Jacobs et al., 1986[76]
Janardanan Nair et al., 2012[77]
Jat et al., 2019[78]
Jing et al., 2017[79]
Jo et al., 2011[80]
Jo et al., 2017[81]
Jones et al., 2021[82]
Jove et al., 2011[83]

Kamath et al., 1998[84]
Kang et al., 2022 [85]
Kasmann et al., 2016[86]
Kasmann et al., 2017[87]
Kasmann et al., 2017[88]
Keller et al., 2020[89]
Keller et al., 2021[90]
Khaira et al., 2010[91]
Khanfir et al., 2011[92]
Kim et al., 2016[93]
Kim et al., 2019[94]
Koh et al., 2019[95]
Komaki et al., 1983[96]
Komaki et al., 2015[97]

Komaki et al., 2016[98]
Kosmidis et al., 1994[99]
Kou et al., 2018[100]

Lee et al., 2023[101]
Levy et al., 2018[102]
Lewiński et al., 1990[103]
Li et al., 2010[104]
Li et al., 2021[105]
Liengswangwong et al., 1995[106]
Lim et al., 2022[107]
Lim et al., 2007[108]
Lin et al., 2020[109]
Lishner et al., 1990[110]
Liu et al., 1993[111]
Liu et al., 2018[112]
Lok et al., 2017[113]
Longo et al., 2022[114]
Ludbrook et al., 2003[115]

Ma et al., 2021[116]
MacDonald et al., 2009[117]
Mamesaya et al., 2018[118]
Manopov et al., 2012[119]
Matutino et al., 2018[120]

Nakahara et al, 2015[121]
Nakamura et al., 2018[122]
Naidoo et al., 2018[123]
Ng et al., 2007[124]
Nicholls et al., 2016[125]
Niiranen et al., 1989[126]

Ohonoshi et al., 1993[127]
Ozawa et al., 2015[128]

Park et al., 2022[129]
Park et al., 2023[130]
Patel et al., 2013[131]
Pezzi et al., 2020[132]
Prelaj et al., 2017[133]

Qi et al., 2022[134]
Qiu et al., 2016[135]

Resio et al., 2019[136]
Rosen et al., 1983[137]
Rosenstein et al., 1992[138]
Rubenstein et al., 1995[139]
Rule et al., 2014[140]

Sakin et al., 2019[141]
Salama et al., 2016[142]
Sas-Korczynska et al., 2010[143]
Schnoller et al., 2021[144]
Scotti et al., 2014[145]

Sculier et al., 1987[146]
Seydel et al., 1985[147]
Sharma et al., 2018[148]
Shaw et al., 1994[149]
Sheikh et al., 2021[150]
Shioyama et al., 2015[151]
Shirvani et al., 2012[152]
Simo et al., 2016[153]
Siker et al., 2011[154]
Slotman et al., 1993[155]
Slotman et al., 2009[156]
Soon et al., 2017[157]
Souhami & Law, 1990[158]
Stanic et al., 2020[159]
Stolten et al., 2020[160]
Sun et al., 2021[161]
Suzuki et al., 2018[162]

Takahashi et al., 2017[163]
Takamura et al., 2015[164]
Tai et al., 2013[165]
Tendler et al., 2019[166]
Teng et al., 2019[167]
Teng et al., 2021[168]
Truong et al., 2003[169]
Turaka et al., 2013[170]
Twijnstra et al., 1987[171]
Twijnstra et al., 1996[172]
Twijnstra et al., 1997[173]

Ueoka et al., 1990[174]
Ueki et al., 2022[175]
Ulsperger et al., 1991[176]
Uprety et al., 2019[177]

Van der Linden et al., 2001[178]
Veena et al., 2023[179]

Wang et al., 2015[180]
Wang et al., 2016[181]
Wang et al., 2020[182]
Wei et al., 2014[183]
Wei et al., 2016[184]
Wei et al., 2022[185]
Wheless et al., 2019[186]
Wierzchowski et al., 2009[187]
Winther-Larsen et al., 2015[188]
Work et al., 1996[189]
Wu et al., 2017[190]
Wu et al., 2015[191]
Wu et al., 2023[192]
Wzietek et al., 2014[193]

Xanthopoulos et al., 2013[194]
Xiaodong et al., 2019[195]
Xingwen et al., 2021[196]

Xu et al., 2017[197]
Xu-Yi et al., 1994[198]

Yan et al., 2021[199]
Yang et al., 2020[200]
Yao et al., 2023[201]
Yavaş et al., 2022[202]
Yildirim et al., 2015[203]
Yilmaz et al., 2020[204]
Yin et al., 2018[205]
Yokouchi et al., 2015[206]
Yu et al., 2019[207]
Yu et al., 2020[208]
Yu et al., 2021[209]
Yu et al., 2022[210]
Yuqiong et al., 2021[211]

Zahra et al., 2016[212]
Zemanova et al., 2011[213]
Zhang et al., 2017[214]
Zhang et al., 2019[215]
Zhang et al., 2019[216]
Zhang et al., 2022[217]
Zhou et al., 2017[218]
Zhou et al., 2021[219]
Zhu et al., 2014[220]
Zhuang et al., 2015[221]
Ziegler et al., 2022[222]
Zongmei et al., 2015[223]

Primary outcome: overall survival

Subgroup and heterogeneity analysis

Pre-specified subgroup analyses were performed on all included studies as well as separated by studies consisting exclusively of patients with limited and extensive stage small cell lung cancer (Table 1). Meta-regression was performed on publication year, total study sample size, and median age of participants. Neither predictor led to a significant reduction in study heterogeneity when considering all included studies (p=0·09, p=0·8180, and p=0·1516, respectively), or when investigated separately for limited (p=0·6455, p=0·3814, and p=0·08, respectively) or extensive stage patients (p=0·06, p=0·86, and p=0·6451, respectively).

Of all studies included in the primary analysis, 39 studies reported unadjusted hazard ratios from univariable analysis only, 17 studies reported adjusted hazard ratios from multivariable analysis only, and 56 reported findings in unadjusted and adjusted hazard ratios. Post-hoc sensitivity analyses of unadjusted and adjusted hazard ratios only found prophylactic cranial irradiation (PCI) associated with longer survival (unadjusted HR 0·60; 95% CI, 0·55-0·65; p<0·001; n=52,928 patients; adjusted HR 0·59; 95% CI, 0·55-0·64; p<0·001; n=32,879 patients; Figure S1, S2). Further sensitivity analysis where adjusted HRs were substituted for unadjusted HRs also confirmed these findings (HR 0·62; 95% CI, 0·58-0·67; p<0·001; n=105 studies; n=56,770 patients; Figure S3).

Between-study heterogeneity was significant when pooled amongst all studies (I^2=73·6%; 95% CI 68·4%-77·9%). Subgroup analysis did not reveal sources of heterogeneity. Effect size analysis did not identify any individual studies that contributed disproportionally to overall between-study heterogeneity in the overall study cohort (Figure S4) or when focusing exclusively on studies that used brain MRI to exclude presence of brain metastases among all patients (results not shown).

Egger's test did not suggest any evidence of publication bias (intercept -0.342, 95% CI, -1- -0·16, t=-1·414, p=0·160, Figure S5). Results from evaluation of risk of bias are reported (Figure S6, S7).

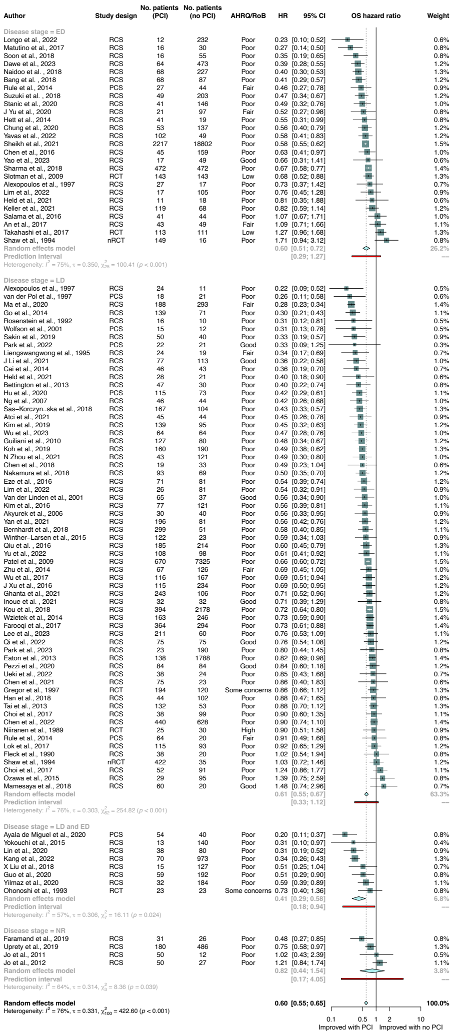

Figure S1. Random-effects meta-analysis of PCI versus no PCI for the primary outcome of overall survival using only unadjusted hazard ratios.

Author	Study design	No. patients (PCI)	No. patients (no PCI)	AHRQ/RoB	HR	95% CI	OS hazard ratio	Weight
**Disease stage = ED**								
Soon et al., 2018	RCS	16	55	Poor	0.22	[0.10; 0.48]		0.7%
Longo et al., 2022	RCS	12	232	Poor	0.29	[0.12; 0.70]		0.6%
Elegbede et al., 2020	RCS	47	193	Poor	0.48	[0.33; 0.70]		1.6%
Zhou et al. , 2017	RCS	33	350	Poor	0.50	[0.32; 0.76]		1.4%
Bang et al. , 2018	RCS	68	87	Poor	0.56	[0.40; 0.78]		1.7%
Suzuki et al. , 2018	RCS	49	203	Poor	0.60	[0.42; 0.85]		1.7%
Sheikh et al., 2022*	RCS	1674	3070	Poor	0.60	[0.57; 0.64]		2.6%
Ma et al., 2020	RCS	29	478	Fair	0.61	[0.37; 1.03]		1.2%
Naidoo et al. , 2018	RCS	68	227	Poor	0.63	[0.44; 0.91]		1.6%
J Yu et al., 2020	RCS	21	97	Fair	0.63	[0.30; 1.33]		0.7%
Sharma et al., 2018	RCS	472	472	Poor	0.66	[0.59; 0.73]		2.5%
Yao et al., 2023	RCS	17	49	Good	0.68	[0.31; 1.49]		0.7%
Chen et al., 2016	RCS	45	159	Poor	0.68	[0.44; 1.05]		1.4%
Chung et al., 2020*	RCS	48	48	Poor	0.70	[0.49; 1.00]		1.6%
Keller et al., 2021*	RCS	57	57	Poor	0.90	[0.64; 1.27]		1.7%
Stanic et al., 2020	RCS	41	146	Poor	1.59	[1.09; 2.32]		1.6%
**Random effects model**					0.63	[0.51; 0.77]		23.3%
**Prediction interval**						[0.34; 1.17]		--
Heterogeneity: $I^2 = 66\%$, $\tau = 0.275$, $\chi^2_{15} = 44.69$ ($p < 0.001$)								
**Disease stage = LD**								
Park et al., 2022	PCS	22	21	Good	0.24	[0.05; 1.21]		0.2%
Kasmann et al., 2017	RCS	6	2	Poor	0.29	[0.15; 0.59]		0.8%
Chen et al., 2018	RCS	19	33	Poor	0.33	[0.11; 0.95]		0.4%
Schnoller et al., 2021	RCS	24	9	Poor	0.36	[0.12; 1.05]		0.4%
Go et al., 2014	RCS	139	71	Poor	0.36	[0.24; 0.54]		1.5%
Ma et al., 2020	RCS	188	293	Fair	0.37	[0.31; 0.45]		2.3%
Chen et al., 2016	RCS	109	68	Poor	0.39	[0.27; 0.57]		1.6%
Ka..smann et al., 2017	RCS	47	18	Poor	0.39	[0.19; 0.82]		0.7%
Ng et al., 2007	RCS	46	44	Poor	0.40	[0.24; 0.65]		1.2%
Lim et al., 2022	RCS	26	81	Poor	0.41	[0.22; 0.77]		0.9%
Zhu et al., 2014	RCS	67	126	Fair	0.43	[0.26; 0.71]		1.2%
Hu et al., 2020	PCS	115	73	Poor	0.43	[0.29; 0.63]		1.6%
Winther–Larsen et al., 2015	RCS	122	23	Poor	0.43	[0.24; 0.78]		1.0%
Sakin et al., 2019	RCS	50	40	Poor	0.45	[0.25; 0.81]		1.0%
Bettington et al., 2013	RCS	47	30	Poor	0.45	[0.24; 0.88]		0.9%
Elegbede et al., 2020	RCS	60	60	Poor	0.48	[0.29; 0.79]		1.2%
Guiliani et al., 2010	RCS	127	80	Poor	0.50	[0.30; 0.83]		1.2%
Held et al., 2021	RCS	28	21	Poor	0.51	[0.21; 1.26]		0.5%
Giaccone et al., 2005	RCS	319	196	Poor	0.52	[0.41; 0.65]		2.1%
Eze et al. , 2016	RCS	71	81	Poor	0.53	[0.38; 0.73]		1.8%
Zhang et al., 2017	RCS	94	76	Poor	0.53	[0.35; 0.80]		1.5%
Yan et al., 2021	RCS	196	81	Poor	0.53	[0.37; 0.76]		1.7%
Jeong et al., 2018	RCS	56	45	Poor	0.53	[0.34; 0.85]		1.3%
Hu et al., 2022	RCS	88	52	Fair	0.54	[0.36; 0.80]		1.5%
Nakamura et al., 2018	RCS	93	69	Poor	0.54	[0.36; 0.81]		1.5%
Sas–Korczyn..ska et al., 2018	RCS	167	104	Poor	0.55	[0.42; 0.73]		1.9%
Kim et al., 2016	RCS	77	121	Poor	0.60	[0.41; 0.89]		1.5%
Videtic et al., 2003	RCS	48	166	Poor	0.63	[0.44; 0.91]		1.6%
Bernhardt et al., 2018	RCS	299	51	Poor	0.65	[0.44; 0.96]		1.6%
Qiu et al. , 2016	RCS	185	214	Poor	0.66	[0.49; 0.87]		1.9%
Yin et al., 2018	RCS	160	109	Poor	0.67	[0.49; 0.90]		1.9%
Wu et al., 2017	RCS	116	167	Poor	0.67	[0.49; 0.92]		1.8%
Yu et al., 2022	RCS	108	98	Poor	0.68	[0.45; 1.05]		1.4%
J Xu et al., 2016	RCS	115	234	Poor	0.69	[0.50; 0.95]		1.8%
Resio et al. , 2019	RCS	202	657	Poor	0.70	[0.55; 0.89]		2.1%
Zahra et al., 2016	RCS	16	22	Fair	0.71	[0.32; 1.58]		0.7%
Eaton et al., 2013	RCS	138	1788	Poor	0.72	[0.53; 0.97]		1.9%
Ghanta et al., 2021*	RCS	243	106	Poor	0.74	[0.49; 1.11]		1.5%
Zhou et al. , 2017	RCS	33	107	Poor	0.74	[0.45; 1.23]		1.2%
Qi et al., 2022	RCS	75	75	Good	0.76	[0.53; 1.09]		1.7%
Farooqi et al., 2017	RCS	364	294	Poor	0.76	[0.63; 0.91]		2.3%
Kou et al., 2018	RCS	394	2178	Poor	0.76	[0.68; 0.85]		2.5%
N Zhou et al., 2021	RCS	43	121	Poor	0.78	[0.41; 1.49]		0.9%
Pezzi et al., 2020*	RCS	84	84	Good	0.79	[0.56; 1.11]		1.7%
Atci et al., 2021	RCS	45	44	Poor	0.89	[0.24; 3.27]		0.3%
Choi et al., 2017	RCS	90	190	Poor	0.92	[0.67; 1.27]		1.8%
Patel et al., 2009	RCS	670	7325	Poor	0.93	[0.88; 0.99]		2.6%
Aynaci et al., 2016	RCS	41	88	Poor	1.00	[0.63; 1.58]		1.3%
Mamesaya et al., 2018	RCS	60	20	Good	1.29	[0.48; 3.47]		0.5%
**Random effects model**					0.59	[0.55; 0.65]		68.3%
**Prediction interval**						[0.38; 0.92]		--
Heterogeneity: $I^2 = 80\%$, $\tau = 0.217$, $\chi^2_{48} = 234.83$ ($p < 0.001$)								
**Disease stage = LD and ED**								
Ayala de Miguel et al., 2020	PCS	54	40	Poor	0.18	[0.08; 0.39]		0.7%
Tendler et al. , 2019	RCS	52	49	Poor	0.27	[0.07; 1.08]		0.3%
Yokouchi et al., 2015	RCS	13	140	Poor	0.28	[0.08; 0.94]		0.3%
Lin et al., 2020	RCS	38	80	Poor	0.33	[0.19; 0.56]		1.1%
Guo et al., 2020	RCS	59	192	Poor	0.43	[0.24; 0.77]		1.0%
Drpa et al., 2020	RCS	12	128	Poor	0.49	[0.21; 1.12]		0.6%
Kang et al., 2022	RCS	70	973	Poor	0.61	[0.40; 0.91]		1.5%
Rule et al., 2014	PCS	91	64	Fair	0.74	[0.48; 1.15]		1.4%
Yilmaz et al., 2020	RCS	32	184	Poor	0.75	[0.49; 1.15]		1.4%
**Random effects model**					0.46	[0.32; 0.67]		8.4%
**Prediction interval**						[0.18; 1.19]		--
Heterogeneity: $I^2 = 57\%$, $\tau = 0.367$, $\chi^2_{8} = 18.43$ ($p = 0.018$)								
**Random effects model**					**0.59**	**[0.55; 0.64]**		**100.0%**
Heterogeneity: $I^2 = 78\%$, $\tau = 0.233$, $\chi^2_{73} = 338.49$ ($p < 0.001$)								

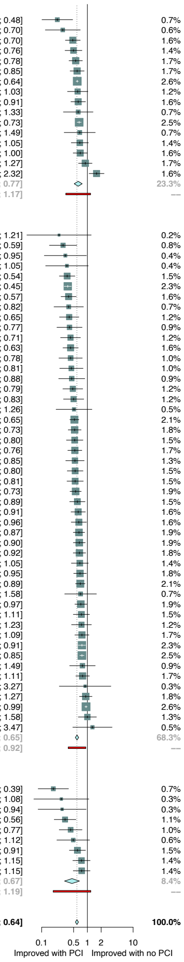

Figure S2. Random-effects meta-analysis of PCI versus no PCI for the primary outcome of overall survival using only adjusted hazard ratios.

Author	Study design	No. patients (PCI)	No. patients (no PCI)	AHRQ/RoB	HR	95% CI	Weight
**Disease stage = ED**							
Soon et al., 2018	RCS	16	55	Poor	0.22	[0.10; 0.48]	0.5%
Matutino et al., 2017	RCS	16	30	Poor	0.27	[0.14; 0.50]	0.6%
Longo et al., 2022	RCS	12	232	Poor	0.29	[0.12; 0.70]	0.4%
Dawe et al., 2023	RCS	64	473	Poor	0.39	[0.28; 0.55]	1.1%
Lim et al., 2022	RCS	17	105	Poor	0.41	[0.22; 0.77]	0.6%
Rule et al., 2014	PCS	27	44	Fair	0.46	[0.27; 0.78]	0.8%
Elegbede et al., 2020	RCS	47	193	Poor	0.48	[0.33; 0.70]	1.0%
Zhou et al. , 2017	RCS	33	350	Poor	0.50	[0.32; 0.76]	0.9%
J Yu et al., 2020	RCS	21	97	Fair	0.52	[0.27; 0.98]	0.6%
Hett et al., 2014	RCS	41	19	Poor	0.55	[0.31; 0.99]	0.7%
Bang et al. , 2018	RCS	68	87	Poor	0.56	[0.40; 0.78]	1.1%
Yavas et al., 2022	RCS	102	49	Poor	0.58	[0.41; 0.83]	1.0%
Suzuki et al. , 2018	RCS	49	203	Poor	0.60	[0.42; 0.85]	1.0%
Sheikh et al., 2021	RCS	2217	18802	Poor	0.60	[0.57; 0.64]	1.4%
Naidoo et al. , 2018	RCS	68	227	Poor	0.63	[0.44; 0.91]	1.0%
Sharma et al., 2018	RCS	472	472	Poor	0.66	[0.59; 0.73]	1.4%
Yao et al., 2023	RCS	17	49	Good	0.68	[0.31; 1.49]	0.5%
Slotman et al., 2009	RCT	143	143	Low	0.68	[0.52; 0.88]	1.2%
Chen et al., 2016	RCS	45	159	Poor	0.68	[0.44; 1.05]	0.9%
Chung et al., 2020	RCS	53	137	Poor	0.70	[0.49; 1.00]	1.0%
Alexopoulos et al., 1997	RCS	27	17	Poor	0.73	[0.37; 1.42]	0.6%
Held et al., 2021	RCS	11	18	Poor	0.89	[0.24; 3.27]	0.2%
Keller et al., 2021	RCS	119	68	Poor	0.90	[0.64; 1.27]	1.1%
Salama et al., 2016	RCS	41	44	Poor	1.07	[0.67; 1.71]	0.9%
An et al., 2017	RCS	43	49	Fair	1.09	[0.71; 1.66]	0.9%
Takahashi et al., 2017	RCT	113	111	Low	1.27	[0.96; 1.68]	1.2%
Stanic et al., 2020	RCS	41	146	Poor	1.59	[1.09; 2.32]	1.0%
Shaw et al., 1994	nRCT	149	16	Poor	1.71	[0.94; 3.12]	0.7%
**Random effects model**					0.64	[0.54; 0.77]	24.3%
**Prediction interval**						[0.29; 1.42]	--
Heterogeneity: $I^2 = 76\%, \tau = 0.374, \chi^2_{27} = 110.96$ ($p < 0.001$)							
**Disease stage = LD**							
Alexopoulos et al., 1997	RCS	24	11	Poor	0.22	[0.09; 0.52]	0.4%
Park et al., 2022	PCS	22	21	Good	0.24	[0.05; 1.21]	0.2%
van der Pol et al., 1997	PCS	18	21	Poor	0.26	[0.11; 0.58]	0.5%
Kasmann et al., 2017	RCS	6	2	Poor	0.29	[0.15; 0.59]	0.6%
Rosenstein et al., 1992	RCS	16	10	Poor	0.31	[0.12; 0.81]	0.4%
Wolfson et al., 2001	PCS	15	12	Poor	0.31	[0.13; 0.78]	0.4%
Chen et al., 2018	RCS	19	33	Poor	0.33	[0.11; 0.95]	0.3%
Liengswangwong et al., 1995	RCS	24	19	Fair	0.34	[0.17; 0.69]	0.6%
Schnoller et al., 2021	RCS	24	9	Poor	0.36	[0.12; 1.05]	0.3%
J Li et al., 2021	RCS	77	113	Good	0.36	[0.22; 0.58]	0.8%
Cai et al., 2014	RCS	46	43	Poor	0.36	[0.19; 0.70]	0.6%
Go et al., 2014	RCS	139	71	Poor	0.36	[0.24; 0.54]	1.0%
Ma et al., 2020	RCS	188	293	Fair	0.37	[0.31; 0.45]	1.3%
Chen et al., 2016	RCS	109	68	Poor	0.39	[0.27; 0.57]	1.0%
Ka..smann et al., 2017	RCS	47	18	Poor	0.39	[0.19; 0.82]	0.5%
Ng et al., 2007	RCS	46	44	Poor	0.40	[0.24; 0.65]	0.8%
Zhu et al., 2014	RCS	67	126	Fair	0.43	[0.26; 0.71]	0.8%
Hu et al., 2020	PCS	115	73	Poor	0.43	[0.29; 0.63]	1.0%
Winther–Larsen et al., 2015	RCS	122	23	Poor	0.43	[0.24; 0.78]	0.7%
Sakin et al., 2019	RCS	50	40	Poor	0.45	[0.25; 0.81]	0.7%
Kim et al., 2019	RCS	139	95	Poor	0.45	[0.32; 0.63]	1.1%
Bettington et al., 2013	RCS	47	30	Poor	0.45	[0.24; 0.88]	0.6%
Wu et al., 2023	RCS	64	64	Poor	0.47	[0.28; 0.76]	0.8%
Elegbede et al., 2020	RCS	60	60	Poor	0.48	[0.29; 0.79]	0.8%
Koh et al., 2019	RCS	160	190	Poor	0.49	[0.38; 0.62]	1.2%
Guiliani et al., 2010	RCS	127	80	Poor	0.50	[0.30; 0.83]	0.8%
Inoue et al. , 2021	RCS	32	32	Good	0.51	[0.21; 1.26]	0.4%
Giaccone et al., 2005	RCS	319	196	Poor	0.52	[0.41; 0.65]	1.2%
Eze et al. , 2016	RCS	71	81	Poor	0.53	[0.38; 0.73]	1.1%
Zhang et al., 2017	RCS	94	76	Poor	0.53	[0.35; 0.80]	0.9%
Yan et al., 2021	RCS	196	81	Poor	0.53	[0.37; 0.76]	1.0%
Jeong et al., 2018	RCS	56	45	Poor	0.53	[0.34; 0.85]	0.9%
Hu et al., 2022	RCS	88	52	Fair	0.54	[0.36; 0.80]	1.0%
Nakamura et al., 2018	RCS	93	69	Poor	0.54	[0.36; 0.81]	0.9%
Sas–Korczyn..ska et al., 2018	RCS	167	104	Poor	0.55	[0.42; 0.73]	1.1%
Van der Linden et al., 2001	RCS	65	37	Good	0.56	[0.34; 0.90]	0.8%
Akyurek et al., 2006	RCS	30	40	Poor	0.56	[0.33; 0.95]	0.8%
Kim et al., 2016	RCS	77	121	Poor	0.60	[0.41; 0.89]	1.0%
Lim et al., 2022	RCS	26	81	Poor	0.61	[0.37; 1.03]	0.8%
Videtic et al., 2003	RCS	48	166	Poor	0.63	[0.44; 0.91]	1.0%
Bernhardt et al., 2018	RCS	299	51	Poor	0.65	[0.44; 0.96]	1.0%
Qiu et al. , 2016	RCS	185	214	Poor	0.66	[0.49; 0.87]	1.1%
Yin et al., 2018	RCS	160	109	Poor	0.67	[0.49; 0.90]	1.1%
Wu et al., 2017	RCS	116	167	Poor	0.67	[0.49; 0.92]	1.1%
Yu et al., 2022	RCS	108	98	Poor	0.68	[0.45; 1.05]	0.9%
J Xu et al., 2016	RCS	115	234	Poor	0.69	[0.50; 0.95]	1.1%
Resio et al. , 2019	RCS	202	657	Poor	0.70	[0.55; 0.89]	1.2%
Zahra et al., 2016	RCS	16	22	Fair	0.71	[0.32; 1.58]	0.5%
Eaton et al., 2013	RCS	138	1788	Poor	0.72	[0.53; 0.97]	1.1%
Wzietek et al., 2014	RCS	163	246	Poor	0.73	[0.59; 0.90]	1.3%
Held et al., 2021	RCS	28	21	Poor	0.74	[0.49; 1.11]	0.9%
Ghanta et al., 2021	RCS	243	106	Poor	0.74	[0.49; 1.11]	0.9%
Zhou et al. , 2017	RCS	33	107	Poor	0.74	[0.45; 1.23]	0.8%
Lee et al., 2023	RCS	211	60	Poor	0.76	[0.53; 1.09]	1.0%
Qi et al., 2022	RCS	75	75	Good	0.76	[0.53; 1.09]	1.0%
Farooqi et al., 2017	RCS	364	294	Poor	0.76	[0.63; 0.91]	1.3%
Kou et al., 2018	RCS	394	2178	Poor	0.76	[0.68; 0.85]	1.4%
N Zhou et al., 2021	RCS	43	121	Poor	0.78	[0.41; 1.49]	0.6%
Pezzi et al., 2020	RCS	84	84	Good	0.79	[0.56; 1.11]	1.0%
Park et al., 2023	RCS	23	190	Poor	0.80	[0.44; 1.45]	0.7%
Ueki et al., 2022	RCS	38	24	Poor	0.85	[0.43; 1.68]	0.6%
Chen et al., 2021	RCS	75	23	Poor	0.86	[0.40; 1.83]	0.5%
Gregor et al., 1997	RCT	194	120	Some concerns	0.86	[0.66; 1.12]	1.2%
Han et al., 2018	RCS	44	102	Poor	0.88	[0.47; 1.65]	0.6%
Tai et al., 2013	RCS	132	53	Poor	0.88	[0.70; 1.12]	1.2%
Atci et al., 2021	RCS	45	44	Poor	0.89	[0.24; 3.27]	0.2%
Choi et al., 2017	RCS	38	99	Poor	0.90	[0.60; 1.35]	1.0%
Chen et al., 2022	RCS	440	628	Poor	0.90	[0.74; 1.10]	1.3%
Niiranen et al., 1989	RCT	25	30	High	0.90	[0.51; 1.58]	0.7%
Rule et al., 2014	PCS	64	20	Fair	0.91	[0.49; 1.68]	0.7%
Lok et al., 2017	RCS	115	93	Poor	0.92	[0.65; 1.29]	1.1%
Patel et al., 2009	RCS	670	7325	Poor	0.93	[0.88; 0.99]	1.4%
Aynaci et al., 2016	RCS	41	88	Poor	1.00	[0.63; 1.58]	0.9%
Fleck et al., 1990	RCS	38	20	Poor	1.02	[0.54; 1.94]	0.6%
Shaw et al., 1994	nRCT	422	35	Poor	1.03	[0.72; 1.46]	1.0%
Choi et al., 2017	RCS	52	91	Poor	1.24	[0.86; 1.77]	1.0%
Mamesaya et al., 2018	RCS	60	20	Good	1.29	[0.48; 3.47]	0.4%
Ozawa et al., 2015	RCS	29	95	Poor	1.39	[0.75; 2.59]	0.7%
**Random effects model**					0.62	[0.57; 0.67]	66.4%
**Prediction interval**						[0.37; 1.04]	--
Heterogeneity: $I^2 = 76\%, \tau = 0.253, \chi^2_{77} = 321.5$ ($p < 0.001$)							
**Disease stage = LD and ED**							
Ayala de Miguel et al., 2020	PCS	54	40	Poor	0.18	[0.08; 0.39]	0.5%
Tendler et al. , 2019	RCS	52	49	Poor	0.27	[0.07; 1.08]	0.2%
Yokouchi et al., 2015	RCS	13	140	Poor	0.28	[0.08; 0.94]	0.3%
Lin et al., 2020	RCS	38	80	Poor	0.33	[0.19; 0.56]	0.7%
Guo et al., 2020	RCS	59	192	Poor	0.43	[0.24; 0.77]	0.7%
Drpa et al., 2020	RCS	12	128	Poor	0.49	[0.21; 1.12]	0.5%
X Liu et al., 2018	RCS	15	127	Poor	0.51	[0.25; 1.04]	0.6%
Kang et al., 2022	RCS	70	973	Poor	0.61	[0.40; 0.91]	0.9%
Ohonoshi et al., 1993	RCT	23	23	Some concerns	0.73	[0.40; 1.36]	0.7%
Yilmaz et al., 2020	RCS	32	184	Poor	0.75	[0.49; 1.15]	0.9%
**Random effects model**					0.46	[0.33; 0.64]	6.0%
**Prediction interval**						[0.21; 1.03]	--
Heterogeneity: $I^2 = 47\%, \tau = 0.313, \chi^2_{9} = 16.94$ ($p = 0.050$)							
**Disease stage = NR**							
Faramand et al., 2019	RCS	31	26	Poor	0.48	[0.27; 0.85]	0.7%
Uprety et al., 2019	RCS	180	486	Poor	0.75	[0.58; 0.97]	1.2%
Jo et al., 2011	RCS	50	12	Poor	1.02	[0.43; 2.39]	0.4%
Jo et al., 2012	RCS	50	27	Poor	1.21	[0.84; 1.74]	1.0%
**Random effects model**					0.82	[0.44; 1.54]	3.3%
**Prediction interval**						[0.17; 4.05]	--
Heterogeneity: $I^2 = 64\%, \tau = 0.314, \chi^2_{3} = 8.36$ ($p = 0.039$)							
**Random effects model**					**0.62**	**[0.58; 0.67]**	**100.0%**
Heterogeneity: $I^2 = 76\%, \tau = 0.291, \chi^2_{119} = 501.07$ ($p < 0.001$)							

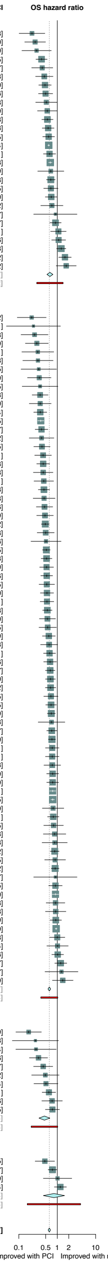

Figure S3. Random-effects meta-analysis of PCI versus no PCI for the primary outcome of overall survival using adjusted instead of unadjusted hazard ratios where available.

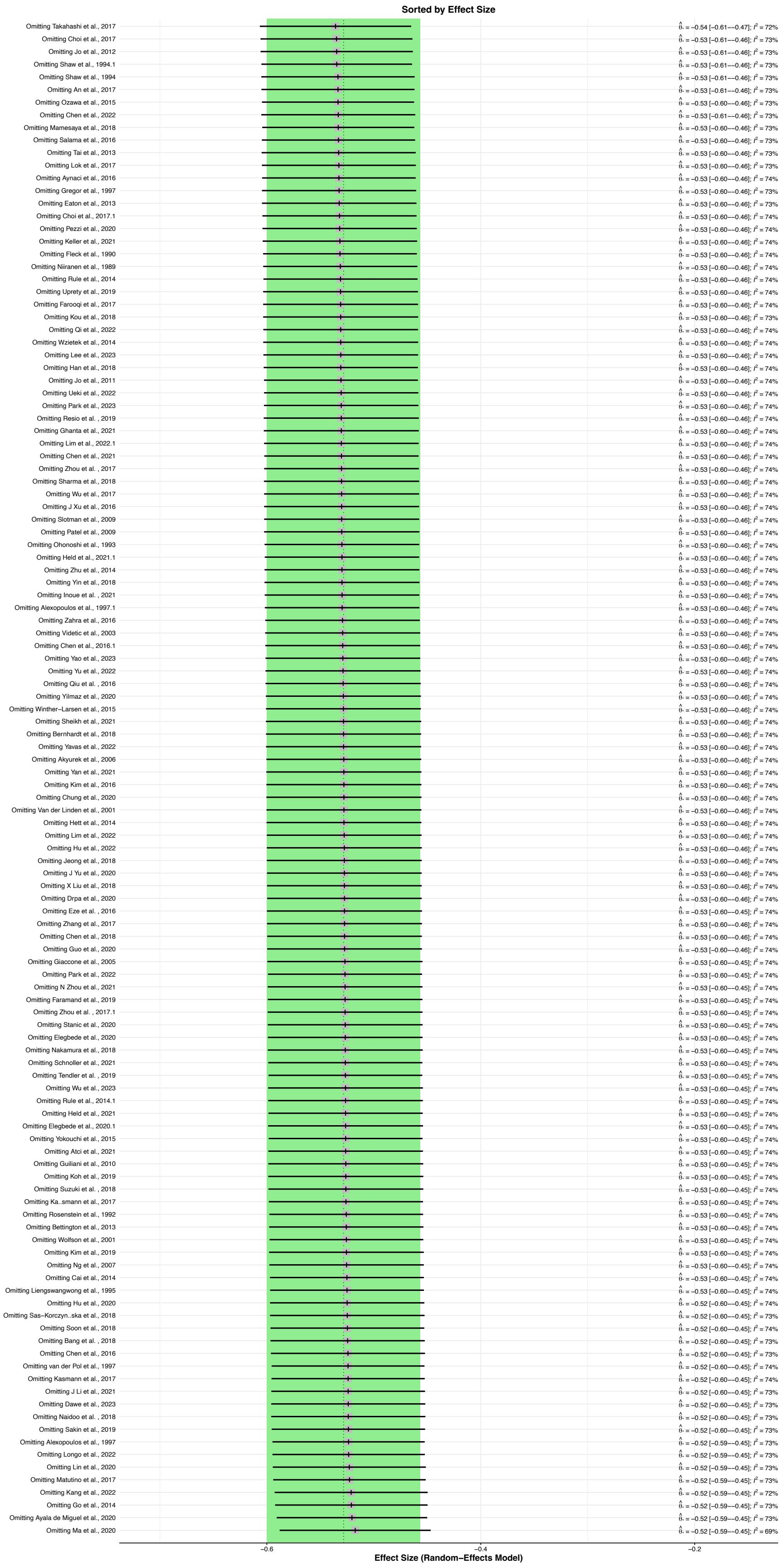
Sorted by Effect Size
Omitting Takahashi et al., 2017
Omitting Choi et al., 2017
Omitting Jo et al., 2012
Omitting Shaw et al., 1994.1
Omitting Shaw et al., 1994
Omitting An et al., 2017
Omitting Ozawa et al., 2015
Omitting Chen et al., 2022
Omitting Mamesaya et al., 2018
Omitting Salama et al., 2016
Omitting Tai et al., 2013
Omitting Lok et al., 2017
Omitting Aynaci et al., 2016
Omitting Gregor et al., 1997
Omitting Eaton et al., 2013
Omitting Choi et al., 2017.1
Omitting Pezzi et al., 2020
Omitting Keller et al., 2021
Omitting Fleck et al., 1990
Omitting Niiranen et al., 1989
Omitting Rule et al., 2014
Omitting Uprety et al., 2019
Omitting Farooqi et al., 2017
Omitting Kou et al., 2018
Omitting Qi et al., 2022
Omitting Wzietek et al., 2014
Omitting Lee et al., 2023
Omitting Han et al., 2018
Omitting Jo et al., 2011
Omitting Ueki et al., 2022
Omitting Park et al., 2023
Omitting Resio et al. , 2019
Omitting Ghanta et al., 2021
Omitting Lim et al., 2022.1
Omitting Chen et al., 2021
Omitting Zhou et al. , 2017
Omitting Sharma et al., 2018
Omitting Wu et al., 2017
Omitting J Xu et al., 2016
Omitting Slotman et al., 2009
Omitting Patel et al., 2009
Omitting Ohonoshi et al., 1993
Omitting Held et al., 2021.1
Omitting Zhu et al., 2014
Omitting Yin et al., 2018
Omitting Inoue et al. , 2021
Omitting Alexopoulos et al., 1997.1
Omitting Zahra et al., 2016
Omitting Videtic et al., 2003
Omitting Chen et al., 2016.1
Omitting Yao et al., 2023
Omitting Yu et al., 2022
Omitting Qiu et al. , 2016
Omitting Yilmaz et al., 2020
Omitting Winther−Larsen et al., 2015
Omitting Sheikh et al., 2021
Omitting Bernhardt et al., 2018
Omitting Yavas et al., 2022
Omitting Akyurek et al., 2006
Omitting Yan et al., 2021
Omitting Kim et al., 2016
Omitting Chung et al., 2020
Omitting Van der Linden et al., 2001
Omitting Hett et al., 2014
Omitting Lim et al., 2022
Omitting Hu et al., 2022
Omitting Jeong et al., 2018
Omitting J Yu et al., 2020
Omitting X Liu et al., 2018
Omitting Drpa et al., 2020
Omitting Eze et al. , 2016
Omitting Zhang et al., 2017
Omitting Chen et al., 2018
Omitting Guo et al., 2020
Omitting Giaccone et al., 2005
Omitting Park et al., 2022
Omitting N Zhou et al., 2021
Omitting Faramand et al., 2019
Omitting Zhou et al. , 2017.1
Omitting Stanic et al., 2020
Omitting Elegbede et al., 2020
Omitting Nakamura et al., 2018
Omitting Schnoller et al., 2021
Omitting Tendler et al. , 2019
Omitting Wu et al., 2023
Omitting Rule et al., 2014.1
Omitting Held et al., 2021
Omitting Elegbede et al., 2020.1
Omitting Yokouchi et al., 2015
Omitting Atci et al., 2021
Omitting Guiliani et al., 2010
Omitting Koh et al., 2019
Omitting Suzuki et al. , 2018
Omitting Ka..smann et al., 2017
Omitting Rosenstein et al., 1992
Omitting Bettington et al., 2013
Omitting Wolfson et al., 2001
Omitting Kim et al., 2019
Omitting Ng et al., 2007
Omitting Cai et al., 2014
Omitting Liengswangwong et al., 1995
Omitting Hu et al., 2020
Omitting Sas−Korczyn..ska et al., 2018
Omitting Soon et al., 2018
Omitting Bang et al. , 2018
Omitting Chen et al., 2016
Omitting van der Pol et al., 1997
Omitting Kasmann et al., 2017
Omitting J Li et al., 2021
Omitting Dawe et al., 2023
Omitting Naidoo et al. , 2018
Omitting Sakin et al., 2019
Omitting Alexopoulos et al., 1997
Omitting Longo et al., 2022
Omitting Lin et al., 2020
Omitting Matutino et al., 2017
Omitting Kang et al., 2022
Omitting Go et al., 2014
Omitting Ayala de Miguel et al., 2020
Omitting Ma et al., 2020
−0.6
−0.4
−0.2
Effect Size (Random−Effects Model)

Figure S4. Forest plots for leave-out-one analysis sorted from low to high effect size for the primary outcome of overall survival. The plot shows the recalculated pooled effects, with one study omitted each time. The dashed line and shaded area represent the estimated pooled effect size and the 95% confidence interval of the original meta-analysis, respectively. No substantial changes in heterogeneity or effect size were observed.

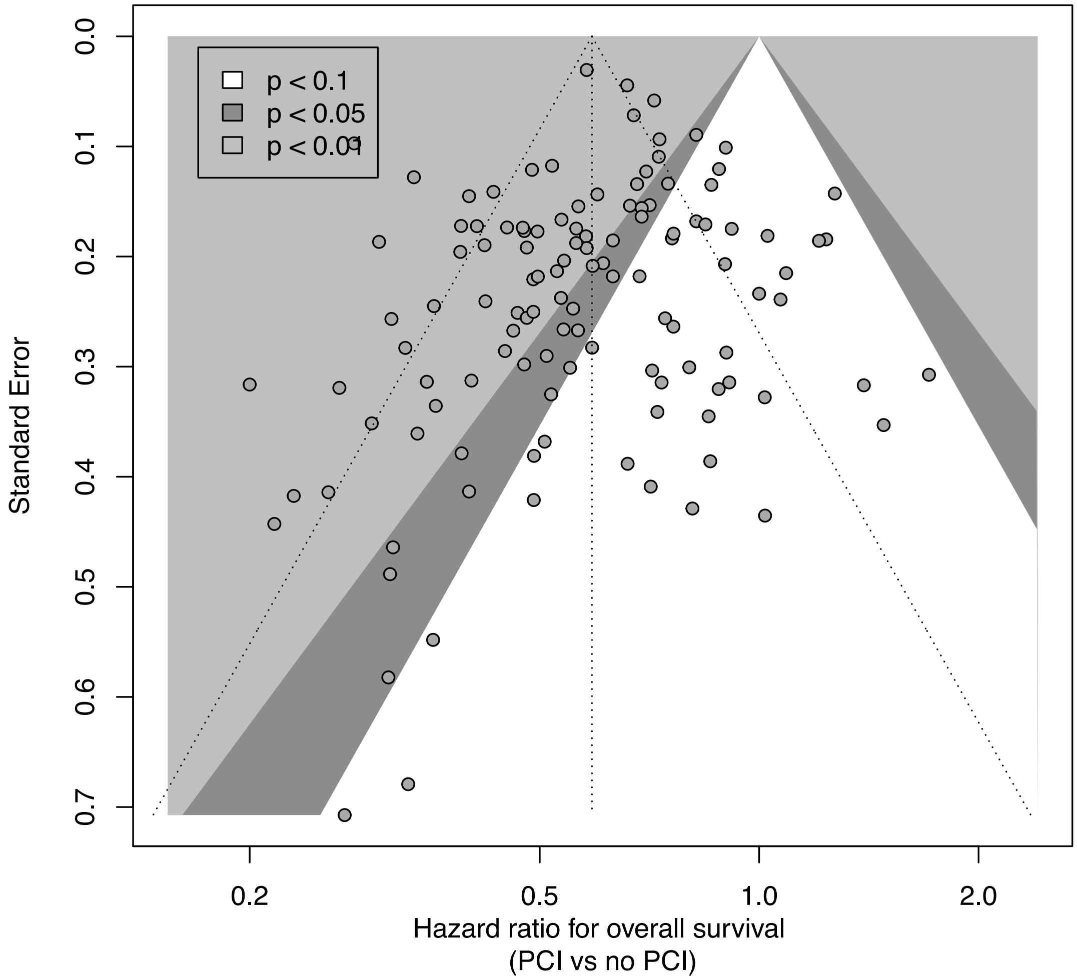

Figure S5. Funnel plot for publication bias among studies comparing PCI with no PCI and reporting on overall survival.

PCI = prophylactic cranial irradiation; vs = versus

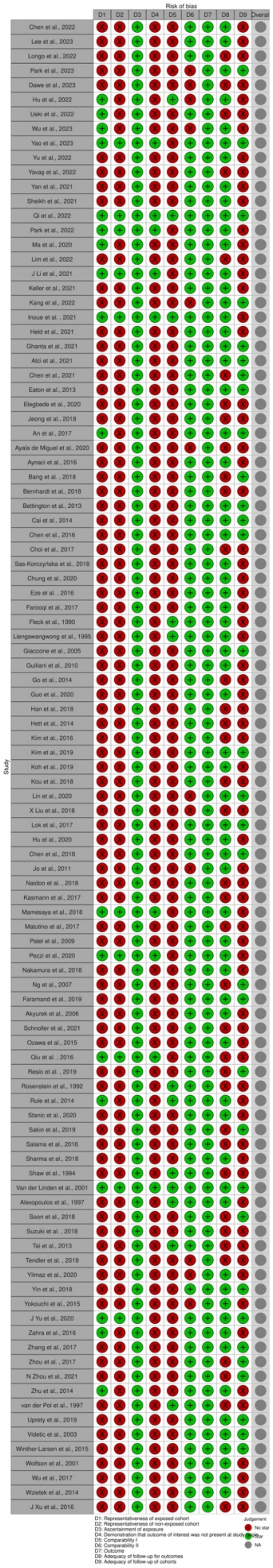
Risk of bias
D1
D2
D3
D4
D5
D6
D7
D8
D9
Overall
Study
Chen et al., 2022
Lee et al., 2023
Longo et al., 2022
Park et al., 2023
Dawe et al., 2023
Hu et al., 2022
Ueki et al., 2022
Wu et al., 2023
Yao et al., 2023
Yu et al., 2022
Yavaş et al., 2022
Yan et al., 2021
Sheikh et al., 2021
Qi et al., 2022
Park et al., 2022
Ma et al., 2020
Lim et al., 2022
J Li et al., 2021
Keller et al., 2021
Kang et al., 2022
Inoue et al. , 2021
Held et al., 2021
Ghanta et al., 2021
Atci et al., 2021
Chen et al., 2021
Eaton et al., 2013
Elegbede et al., 2020
Jeong et al., 2018
An et al., 2017
Ayala de Miguel et al., 2020
Aynaci et al., 2016
Bang et al. , 2018
Bernhardt et al., 2018
Bettington et al., 2013
Cai et al., 2014
Chen et al., 2016
Choi et al., 2017
Sas-Korczyńska et al., 2018
Chung et al., 2020
Eze et al. , 2016
Farooqi et al., 2017
Fleck et al., 1990
Liengswangwong et al., 1995
Giaccone et al., 2005
Guiliani et al., 2010
Go et al., 2014
Guo et al., 2020
Han et al., 2018
Hett et al., 2014
Kim et al., 2016
Kim et al., 2019
Koh et al., 2019
Kou et al., 2018
Lin et al., 2020
X Liu et al., 2018
Lok et al., 2017
Hu et al., 2020
Chen et al., 2018
Jo et al., 2011
Naidoo et al. , 2018
Kasmann et al., 2017
Mamesaya et al., 2018
Matutino et al., 2017
Patel et al., 2009
Pezzi et al., 2020
Nakamura et al., 2018
Ng et al., 2007
Faramand et al., 2019
Akyurek et al., 2006
Schnoller et al., 2021
Ozawa et al., 2015
Qiu et al. , 2016
Resio et al. , 2019
Rosenstein et al., 1992
Rule et al., 2014
Stanic et al., 2020
Sakin et al., 2019
Salama et al., 2016
Sharma et al., 2018
Shaw et al., 1994
Van der Linden et al., 2001
Alexopoulos et al., 1997
Soon et al., 2018
Suzuki et al. , 2018
Tai et al., 2013
Tendler et al. , 2019
Yilmaz et al., 2020
Yin et al., 2018
Yokouchi et al., 2015
J Yu et al., 2020
Zahra et al., 2016
Zhang et al., 2017
Zhou et al. , 2017
N Zhou et al., 2021
Zhu et al., 2014
van der Pol et al., 1997
Uprety et al., 2019
Videtic et al., 2003
Winther-Larsen et al., 2015
Wolfson et al., 2001
Wu et al., 2017
Wzietek et al., 2014
J Xu et al., 2016
D1: Representativeness of exposed cohort
D2: Representativeness of non-exposed cohort
D3: Ascertainment of exposure
D4: Demonstration that outcome of interest was not present at study begin
D5: Comparability I
D6: Comparability II
D7: Outcome
D8: Adequacy of follow-up for outcomes
D9: Adequacy of follow-up of cohorts
Judgement
No star
Star
NA

Figure S6. Newcastle Ottawa Scale plot of overall study quality assessment for non-randomised studies for the primary outcome of overall survival.

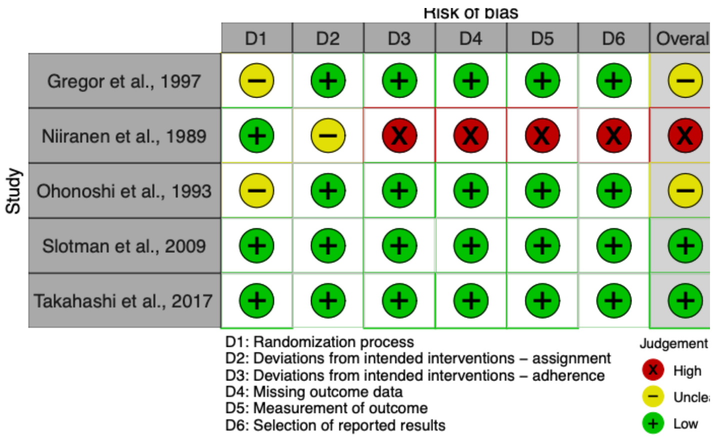

Figure S7. Risk of bias summary plot of randomised studies comparing PCI with no PCI for the primary outcome of overall survival.

Narrative synthesis

Ninety-five studies further reported on survival outcomes in univariable analysis using formats not amenable to meta-analysis (e.g., median survival, annual survival rates; Appendix 2, Table S3). The majority of studies found PCI associated with prolonged survival (Table S4). Out of the studies that radiographically confirmed absence of brain metastases in patients in the whole study cohort (n=5), four found no difference in survival and one did not report statistical significance. Thirty-six of the 105 studies reporting on PCI in multivariable analysis did not report adjusted hazard ratios and were, therefore, not eligible to for meta-analysis (Table S4). None of these studies explicitly reported that absence of brain metastases was confirmed after first-line therapy in patients who did and did not receive PCI therapy.

Table S4. Summary of significant findings of overall survival in studies reporting in formats not amenable to meta-analysis.

Association of PCI with survival	All studies	Studies reporting on limited stage disease patients only	Studies reporting on extensive stage disease patients only
**Univariable analysis**			
Significant improvement with PCI, n	48	30	7
No significant difference between cohorts, n	32	21	2
Varying results for different subgroups*, n	4	NA	NA
**Multivariable analysis**			
PCI identified as prognostic factor, n	19	9	3
PCI not significant, n	14	11	2

*Chen et al., 2014: limited stage disease patients only: not significant, extensive stage disease patients only: significant improvement with PCI; Harden et al., 2011: limited stage disease patients only: not significant, extensive stage disease patients only: significant improvement with PCI; Prelaj et al., 2017, overall cohort: significant improvement with PCI, extensive stage disease patients only: not significant; Rosenstein et al., 1992, patients with complete response only: p=0·05, complete response with disease control in the thorax: p<0·05)

Secondary outcome: incidence of intracranial metastatic disease

Subgroup and heterogeneity analysis

Results from pre-specified subgroup analyses are displayed in Table S5. Between-study heterogeneity was moderate (I^2=58·6, 95% CI, 45·4%-68·6%). Subgroup analysis did not reveal sources of heterogeneity. Meta regression showed that total publication year (p=0.02) and median age of study participants (p=0·04) but not sample size (p=0·16) contributed to between-study heterogeneity, although residual between-study heterogeneity remained moderate (I^2=53·4% and I^2=57·9% for publication year and median age, respectively). No individual studies were identified that disproportionately contributed to overall between-study heterogeneity (Figure S8). Egger's test suggests funnel plot asymmetry (intercept -1·352; 95% CI, -2·06- -9·64; t=-3·717; p<0·001, Figure S9).

Table S5. Subgroup analysis of incidence of intracranial metastatic disease.

Study characteristic	All included studies	Limited stage disease patients only	Extensive stage disease patients only
**Study design**			
RCT	0·44 (95% CI 0·26-0·72), n=7 studies	0·21 (95% CI 0·04-0·97), n=3 studies	0·54 (95% CI 0·22-1·29), n=2 studies
RCS	0·46 (95% CI 0·39-0·54), n=54 studies	0·47 (9% CI 0·39-0·57), n=35 studies	0·50 (95% CI 0·30-0·84), n= 9 studies
PCS	0·35 (95% CI 0·19-0·67), n=3 studies	0·35 (95% CI 0·19-0·67), n=3 studies	NA
**Treatment response to first-line therapy**			
CR	0·39 (95% CI 0·29-0·52), n=9 studies	0·36 (9% CI 0·21-0·60), n=3 studies	NA
CR/PR	0·46 (95% CI 0·33-0·64), n=14 studies	0·42 (95% CI 0·25-0·71), n=9 studies	0·53 (95% CI 0·32-0·87), n=5 studies
Any response (CR + CR/PR)	0·44 (95% CI 0·35-0·55), n=23 studies	0·39 (95% CI 0·28-0·56), n=15 studies	0·53 (95% CI 0·32-0·87), n=5 studies
CR/PR/SD	0·40 (95% CI 0·27-0·60), n=8 studies	0·43 (95% CU 0·22-0·83), n=5 studies	0·33 (95% CI 0·14-0·89), n=3 studies
CR/PR/SD/PD	0·31 (95% CI 0·11-0·87), n=1 study	0·31 (95% CI 0·11-0·86), n=1 study	NA
NR	0·49 (95% CI 0·38-0·61), n=32 studies	0·51 (95% CI 0·39-0·67), n=20 studies	0·89 (9% CI 0·32-2·45), n=3 studies
**Use of brain baseline brain CT/MRI**			
Yes	0·42 (95% CI 0·34-0·51), n=30 studies	0·38 (9% CI 0·32-0·47), n=21 studies	0·61 (95% CI 0·29-1·29), n=5 studies
No	0·44 (95% CI 0·21-0·92) n=4 studies	0·34 (9% CI 0·12-0·93), n=1 study	0·41 (95% CI 0·10-1·74), n=1 study
Not in all patients	0·39 (95% CI 0·19-0·77), n=6 studies	0·41 (9% CI 0·09-1·80), n=4 studies	0·37 (95% CI 0·13-1·06), n=2 studies
NR	0·53 (95% CI 0·40-0·70), n=24 studies	0·59 (9% CI 0·41-0·83), n=15 studies	0·54 (95% CI 0·20-1·52), n=3 studies
**MRI confirmation of no IMD at restaging**			
Yes	0·51 (95% CI 0·26-0·99), n=4 studies	0·44 (95% CI 0·15-1·29), n=3 studies	0·69 (5% CI 0·34-1·39), n=1 study
No	0·39 (95% CI 0·29-0·52), n=18 studies	0·35 (95% CI 0·23-0·55), n=12 studies	0·36 (95% CI 0·14-0·92), n=2 studies
NR	0·48 (95% CI 0·40-0·58), n=42 studies)	0·51 (9% CI 0·41-0·63), n=26 studies	0·54 (95% CI 0·31-0·96), n=8 studies
**Use of platinum-based therapy**			
Yes	0·40 (9% CI 0·33-0·49), n=24 studies	0·37 (9% CI 0·30-0·45), n=15 studies	0·50 (95% CI 0·30-0·83), n=8 studies
No	0·32 (95% CI 0·22-0·48), n=9 studies	0·32 (9% CI 0·20-0·53), n=6 studies	NA
Not administered to all patients	0·49 (95% CI 0·28-0·84), n=10 studies	0·51 (9% CI 0·28-0·91), n=9 studies	NA
NR	0·58 (95% CI 0·45-0·76), n=21 studies	0·63 (95% CI 0·41-0·90), n=11 studies	0·55 (95% CI 0·22-1·36), n=3 studies
**AHRQ***			
Good	0·45 (95% CI 0·24-0·85), n=4 studies	0·44 (9% CI 0·14-1·42), n=3 studies	NA
Fair	0·27 (95% CI 0·09-0·88), n=3 studies	0·31 (9% CI 0·00-43·08), n=2 studies	0·20 (95% CI 0·03-1·20), n=1 study
Poor	0·47 (9% CI 0·40-0·56), n=51	0·47 99% CI 0·39-0·58), n=33 studies	0·55 (95% CI 0·32-0·93), n=8 studies
**RoB****			
Low	0·54 (95% CI 0·02-15·12), n=2 studies	NA	0·54 (95% CI 0·22-1·28), n=2 studies
Some concerns	0·34 (95% CI 0·03-3·80), n=2 studies	0·29 (9% CI 0·09-0·89), n=1 study	NA
High	0·10 (95% CI 0·00-2·80), n=2 studies	0·10 (9% CI 0·0=2·80), n=2 studies	NA

AHRQ = Agency for Health Research and Quality; CR = complete response; CT = computed tomography; MRI = magnetic resonance imaging; NA=not applicable; NR = not reported; nRCT = non-randomised controlled trial; PCS = prospective cohort study; PR = partial response; SD = stable disease; RCS = retrospective cohort study; RCT = randomised controlled trial; RoB = risk of bias

*AHRQ only reported for non-randomised trials

**RoB only reported for randomised controlled trials

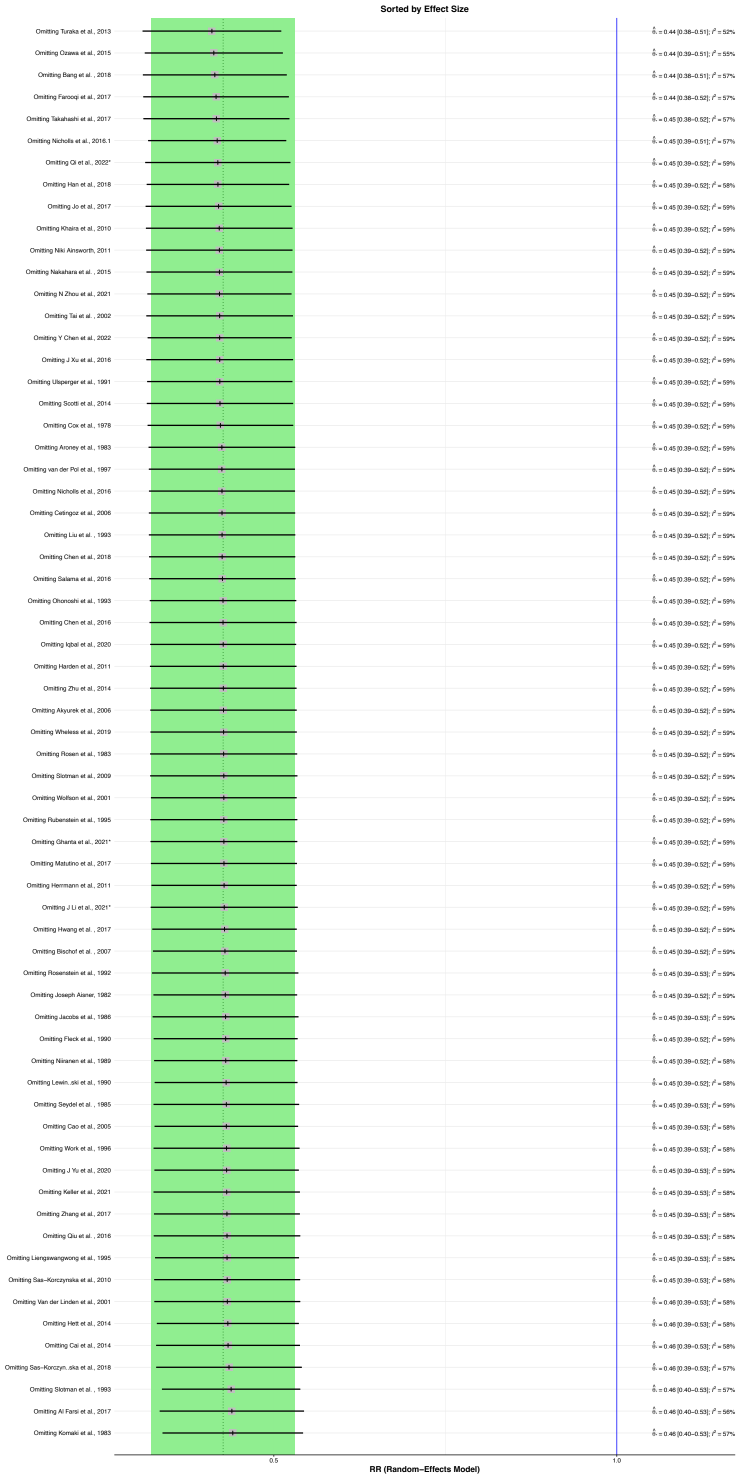

Figure S8. Forest plots for leave-out-one analysis sorted from low to high effect size for the secondary outcome of incidence of brain metastases. The plot shows the recalculated pooled effects, with one study omitted each time. The dashed line and shaded area represent the estimated pooled effect size and the 95% confidence interval of the original meta-analysis, respectively. No substantial changes in heterogeneity or effect size were observed.

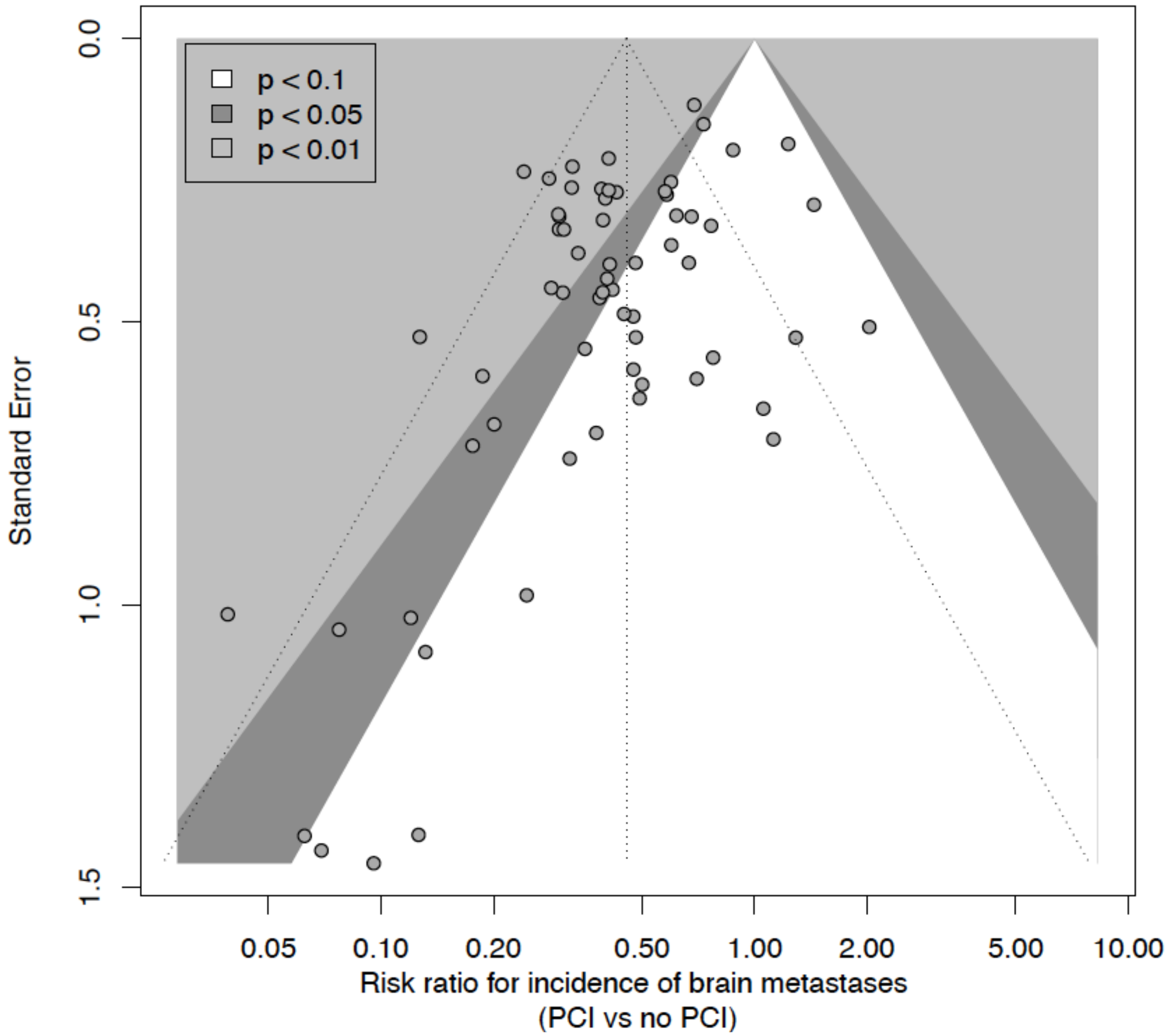

Figure S9. Funnel plot for publication bias among studies comparing PCI with no PCI and reporting on incidence of brain metastases.
PCI = prophylactic cranial irradiation; vs = versus

Narrative synthesis

All studies included in the primary analysis of incidence of brain metastases reported unadjusted risk ratios. Fifteen studies also reported on brain metastases incidence and/or time to brain metastases with or without censoring, but due to inconsistent reporting formats and limited number of studies, these were not included in the formal meta-analysis (Appendix 2, Table S6).

Further, 36 studies reported on brain metastases incidence in formats not amenable to meta-analysis, of which 21 found statistically significant reduction in the incidence of brain metastases in patients who received PCI compared to patients who did not, while 11 reported no difference between treatment cohort. Six out of the 36 studies radiographically ensured absence of brain metastases at restaging, of which 3 found improvement in terms of brain metastases incidence while the other 3 did not (Appendix 2, Table S6).

Secondary outcome: intracranial progression-free survival

Twenty studies reported on intracranial progression-free survival and found superior intracranial progression-free survival in patients who received PCI compared to those who did not (HR 0·37; 95%CI, 0·29-0·48; p<0·001; n=3347 patients; Figure S10).

Author	Study design	No. patients (PCI)	No. patients (no PCI)	AHRQ/RoB	HR	95% CI	Weight
Disease stage = ED							
Keller et al., 2020	RCS	137	87	Fair	0.18	[0.10; 0.33]	5.7%
Bang et al. , 2018	RCS	68	87	Poor	0.36	[0.21; 0.61]	6.0%
Chen et al., 2016	RCS	45	159	Poor	0.38	[0.20; 0.72]	5.4%
Chung et al., 2020	RCS	53	137	Poor	0.55	[0.28; 1.11]	5.0%
Random effects model					0.34	[0.16; 0.69]	22.1%
Prediction interval						[0.06; 1.88]	--
Heterogeneity: I^2 = 54%, τ = 0.330, χ^2_3 = 6.47 (p = 0.091)							
Disease stage = LD							
Van der Linden et al., 2001	RCS	65	37	Good	0.14	[0.06; 0.31]	4.4%
Sas−Korczynska et al., 2010	RCS	86	43	Poor	0.15	[0.08; 0.29]	5.3%
Liengswangwong et al., 1995	RCS	24	19	Fair	0.21	[0.07; 0.68]	2.9%
J Li et al., 2021	RCS	77	113	Good	0.23	[0.13; 0.43]	5.6%
van der Pol et al., 1997	PCS	18	21	Poor	0.27	[0.10; 0.75]	3.4%
Sas−Korczyn..ska et al., 2018	RCS	167	104	Poor	0.27	[0.17; 0.45]	6.2%
Guiliani et al., 2010	RCS	127	80	Poor	0.29	[0.16; 0.53]	5.6%
Zahra et al., 2016	RCS	16	22	Fair	0.35	[0.07; 1.67]	1.9%
Eze et al. , 2016	RCS	71	113	Poor	0.42	[0.26; 0.69]	6.2%
Chen et al., 2018	RCS	19	33	Poor	0.49	[0.18; 1.37]	3.4%
Inoue et al. , 2021*	RCS	32	32	Good	0.51	[0.23; 1.14]	4.4%
Koh et al., 2019	RCS	160	190	Poor	0.54	[0.38; 0.76]	7.1%
Kim et al., 2019	RCS	139	95	Poor	0.62	[0.37; 1.04]	6.1%
Gregor et al., 1997	RCT	194	120	Some concerns	0.75	[0.58; 0.96]	7.6%
N Zhou et al., 2021	RCS	43	121	Poor	0.76	[0.21; 2.79]	2.5%
Zhu et al., 2014	RCS	67	126	Fair	0.83	[0.45; 1.54]	5.4%
Random effects model					0.38	[0.28; 0.52]	77.9%
Prediction interval						[0.13; 1.09]	--
Heterogeneity: I^2 = 73%, τ = 0.468, χ^2_{15} = 55.2 (p < 0.001)							
**Random effects model**					**0.37**	**[0.29; 0.48]**	**100.0%**
Heterogeneity: I^2 = 71%, τ = 0.435, χ^2_{19} = 66.00 (p < 0.001)							

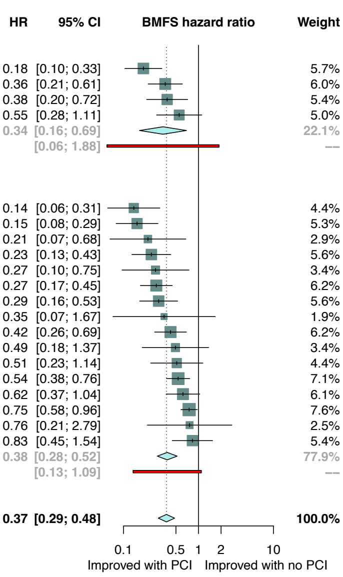

Figure S10. Random-effects meta-analysis of PCI versus no PCI for secondary outcome of intracranial progression-free survival.

Subgroup and heterogeneity analysis

Pre-specified subgroup analyses were performed only for the overall study cohort due to limited sample size when considering studies according to participant disease stage (Table S7).

Between-study heterogeneity for analysis of intracranial progression-free survival was moderate (I^2=71·2%; 95% CI, 54·8-81·7%). This was reduced when considering cohorts exclusively consisting of studies with poor Agency for Health Research and Quality rating (I^2=45·6%) but was not affected by other subgroup analyses. Residual between-study heterogeneity was not reduced in meta regression analysis by publication year, median age of study participants, or study sample size. No individual study was identified that contributed significantly to between-study heterogeneity (Figure S11). Egger's test suggests funnel plot asymmetry (intercept 1·983; 95% CI, 0·66-3·3, t=2·944; p=0·008; Figure S12).

Table S7. Subgroup analysis of intracranial progression-free survival

Study characteristic	All included studies
**Study design**	
RCT	0·75 (95% CI 0·33-1·71), n=1 study
RCS	0·36 (95% CI 0·27-0·46), n=18 studies
PCS	0·27 (95% CI 0·07-0·97), n= 1 study
**Treatment response to first-line therapy**	
CR	0·35 (95% CI 0·09-1·28), n=4 studies
CR/PR	0·33 (95% CI 0·22-0·49), n=7 studies
Any response (CR + CR/PR)	0·33 (95% CI 0·23-0·47), n=11 studies
CR/PR/SD	0·23 (95% CI 0·01-4·57), n=2 studies
CR/PR/SD/PD	0·48 (95% CI 0·09-2·55), n=2 studies
NR	0·63 (95% CI 0·44-0·91), n=5 studies
**Use of brain baseline brain CT/MRI**	
Yes	0·40 (95% CI 0·29-0·57), n=11 studies
NR, only in a subset of patients	0·33 (95% CI 0·21-0·53), n=9 studies
**MRI confirmation of no brain metastases at restaging**	
Yes	0·33 (95% CI 0·00-45·38), n=2 studies
No	0·36 (95% CI 0·16-0·81), n=5 studies
NR	0·38 (9% CI 0·28-0·53), n=13 studies
**Use of platinum-based therapy**	
Yes	0·38 (95% CI 0·29-0·51), n=13 studies
No	0·18 (95% CI 0·00-11·97), n=2 studies
Not administered to all patients	0·54 (95% CI 0·28-1·06), n=4 studies
NR	0·18 (95% CI 0·08-0·45), n=1 study
**AHRQ***	
Good	0·25 (95% CI 0·05-1·22), n=3 studies
Fair	0·34 (95% CI 0·10-1·17), n=4 studies
Poor	0·38 (95% CI 0·29-0·50), n=12 studies
**RoB****	
Some concerns	0·75 (95% CI 0·33-1·70), n=1 study

AHRQ = Agency for Health Research and Quality; CR = complete response; CT = computed tomography; MRI = magnetic resonance imaging; NA=not applicable; NR = not reported; nRCT = non-randomised controlled trial; PCS = prospective cohort study; PR = partial response; SD = stable disease; RCS = retrospective cohort study; RCT = randomised controlled trial; RoB = risk of bias

*AHRQ only reported for non-randomised trials

**RoB only reported for randomised controlled trials

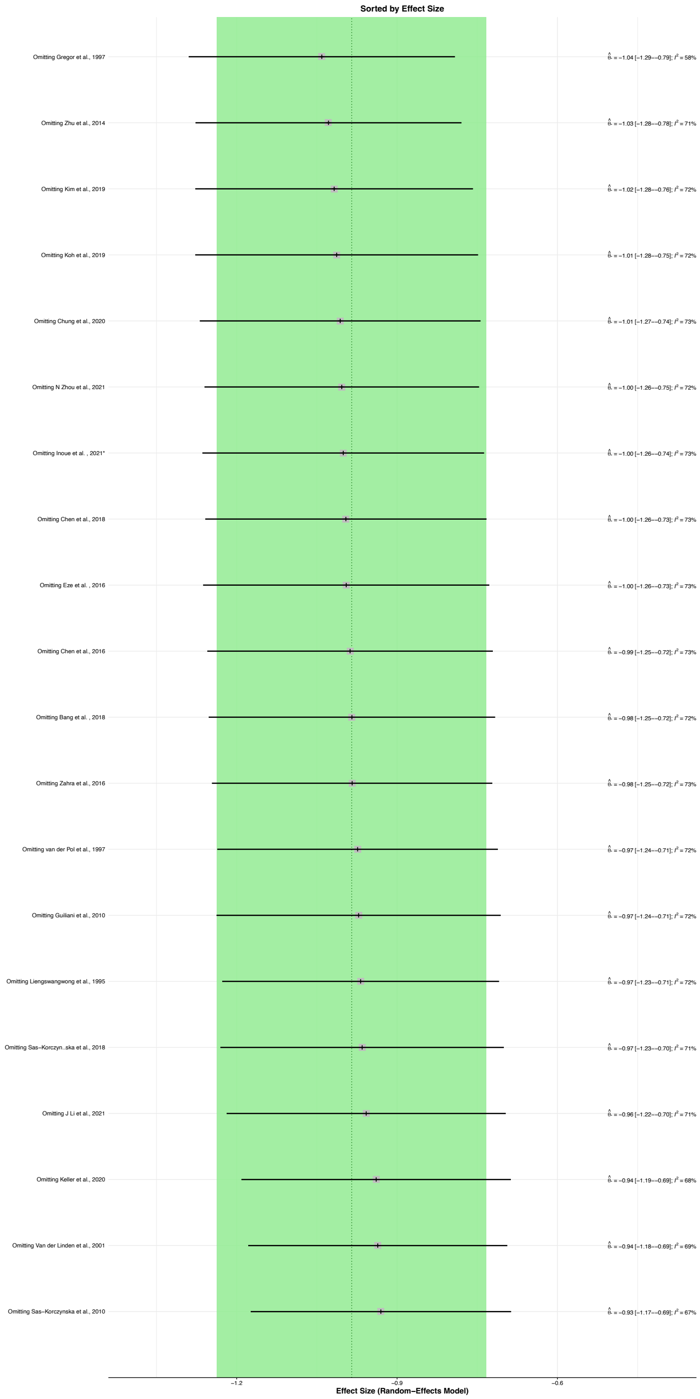

Figure S11. Forest plots for leave-out-one analysis sorted from low to high effect size for the secondary outcome of intracranial progression-free survival. The plot shows the recalculated pooled effects, with one study omitted each time. The dashed line and shaded area represent the estimated pooled effect size and the 95% confidence interval of the original meta-analysis, respectively. No substantial changes in heterogeneity or effect size were observed.

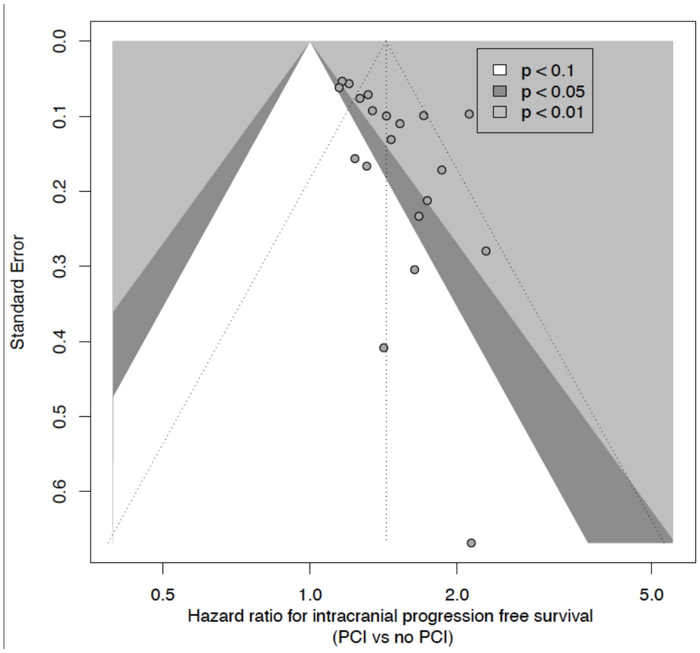

Figure S12. Funnel plot for publication bias among studies comparing PCI with no PCI and reporting on intracranial progression free survival.
PCI = prophylactic cranial irradiation; vs = versus

Narrative synthesis

Forty-two studies reported on time to brain metastases in univariable analysis in formats not amenable to statistical pooling, with 22 studies reporting improvement in patients who received PCI and eight finding no difference. Six out of these 42 studies confirmed absence of brain metastases after first-line therapy: four found superior intracranial progression survival in cohorts who received PCI and one found no difference (Appendix 2, Table S6).

Secondary outcome: progression-free survival

Progression-free survival was in favour of receipt of PCI when considering all available studies in meta-analysis (HR 0·58; 95% CI, 0·50-0·767; p=<0·001; n=19 studies; n=2,224 patients; Figure S13).

Subgroup and heterogeneity analysis

The overall between-study heterogeneity was 49·8% (95% CI, 15·8-73·9), which was reduced in studies reporting on cohorts of patients who received brain imaging at baseline using either computed tomography or magnetic resonance imaging (I^2=31·2%), retrospective cohort studies (I^2=16·6%), and those with poor quality according to the Agency for Health Research and Quality rating (I^2=1·1%, Table S9). Meta regression according to publication year, median age of study participants, or sample size did not significantly reduce residual heterogeneity. Leave-out-one analysis identified that the study by Takayashi et al. contributed significantly to between-study heterogeneity (I^2=17·0%, Figure S14). Overall effect size when omitting this trial from the meta-analysis remained in favour of PCI therapy (HR 0·58; 95% CI, 0·45-0·71; n=18 studies; n=2,000 patients; p<0·001). Egger's test did not indicate presence of funnel plot asymmetry (intercept -1·519; 95% CI, -3·07- -0·030, t=-1·92 p=0·0708; Figure S15).

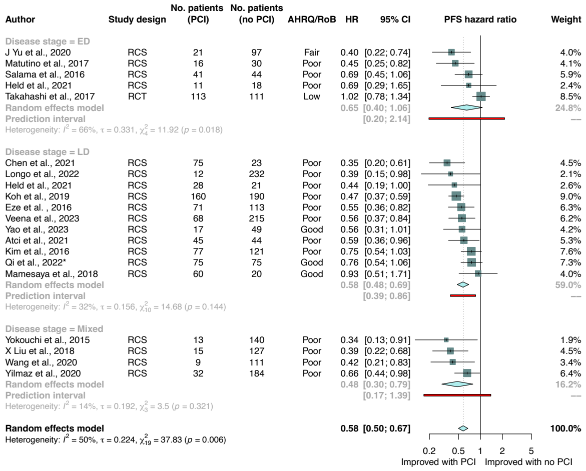

Figure S13. Random-effects meta-analysis of PCI versus no PCI for secondary outcome of progression-free survival.

Table S9. Subgroup analysis of progression-free survival.

Study characteristic	All included studies
**Study design**	
RCT	1·02 (95% CI 0·68-1·54), n=1 study
RCS	0·56 (95% CI 0·48-0·65), n=19 studies
**Treatment response to first-line therapy**	
CR/PR	0·62 (95% CI 0·37-1·06), n=6 studies
CR/PR/SD	0·53 (95% CI 0·35-0·82), n=5 studies
CR/PR/SD/PD	0·68 (95% CI 0·16-2·94), n=2 studies
NR	0·52 (95% CI 0·36-0·75), n=7 studies
**Use of brain baseline brain CT/MRI**	
Yes	0·57 (95% CI 0·45-0·75), n=10 studies
NR	0·58 (95% CI 0·44-0·78), n=10 studies
**MRI confirmation of no brain metastases at restaging**	
Yes	0·85 (95% CI 0·69-1·24), n=4 studies
No	0·49 (95% CI 0·24-1·04), n=3 studies
NR	0·53 (95% CI 0·45-0·62), n=13 studies
**Use of platinum-based therapy**	
Yes	0·63 (95% CI 0·51-0·77), n=14 studies
Not administered to all patients	0·47 (95% CI 0·27-0·79), n=1 study
NR	0·50 (95% CI 0·23-1·06), n=5 studies
**AHRQ***	
Good	0·74 (95% CI 0·44-1·24), n=3 studies
Fair	0·40 (95% CI 0·21-0·77), n=1 study
Poor	0·54 (95% CI 0·46-0·62), n=15 studies
**RoB****	
Low	1·02 (95% CI 0·72-1·46), n=1 study

AHRQ = Agency for Health Research and Quality; CR = complete response; CT = computed tomography; MRI = magnetic resonance imaging; NR = not reported; PR = partial response; SD = stable disease; RCS = retrospective cohort study; RCT = randomised controlled trial; RoB = risk of bias

*AHRQ only reported for non-randomised trials

**RoB only reported for randomised controlled trials

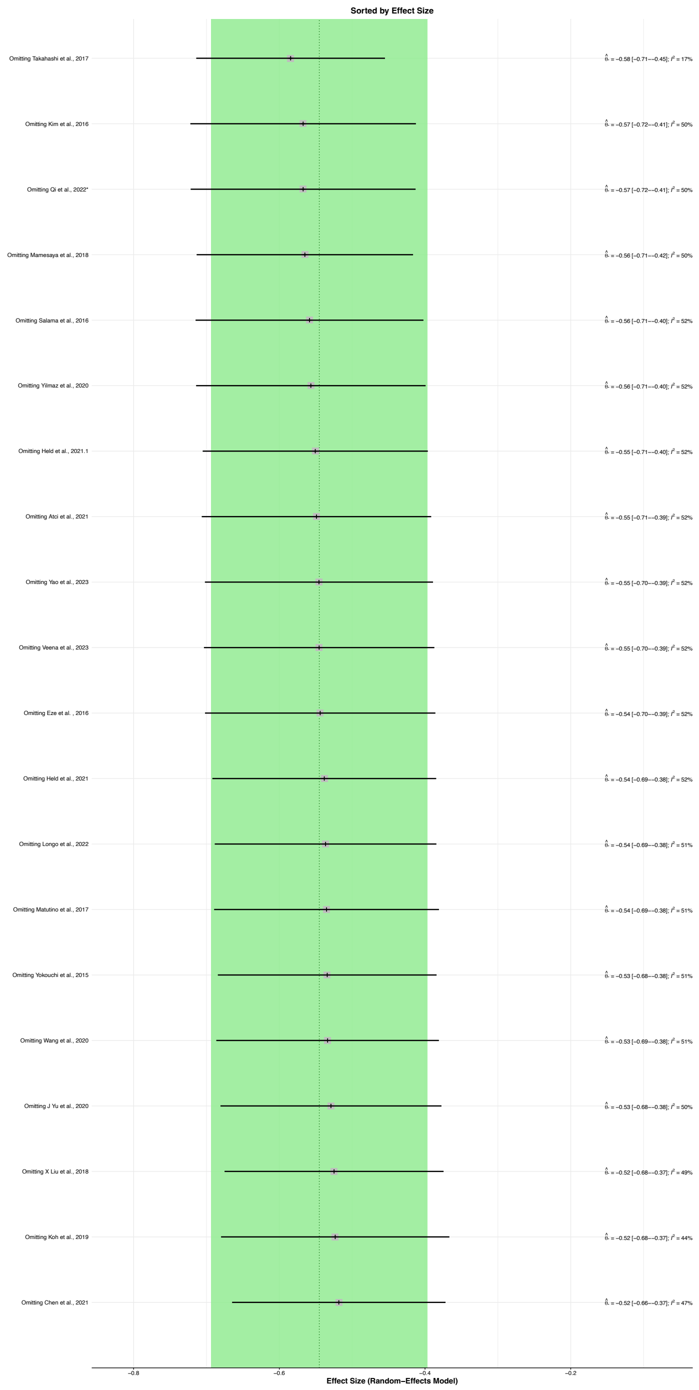

Figure S14. Forest plots for leave-out-one analysis sorted from low to high effect size for the secondary outcome of progression-free survival. The plot shows the recalculated pooled effects, with one study omitted each time. The dashed line and shaded area represent the estimated pooled effect size and the 95% confidence interval of the original meta-analysis, respectively. No substantial changes in heterogeneity or effect size were observed.

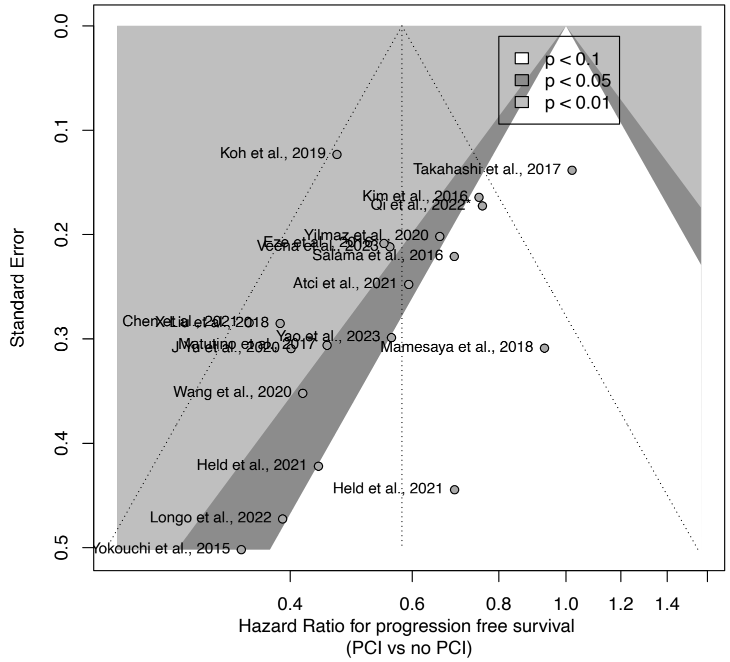

Figure S15. Funnel plot for publication bias among studies comparing PCI with no PCI and reporting on progression free survival.

PCI = prophylactic cranial irradiation; vs = versus

Narrative synthesis

Forty-one studies reported on progression-free survival in univariable and/or multivariable analysis but were not eligible for meta-analysis. Seventeen studies found statistically significant improvement in progression-free survival in cohorts of patients who received PCI compared with those who did not, and 10 found no difference. Three of these radiographically confirmed absence of brain metastases in patients who did and did not receive PCI therapy and all found no difference in progression free survival with the addition with PCI therapy. Information on studies reporting on multivariable analysis can be found in the supplementary materials (Appendix 2, Table S8).

Secondary outcome: disease-free survival

Seven studies[14,16,56,66,156,178,206] reported on disease-free survival in formats amenable to multivariable analysis (HR 0·57; 95% CI, 0·36-0·89; p=0·023; n=866 patients; Figure S16).

Subgroup and heterogeneity analysis

Subgroup analysis according to pre-specified subgroups was performed, but no reduction in the overall between-study heterogeneity (I^2=65·3%; 95% CI, 22·0-84·5) was found (Table S11). Publication year, sample size, and median age of study participants did not contribute significantly to between-study heterogeneity in meta regression (p>0·05). Between study heterogeneity was not significantly influenced by any one study (Figure S17). Egger's test did not suggest presence of funnel plot asymmetry (intercept 0·676, 95% CI -2·62- -3·97, t=0·402, p=0·704; Figure S18).

Author	Study design	No. patients (PCI)	No. patients (no PCI)	AHRQ/RoB	HR	95% CI	Weight
**Disease stage = ED**							
Slotman et al., 2009	RCT	143	143	Low	0.76	[0.60; 0.97]	21.2%
**Disease stage = LD**							
Wolfson et al., 2001	PCS	15	12	Poor	0.33	[0.13; 0.82]	9.5%
Van der Linden et al., 2001	RCS	65	37	Good	0.42	[0.26; 0.66]	16.9%
Han et al., 2018	RCS	44	102	Poor	0.95	[0.55; 1.63]	15.4%
Fleck et al., 1990	RCS	38	20	Poor	1.02	[0.54; 1.94]	13.5%
**Random effects model**					**0.62**	**[0.26; 1.51]**	**55.3%**
**Prediction interval**						**[0.07; 5.95]**	--
Heterogeneity: $I^2 = 68\%$, $\tau = 0.445$, $\chi^2_3 = 9.37$ ($p = 0.025$)							
**Disease stage = Mixed**							
Yokouchi et al., 2015	RCS	13	140	Poor	0.34	[0.13; 0.91]	8.6%
Ayala de Miguel et al., 2020	PCS	54	40	Poor	0.35	[0.20; 0.62]	14.9%
**Random effects model**					**0.35**	**[0.30; 0.41]**	**23.5%**
Heterogeneity: $I^2 = 0\%$, $\tau = 0$, $\chi^2_1 = 0$ ($p = 0.960$)							
**Random effects model**					**0.57**	**[0.36; 0.89]**	**100.0%**
Heterogeneity: $I^2 = 65\%$, $\tau = 0.382$, $\chi^2_6 = 17.27$ ($p = 0.008$)							

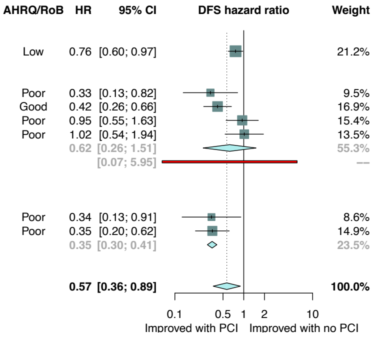

Figure S16. Random-effects meta-analysis of PCI versus no PCI for secondary outcome of intracranial progression-free survival.

Table S11. Subgroup analysis of disease-free survival.

Study characteristic	All included studies
**Study design**	
RCT	0·76 (95% CI 0·37-1·58), n=1 study
RCS	0·64 (95% CI 0·27-1·50), n=4 studies
PCS	0·34 (95% CI 0·23-0·51), n=1 study
**Treatment response to first-line therapy**	
CR	0·53 (95% CI 0·12-0·27), n=3 studies
CR/PR	0·76 (95% CI 0·28-2·07), n=1 study
CR + CR/PR	0·60 (95% CI 0·28-1·30), n=4 studies
NR	0·51 (95% CI 0·11-2·26), n=3 studies
**Use of brain baseline brain CT/MRI**	
Yes	1·02 (95% CI 0·39-2·64), n=1 study
No	0·76 (95% CI 0·36-1·60), n=1 study
NR	0·46 (95% CI 0·26-0·82), n=5 studies
**MRI confirmation of no brain metastases at restaging**	
Yes	0·42 (95% CI 0·15-1·19), n=1 study
No	0·76 (95% CI 0·28-2·01), n=1 study
NR	0·55 (95% CI 0·26-1·15), n=5 studies
**Use of platinum-based therapy**	
Yes	0·34 (95% CI 0·09-1·30), n=1 study
No	0·63 (95% CI 0·00-1·78·81), n=2 studies
NR	0·57 (95% CI 0·25-1·32), n=4 studies
**AHRQ***	
Good	0·42 (95% CI 0·15-1·19), n=1 study
Poor	0·55 (95% CI 0·26-1·15), n=5 studies
**RoB****	
Low	0·76 (95% CI 0·29-2·01), n=1 study

AHRQ = Agency for Health Research and Quality; CR = complete response; CT = computed tomography; MRI = magnetic resonance imaging; NR = not reported; PR = partial response; PCS = prospective cohort study; SD = stable disease; RCS = retrospective cohort study; RCT = randomised controlled trial; RoB = risk of bias
*AHRQ only reported for non-randomised trials
**RoB only reported for randomised controlled trials

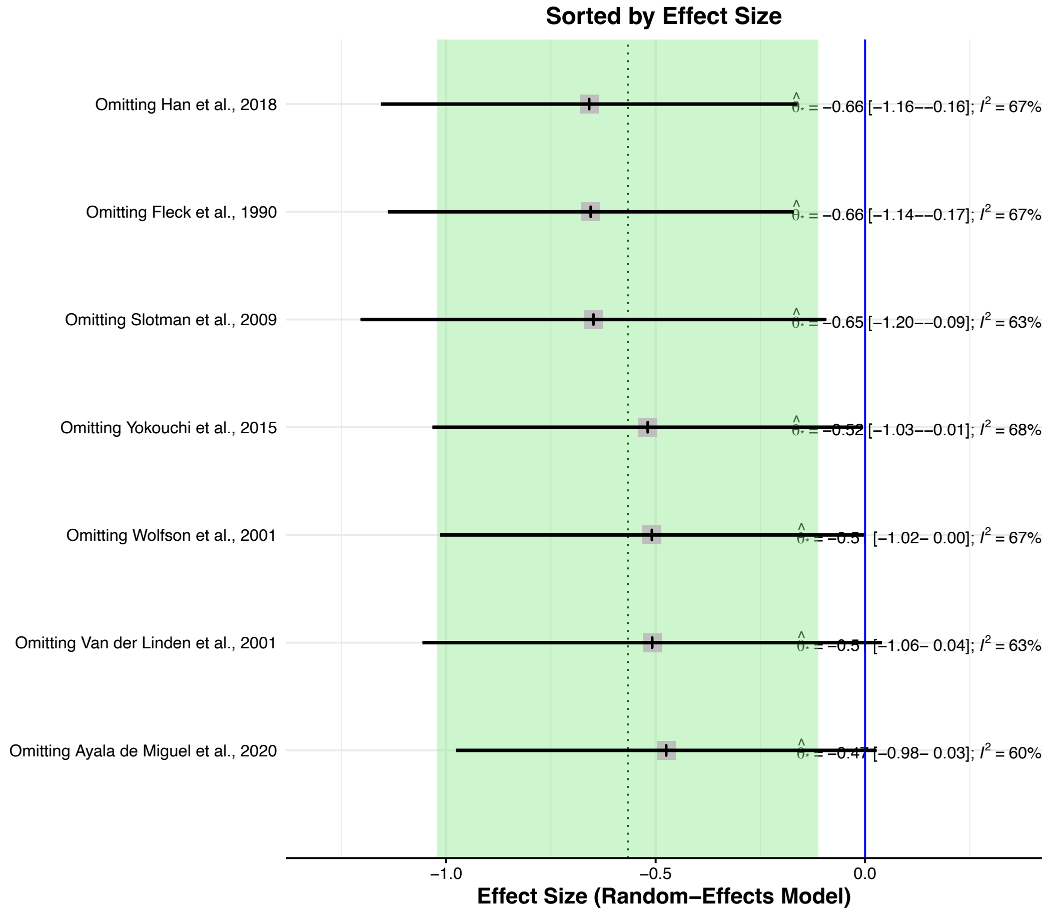

Figure S17. Forest plots for leave-out-one analysis sorted from low to high effect size for the secondary outcome of disease-free survival. The plot shows the recalculated pooled effects, with one study omitted each time. The dashed line and shaded area represent the estimated pooled effect size and the 95% confidence interval of the original meta-analysis, respectively. No substantial changes in heterogeneity or effect size were observed.

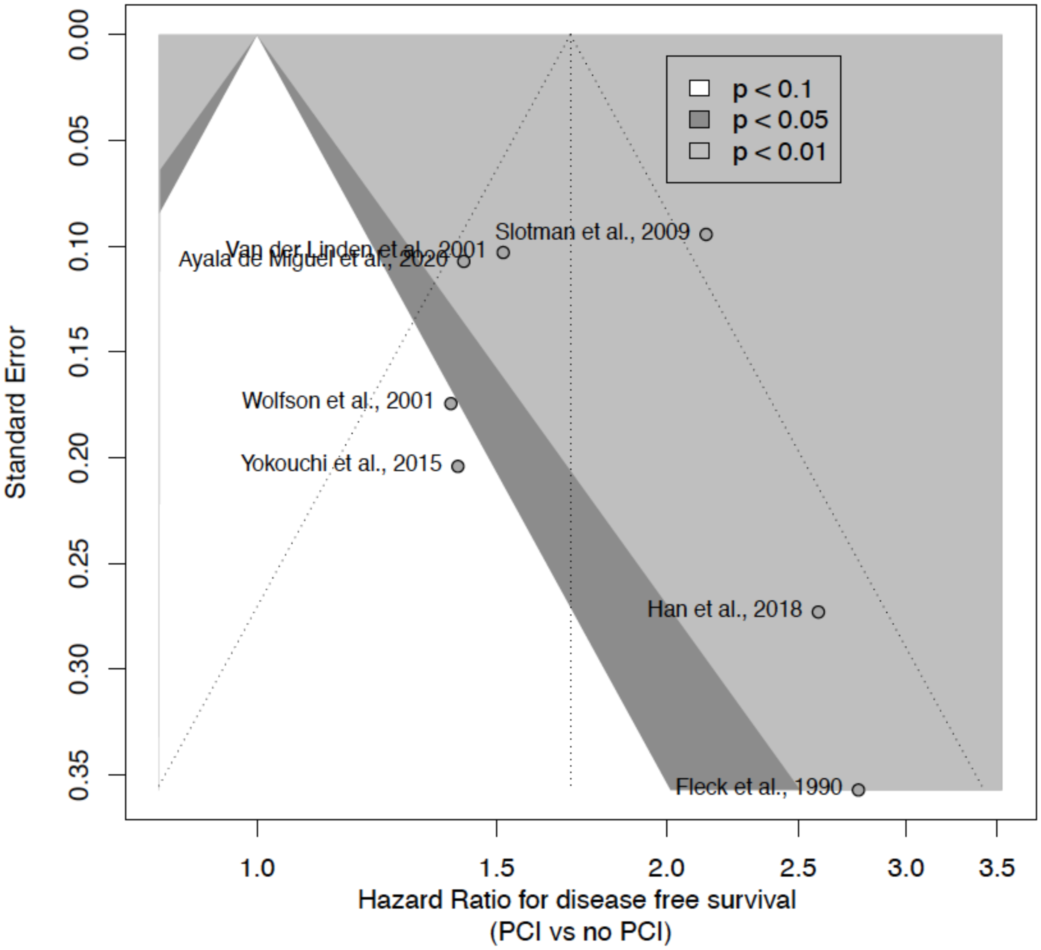

Figure S18. Funnel plot for publication bias among studies comparing PCI with no PCI and reporting on disease free survival.

PCI = prophylactic cranial irradiation; vs = versus

Narrative synthesis

Out of the thirteen studies[14-16,18,51,54,65,88,92,131,156,165,176,183,199,203,206,212,227] that reported on disease-free survival in formats not amenable to meta-analysis, 9 found improvement with administration of PCI therapy[16,51,54,88,92,131,156,165,203] and 2 did not[176,183] while 2 did not report outcomes of statistical analysis[199,227]. None of these studies radiographically confirmed absence of brain metastases at restaging. Ten studies reported on disease-free survival in multivariable analysis with differing endpoint definitions (Appendix 2, Table S10)[14,15,18,51,65,88,92,199,206,212].

Secondary outcome: incidence of brain metastases as first site of recurrence

PCI reduced the indicine of brain metastases as first site of recurrence (RR 0·44; 95% CI, 0·34-0·59; p<0·001; n=18 studies; n=2827 patients; Figure S19).

Subgroup analysis and between-study heterogeneity

Moderate between-study heterogeneity among all studies (I^2=54·1%, 95% CI, 23·9-72·3) was reduced when considering studies reporting on patients who received either computed tomography or magnetic resonance imaging at baseline (I^2=46·5%) or those in which ≥90% of patients received platinum-based chemotherapy (I^2=40·8%) and was unaltered in other pre-specified subgroup analysis (Table S13). Sample size significantly contributed to between-study heterogeneity in meta regression (residual heterogeneity I^2=31.8%). Removing the report by Komaki et al. 2016 from the model also reduced between-study heterogeneity (I^2=46), although the overall effect size remained unchanged (RR 0·42, 95% CI 0·32-0·55, Figure S20). Egger's test indicates presence of funnel plot asymmetry (intercept -1·853, 95% CI -2·72- -1·01, t=-4·271, p<0·0001; Figure S21).

Author	Study design	IMD incidence (PCI)	No. patients (PCI)	IMD incidence (no PCI)	No. patients (no PCI)	AHRQ/RoB	RR	95% CI	Weight
**Disease stage = LD**									
Ng et al., 2007	RCS	3.00	39	11.00	25	Poor	0.17	[0.05; 0.57]	3.5%
Liengswangwong et al., 1995	RCS	2.00	24	9.00	19	Fair	0.18	[0.04; 0.72]	2.7%
Fleck et al., 1990	RCS	0.00	38	1.00	20	Poor	0.18	[0.01; 4.16]	0.6%
J Li et al., 2021*	RCS	6.00	69	30.00	69	Good	0.20	[0.09; 0.45]	5.7%
Tai et al., 2013	RCS	2.00	40	8.00	48	Poor	0.30	[0.07; 1.33]	2.4%
Rosenstein et al., 1992	RCS	5.00	36	12.00	26	Poor	0.30	[0.12; 0.75]	5.0%
Park et al., 2022	PCS	3.00	22	9.00	21	Good	0.32	[0.10; 1.02]	3.6%
Eagan et al., 1981	RCT	2.00	15	6.00	15	Some concerns	0.33	[0.08; 1.39]	2.6%
Choi et al., 2017	RCS	5.05	38	36.63	99	Poor	0.36	[0.15; 0.84]	5.4%
Nakamura et al., 2018	RCS	17.00	93	28.00	69	Poor	0.45	[0.27; 0.75]	8.5%
Tai et al., 2013	RCS	6.00	128	5.00	49	Poor	0.46	[0.15; 1.44]	3.7%
Videtic et al., 2003	RCS	10.00	48	54.00	166	Poor	0.64	[0.35; 1.16]	7.7%
Komaki et al., 2016	RCS	66.00	307	71.00	271	Poor	0.82	[0.61; 1.10]	11.0%
Choi et al., 2017	RCS	17.84	52	37.40	91	Poor	0.83	[0.53; 1.31]	9.3%
van der Pol et al., 1997	PCS	3.00	18	2.00	21	Poor	1.75	[0.33; 9.34]	2.0%
**Random effects model**							**0.45**	**[0.33; 0.63]**	**73.7%**
**Prediction interval**								**[0.19; 1.07]**	**--**
Heterogeneity: $I^2 = 54\%$, $\tau = 0.363$, $\chi^2_{14} = 30.25$ ($p = 0.007$)									
**Disease stage = Mixed**									
Sculier et al., 1987	RCS	18.00	146	20.00	60	Poor	0.37	[0.21; 0.65]	8.0%
Ohonoshi et al., 1993	RCT	2.00	23	5.00	23	Some concerns	0.40	[0.09; 1.86]	2.3%
Arriagada et al., 2002	RCT	49.00	245	68.00	250	Some concerns	0.74	[0.53; 1.01]	10.7%
**Random effects model**							**0.55**	**[0.20; 1.51]**	**21.1%**
**Prediction interval**								**[0.01; 52.71]**	**--**
Heterogeneity: $I^2 = 57\%$, $\tau = 0.271$, $\chi^2_{2} = 4.63$ ($p = 0.099$)									
**Disease stage = NR**									
Lishner et al., 1990	RCS	2.00	48	4.00	10	Poor	0.10	[0.02; 0.49]	2.3%
**Disease stage = ED**									
Matutino et al., 2017	RCS	2.00	16	16.00	30	Poor	0.23	[0.06; 0.89]	2.9%
**Random effects model**							**0.44**	**[0.34; 0.59]**	**100.0%**
Heterogeneity: $I^2 = 54\%$, $\tau = 0.369$, $\chi^2_{19} = 41.43$ ($p = 0.002$)									

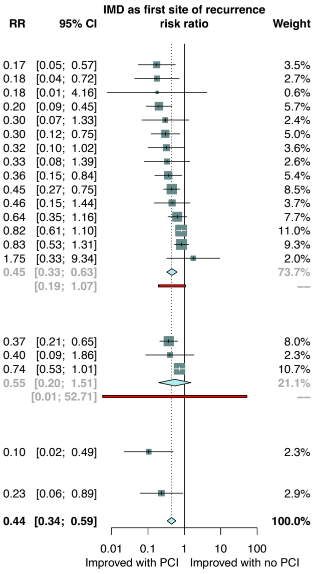

Figure S19. Random-effects meta-analysis of PCI versus no PCI for secondary outcome of incidence of brain metastases as first site of recurrence.

Table S13. Subgroup analysis of incidence of brain metastases as first site of recurrence.

Study characteristic	All included studies
Study design	
RCT	0·58 (95% CI 0·21-1·62), n=3 studies
RCS	0·41 (95% CI 0·29-0·57), n=15 studies
PCS	0·59 (95% CI 0·00-1910·06), n=2 studies
**Treatment response to first-line therapy**	
CR	0·44 (95% CI 0·25-0·77), n=8 studies
CR/PR	0·44 (95% CI 0·17-1·13), n=4 studies
Any response	0·44 (95% CI 0·30-0·65), n=12 studies
CR/PR/SD	0·28 (95% CI 0·04-1·92), n=2 studies
Incomplete response	0·30 (95% CI 0·05-1·69), n=1 study
NR	0·48 (95% CI 0·20-1·14), n=5 studies
**Use of brain baseline brain CT/MRI**	
Yes	0·51 (95% CI 0·34-0·75), n=12 studies
No	0·33 (95% CI 0·11-1·02)· n=2 studies
NR	0·38 (95% CI 0·20-0·75), n=6 studies
**MRI confirmation of no brain metastases at restaging**	
Yes	0·28 (95% CI 0·01-3·71), n=2 studies
No	0·40 (95% CI 0·29-0·55), n=4 studies
NR	0·51 (95% CI 0·35-0·73), n=14 studies
**Use of platinum-based therapy**	
Yes	0·39 (95% CI 0·28-0·55), n=12 studies
No	0·65 (95% CI 0·05-8·71), n=3 studies
Only in some patients	0·44 (95% CI 0·14-1·38), n=4 studies
NR	0·74 (95% CI 33-1·66), n=1 study
**AHRQ***	
Good	0·24 (95% CI 0·01-4·17), n=2 studies
Fair	0·18 (95% CI 0·03-0·83), n= 1 study
Poor	0·48 (95% CI 0·34-0·67), n=14 studies
**RoB****	
Some concerns	0·60 (95% CI 0·22-1·60), n=3 studies

AHRQ = Agency for Health Research and Quality; CR = complete response; CT = computed tomography; MRI = magnetic resonance imaging; NR = not reported; PR = partial response; PCS = prospective cohort study; SD = stable disease; RCS = retrospective cohort study; RCT = randomided controlled trial; RoB = risk of bias

*AHRQ only reported for non-randomised trials

**RoB only reported for randomised controlled trials

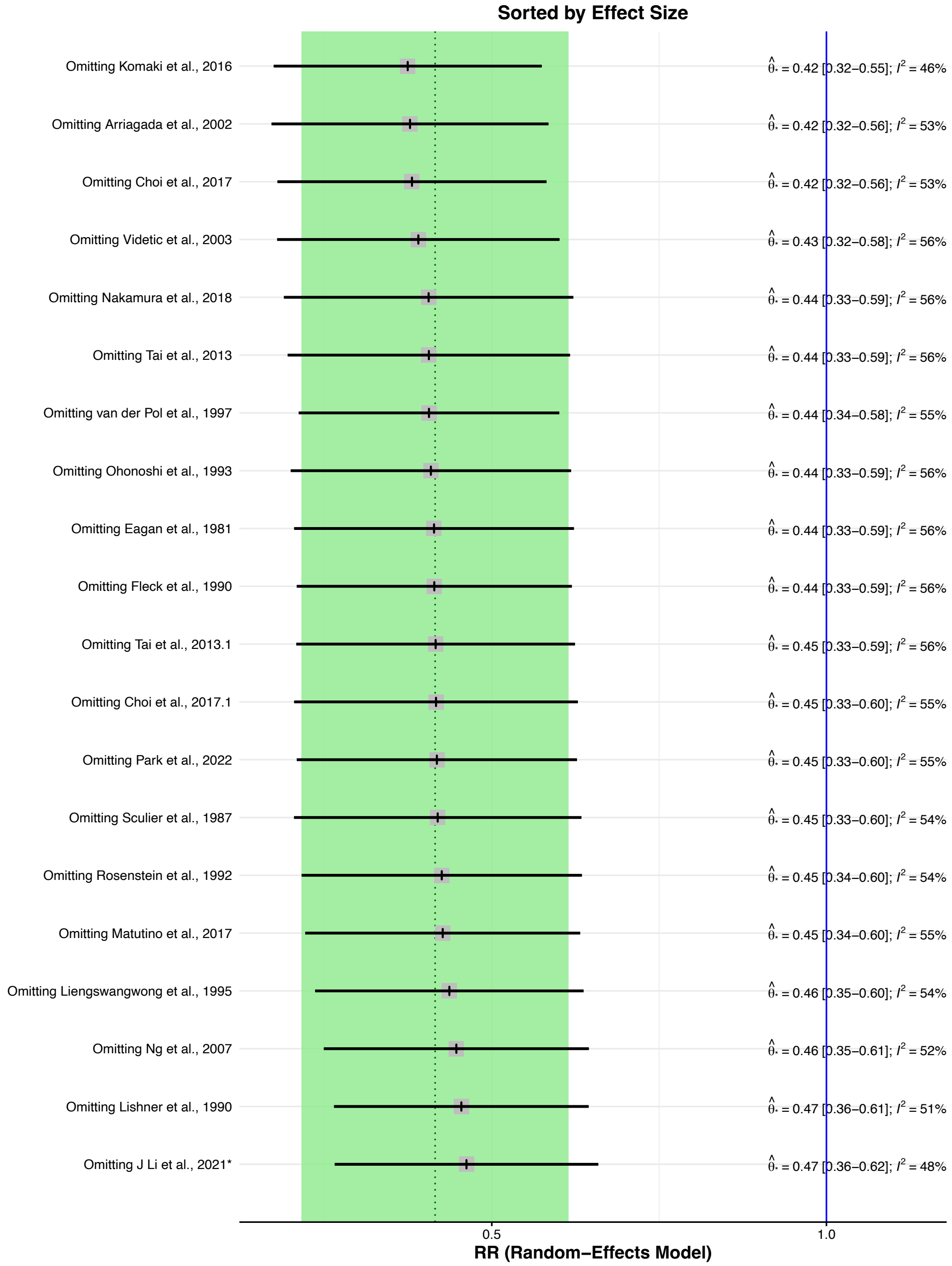

Figure S20. Forest plots for leave-out-one analysis sorted from low to high effect size for the secondary outcome of incidence of brain metastases as first site of recurrence. The plot shows the recalculated pooled effects, with one study omitted each time. The dashed line and shaded area represent the estimated pooled effect size and the 95% confidence interval of the original meta-analysis, respectively. No substantial changes in heterogeneity or effect size were observed.

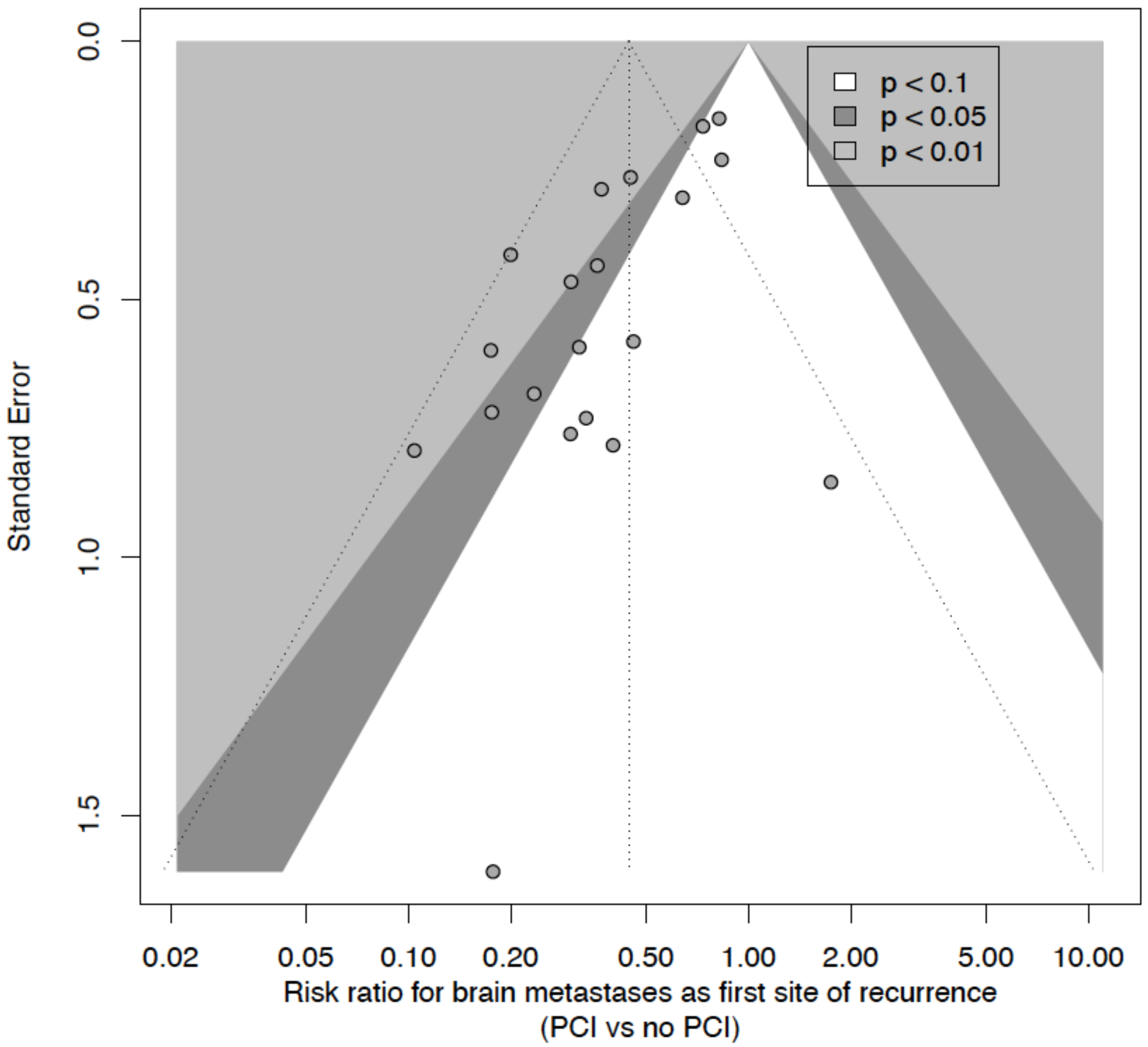

Figure S21. Funnel plot for publication bias among studies comparing PCI with no PCI and reporting on incidence of brain metastases as first site of recurrence.

PCI = prophylactic cranial irradiation; vs = versus

Narrative synthesis

Three studies reported on the brain as the first site of metastatic recurrence with two finding reduced incidence [9,124] and one not reporting on statistical significance[165]. One study radiographically confirmed absence of brain metastases using computed tomography imaging after first line therapy.[9]

Secondary outcomes: neurocognitive decline and adverse events

We identified 15[9,44,63,74,76,110,120,121,139,147,153,155,156,163,206] studies that assessed neurocognitive decline between in relation to receipt of PCI (supplementary excel, "Neurocognitive decline and AEs"). None were amenable to meta-analysis. Two studies found significant reduction in neurocognitive function in patients who received PCI[121,153], 4 found no significant difference[44,63,156,163], and 9 did not report on statistical significance[9,74,76,110,120,139,147,155,206]. Twenty[20,25,29,56-58,63,69,74,78,120,127,129,138-140,142,156,163,189] studies reported on adverse events associated with administration of PCI. Five of these found no grade 3+ adverse events following administration of PCI[20,25,69,74,129] (Appendix 2, Table S12).

References

1. Ago T, Liem JSK, Yu E, et al. Prophylactic cranial irradiation revisited: Cost-effectiveness and quality of life in small-cell lung cancer. *International Journal of Radiation Oncology Biology Physics* 2002; **52**(1): 68-74.
2. Ahn S, Jeong J, Kim Y, et al. Time to the End of Thoracic Radiotherapy Affects to Survival Outcomes Greater than Radiation Dose in Limited Stage Small Cell Lung Cancer. *Journal of Thoracic Oncology* 2018; **13**(10 Supplement): S575-S6.
3. Ainsworth N, Bulusu R, Fife K, et al. Multicentre study of overall survival, incidence of brain metastases and use of prophylactic cranial irradiation in patients with small cell lung cancer from 2005-2009. *Journal of Thoracic Oncology* 2011; **6**(6 SUPPL. 2): S642-S3.
4. Akyurek S, Onal C, Cagar A, Hicsonmez A, Andrieu MN, Kurtman C. Mid-course thoracic radiotherapy with cisplatin-etoposide chemotherapy in limited-stage small-cell lung cancer. *Medical oncology (Northwood, London, England)* 2006; **23**(4): 499-505.
5. Al Farsi A, Swaminath A, Ellis P. Patterns of relapse in Small Cell Lung Cancer (SCLC): A retrospective analysis of outcomes from a Single Canadian Center. *Journal of Thoracic Oncology* 2017; **12**(1 Supplement 1): S727-S8.
6. Alexopoulos, Vaslamatzis, Patilla, Taranto. Central Nervous System Involvement and the Role of Prophylactic Cranial Irradiation in Small Cell Lung Cancer. *The oncologist* 1997; **2**(3): 153-9.
7. An C, Jing W, Zhang Y, et al. Thoracic radiation therapy could give survival benefit to elderly patients with extensive-stage small-cell lung cancer. *Future oncology (London, England)* 2017; **13**(13): 1149-58.
8. Aroney RS, Aisner J, Wesley MN, et al. Value of prophylactic cranial irradiation given at complete remission in small cell lung carcinoma. *Cancer treatment reports* 1983; **67**(7-8): 675-82.
9. Arriagada R, Le Chevalier T, Borie F, et al. Prophylactic cranial irradiation for patients with small-cell lung cancer in complete remission. *Journal of the National Cancer Institute* 1995; **87**(3): 183-90.
10. Arriagada R, Le Chevalier T, Riviere A, et al. Patterns of failure after prophylactic cranial irradiation in small-cell lung cancer: Analysis of 505 randomized patients. *Annals of Oncology* 2002; **13**(5): 748-54.
11. Asaad T, Asaad Trabolsi JMBER, Mount Sinai Medical Center MBFL, Mount Sinai Medical Center MBFLUS, Mount Sinai Comprehensive Cancer Center MBFL. Evaluating the use of prophylactic cranial irradiation in small cell lung cancer. %U https://meetinglibrary.asco.org/record/159998/abstract %W ASCO Meeting Library. 2018.
12. Atci MM, Sakin A, Uysal E, Aksaray F, Selvi O, Can O. Survival and Prognostic Factors in Limited-stage Small-cell Lung Cancer. *J Coll Physicians Surg Pak* 2021; **31**(12): 1433-7.
13. Aufderstrasse S, El Shafie RA, Bernhardt D, et al. Impact of inflammatory markers on survival in patients with limited disease small-cell lung cancer undergoing chemoradiotherapy. *Cancer management and research* 2018; **10**: 6563-9.
14. Ayala de Miguel P, Enguix-Riego MV, Cacicedo J, et al. Prognostic value of the TGFbeta1 rs4803455 single nucleotide polymorphism in small cell lung cancer. *Tumori* 2020: 300891620946841.

15. Aynaci O, Canyilmaz E, Serdar L, Kandaz M, Bahat Z, Yoney A. Survival and prognostic factors in limited stage small cell lung cancer: A retrospective study from northeast Turkey. *Journal of cancer research and therapeutics* 2016; **12**(1): 238-43.
16. Bains Y, Lu J, Etuk B, et al. Twice-daily prophylactic cranial irradiation for patients with limited disease small-cell lung cancer with complete response to chemotherapy and consolidative radiotherapy: Report of a single institutional phase II trial. *American Journal of Clinical Oncology: Cancer Clinical Trials* 2001; **24**(3): 290-5.
17. Bang A, Kendal WS, Cook G, MacRae RM, Laurie SA. Prophylactic Cranial Irradiation in Extensive Stage Small Cell Lung Cancer: Outcomes at a Comprehensive Cancer Centre. *International Journal of Radiation Oncology Biology Physics* 2018; **101**(5): 1133-40.
18. Bayman E, Etiz D, Akcay M, Ak G. Timing of thoracic radiotherapy in limited stage small cell lung cancer: results of early versus late irradiation from a single institution in Turkey. *Asian Pacific journal of cancer prevention : APJCP* 2014; **15**(15): 6263-7.
19. Bettington CS, Bryant G, Hickey B, Tripcony L, Pratt G, Fay M. A retrospective analysis of survival outcomes for two different radiotherapy fractionation schedules given in the same overall time for limited stage small cell lung cancer. *Journal of medical imaging and radiation oncology* 2013; **57**(1): 105-12.
20. Bischof M, Debus J, Herfarth K, et al. Surgery and chemotherapy for small cell lung cancer in stages I-II with or without radiotherapy. *Strahlentherapie und Onkologie : Organ der Deutschen Rontgengesellschaft [et al]* 2007; **183**(12): 679-84.
21. Boskovic T, Vitorovic S, Secen N, Sevo M, Culibrk T, Bokan D. EP1.12-04 Can Prophylactic Cranial Irradiation Reduce Neurological Symptoms in Patients with Small Cell Lung Cancer? *Journal of Thoracic Oncology* 2019; **14**(10 Supplement): S1017.
22. Bravo RG, Autor CA, De La Puente Orteu B, et al. P2.16-34 Is Prophylactic Cranial Irradiation Useful in Real World? *Journal of Thoracic Oncology* 2019; **14**(10 Supplement): S878-S9.
23. Bruni A, Lanfranchi B, Rubino L, et al. Profilattic cranial irradiation (PCI) following toracic CHT-RT for small cell lung cancer: Retrospective analysis of 182 consecutive patients. *Journal of Thoracic Oncology* 2015; **10**(9 SUPPL. 2): S743.
24. Feasibility of omitting clinical target volume for limited-disease small cell lung cancer treated with chemotherapy and intensity-modulated radiotherapy. *Radiation Oncology* 2014; **9**(1): 17.
25. Cao K-J, Huang H-y, Tu M-c, Pan G-y. Long-term results of prophylactic cranial irradiation for limited-stage small-cell lung cancer in complete remission. *Chinese medical journal* 2005; **118**(15): 1258-62.
26. Cetingoz R, Cetinayak HO, Kinay M, et al. Prognostic factors in limited-stage small cell lung cancer of patients treated with combined modality approach. *Journal of BUON* 2006; **11**(1): 31-7.
27. Chen X, Fang J, Nie J, et al. [Multivariate analysis of prognostic factors in the eldly patients with small cell lung cancer: a study of 160 patients]. *Zhongguo fei ai za zhi = Chinese journal of lung cancer* 2014; **17**(1): 15-23.
28. Chen X-R, Liang J-Z, Ma S-X, et al. Consolidation chemotherapy improves progression-free survival in stage III small-cell lung cancer following concurrent chemoradiotherapy: a retrospective study. *OncoTargets and therapy* 2016; **9**: 5729-36.

29. Chen Y, Li J, Hu Y, et al. Prophylactic cranial irradiation could improve overall survival in patients with extensive small cell lung cancer: A retrospective study. *Strahlentherapie und Onkologie* 2016; **192**(12): 905-12.
30. Chen M-Y, Hu X, Xu Y-J, Chen M. The impact of prophylactic cranial irradiation for post-operative patients with limited stage small cell lung cancer. *Medicine* 2018; **97**(44): e13029.
31. Chen Y, Jin Y, Hu X, Chen M. Effect of chemoradiotherapy on the proportion of circulating lymphocyte subsets in patients with limited-stage small cell lung cancer. *Cancer immunology, immunotherapy : CII* 2021.
32. Chen Y, Paz-Ares L, Reinmuth N, et al. Impact of Brain Metastases on Treatment Patterns and Outcomes With First-Line Durvalumab Plus Platinum-Etoposide in Extensive-Stage SCLC (CASPIAN): A Brief Report. *JTO Clin Res Rep* 2022; **3**(6): 100330.
33. Chen Y, Wang Y, Ren F, et al. Prophylactic cranial irradiation (PCI) versus active surveillance in patients with limited-stage small cell lung cancer: a retrospective, multicentre study. *Respir Res* 2022; **23**(1): 274.
34. Choi M, Lee Y, Moon SH, Han J-Y, Kim HT, Lee JS. Effect of Accurate Staging Using Positron Emission Tomography on the Outcomes of Prophylactic Cranial Irradiation in Patients With Limited Stage Small-Cell Lung Cancer. *Clinical lung cancer* 2017; **18**(1): 77-84.
35. Chudyba A, Skora T, Sas-Korczynska B, Luczynska E, Sokolowski A. The retrospective evaluation of prophylactic cranial irradiation in patients treated for limited stage small-cell lung cancer - A single centre study. *Nowotwory* 2018; **68**(5-6): 232-9.
36. Chung J-H, Kang SY, Wu H-G, et al. Risk stratification of symptomatic brain metastases by clinical and FDG PET parameters for selective use of prophylactic cranial irradiation in patients with extensive disease of small cell lung cancer. *Radiotherapy and oncology : journal of the European Society for Therapeutic Radiology and Oncology* 2020; **143**: 81-7.
37. Çimen F, Aloglu M, Düzgün S, Şentürk A, Atikcan Ş, Özmen Ö. What is the effect of tumor diameter, lymph node metastases, and SUVmax value on prognosis in limited-stage small cell lung cancer? *Rev Assoc Med Bras (1992)* 2022; **68**(9): 1252-8.
38. Clark B, Nowicki S, Dunlop D, Mohammed N. Management of small cell lung cancer in the West of Scotland. *Lung Cancer* 2010; **67**(SUPPL 1): S11.
39. Cordeiro D, Xu Z, Shepard M, Sheehan D, Li C, Sheehan J. Gamma Knife radiosurgery for brain metastases from small-cell lung cancer: Institutional experience over more than a decade and review of the literature. *Journal of radiosurgery and SBRT* 2019; **6**(1): 35-43.
40. Cox JD, Petrovich Z, Paig C, Stanley K. Prophylactic cranial irradiation in patients with inoperable carcinoma of the lung: preliminary report of a cooperative trial. *Cancer* 1978; **42**(3): 1135-40.
41. Dawe D, Rittberg R, Bucher O, et al. 1547P Predictors of short-, medium-, and long-term survival with limited stage small cell lung cancer in the real-world. *Annals of Oncology* 2022; **33**: S1254.
42. Dawe DE, Rittberg R, Syed I, et al. Real-world predictors of survival in patients with extensive-stage small-cell lung cancer in Manitoba, Canada: a retrospective cohort study. *Front Oncol* 2023; **13**: 1191855.
43. De Almeida SB, Vitorino M, Goncalves S. MO01.44 Prognostic Factors in Extensive-Stage Small Cell Lung Cancer (SCLC). *Journal of Thoracic Oncology* 2021; **16**(1 Supplement): S34-S5.

44. Depierre A, Jacoulet P, Moro D, et al. Long-term survivors of small-cell lung cancer (SCLC): A French multicenter study. *Annals of Oncology* 1997; **8**(10): 1009-14.
45. Devisetty K, Siker ML, Suboc T, Choong NW, Gore EM. Pattern and extent of failure in limited stage small cell lung cancer: Implications for oligometastatic treatment. *International Journal of Radiation Oncology Biology Physics* 2011; **81**(2 SUPPL. 1): S584.
46. Drpa G, Jakopovic M, Samarzija M, et al. Neutrophil-to-lymphocyte ratio can predict outcome in extensive-stage small cell lung cancer. *Radiology and oncology* 2020; **54**(4): 437-46.
47. Eagan RT, Frytak S, Creagan ET. A case for preplanned thoracic and prophylactic whole brain radiation therapy in limited small-cell lung cancer. *Cancer clinical trials* 1981; **4**(3): 261-6.
48. Eaton BR, Kim S, Marcus DM, et al. Effect of prophylactic cranial irradiation on survival in elderly patients with limited-stage small cell lung cancer. *Cancer* 2013; **119**(21): 3753-60.
49. Elegbede AA, Gibson AJ, Fu H, et al. Real-World Adherence to Guideline-Recommended Treatment for Small Cell Lung Cancer. *American journal of clinical oncology* 2020; **43**(4): 236-42.
50. Elegbede A, Gibson A, Pabani A, Dean M, Bebb G. P63. 13 Long Term Survival Characteristics in SCLC Patients Receiving Atezolizumab and Chemotherapy. *Journal of Thoracic Oncology* 2021; **16**(10): S1187-S8.
51. Ergen SA, Dincbas HF, Oksuz DC, et al. Patterns of failure and the prognostic factors of the patients with LD SCLC according to the TNM staging; TOG-TROD study. *Journal of Thoracic Oncology* 2017; **12**(1 Supplement 1): S723.
52. Eze C, Roengvoraphoj O, Niyazi M, et al. Treatment response and prophylactic cranial irradiation are important prognostic factors in ld SCLC patients staged with CMRI. *Journal of Thoracic Oncology* 2017; **12**(1 Supplement 1): S1470-S1.
53. Faramand A, Niranjan A, Kano H, Flickinger J, Lunsford LD. Primary or salvage stereotactic radiosurgery for brain metastatic small cell lung cancer. *Journal of neuro-oncology* 2019; **144**(1): 217-25.
54. Farooqi AS, Holliday EB, Allen PK, Wei X, Cox JD, Komaki R. Prophylactic cranial irradiation after definitive chemoradiotherapy for limited-stage small cell lung cancer: Do all patients benefit? *Radiotherapy and oncology : journal of the European Society for Therapeutic Radiology and Oncology* 2017; **122**(2): 307-12.
55. Farris MK, Mix M, Wang X, et al. MA18.04 Prognostic Factors in Limited-Stage Small Cell Lung Cancer (LSCLC) : Secondary Analysis of CALGB 30610 (Alliance)/RTOG 0538. *Journal of Thoracic Oncology* 2023; **18**(11, Supplement): S166-S7.
56. Fleck JF, Einhorn LH, Lauer RC, Schultz SM, Miller ME. Is prophylactic cranial irradiation indicated in small-cell lung cancer? *Journal of clinical oncology : official journal of the American Society of Clinical Oncology* 1990; **8**(2): 209-14.
57. Frytak S, Shaw JN, O'Neill BP, et al. Leukoencephalopathy in small cell lung cancer patients receiving prophylactic cranial irradiation. *American journal of clinical oncology* 1989; **12**(1): 27-33.
58. Geddes DM, Dones L, Hill E, et al. Quality of life during chemotherapy for small cell lung cancer: assessment and use of a daily diary card in a randomized trial. *European journal of cancer (Oxford, England : 1990)* 1990; **26**(4): 484-92.
59. Ghanta S, Keller A, Rodríguez-López JL, Patel A, Beriwal S. Utility of Prophylactic Cranial Irradiation for Limited Stage Small Cell Lung Cancer in the Modern Era with Magnetic Resonance Imaging Surveillance. *Clin Oncol (R Coll Radiol)* 2021; **33**(8): e323-e30.

60. Giaccone G, Debruyne C, Felip E, et al. Phase III study of adjuvant vaccination with Bec2/bacille Calmette-Guerin in responding patients with limited-disease small-cell lung cancer (European Organisation for Research and Treatment of Cancer 08971-08971B; Silva Study). *Journal of clinical oncology : official journal of the American Society of Clinical Oncology* 2005; **23**(28): 6854-64.
61. Go S-I, Keam B, Kim TM, et al. Clinical significance of downstaging in patients with limited-disease small-cell lung cancer. *Clinical lung cancer* 2014; **15**(2): e1-e6.
62. Gross AJ, Kharouta MZ, Chaung KV, et al. Prophylactic Cranial Irradiation (PCI) and Consolidative Thoracic Radiation (TRT) in Extensive Stage Small Cell (ES-SCLC) Lung Cancer. *International Journal of Radiation Oncology Biology Physics* 2020; **108**(3 Supplement): e161.
63. Gregor A, Cull A, Stephens RJ, et al. Prophylactic cranial irradiation is indicated following complete response to induction therapy in small cell cancer: Results of a multicentre randomised trial. *European Journal of Cancer* 1997; **33**(11): 1752-8.
64. Giuliani M, Sun A, Bezjak A, et al. Utilization of prophylactic cranial irradiation in patients with limited stage small cell lung carcinoma. *Cancer* 2010; **116**(24): 5694-9.
65. Guo Y, Yang L, Liu L, et al. Comparative study of clinicopathological characteristics and prognosis between combined and pure small cell lung cancer (SCLC) after surgical resection. *Thoracic cancer* 2020; **11**(10): 2782-92.
66. Han B, Xu J, Qiao R, et al. Prophylactic Cranial Irradiation Can Not Provide Survival Benefits for Resected Small Cell Lung Cancer Without Lymph Node Involvement. *Journal of Thoracic Oncology* 2018; **13**(10 Supplement): S797.
67. Harden SV, Ainsworth N, Saunders D, Magee L. Small cell lung cancer overall survival, incidence of brain metastases and use of prophylactic cranial irradiation for patients treated in Cambridge during 2005-2009. *Lung Cancer* 2011; **71**(SUPPL. 1): S42.
68. Held MK, Hansen O, Schytte T, et al. Outcomes of prophylactic cranial irradiation in patients with small cell lung cancer in the modern era of baseline magnetic resonance imaging of the brain. *Acta Oncol* 2022; **61**(2): 185-92.
69. Herrmann MKA, Bloch E, Overbeck T, et al. Mediastinal radiotherapy after multidrug chemotherapy and prophylactic cranial irradiation in patients with SCLC--treatment results after long-term follow-up and literature overview. *Cancer radiotherapie : journal de la Societe francaise de radiotherapie oncologique* 2011; **15**(2): 81-8.
70. Hett KL, Tanguay JS, Button M. Survival in stage IV small cell lung cancer and effect of prophylactic cranial irradiation. *Lung Cancer* 2014; **83**(SUPPL. 1): S67-S8.
71. Hu X, Xia B, Bao Y, et al. Timing of thoracic radiotherapy is more important than dose intensification in patients with limited-stage small cell lung cancer: a parallel comparison of two prospective studies. *Der Zeitpunkt der thorakalen Strahlentherapie ist wichtiger als die Dosisintensivierung bei Patienten mit limitiertem Stadium des kleinzelligen Lungenkarzinoms: Ein paralleler Vergleich von zwei prospektiven Studien* 2020; **196**(2): 172-81.
72. Hu X, Chen MY, Zheng SH, et al. EP13.04-03 The Impact of PCI on the Prognosis of Limited-stage SCLC Patients in the Era of MRI Surveillance. *Journal of Thoracic Oncology* 2023; **18**(11, Supplement): S699.
73. Hwang E, Williams J, Venchiarutti R, Wong W, Lewis C. Limited stage small cell lung cancer: Patterns of care and outcomes of a single institution over 15 years. *Journal of Thoracic Oncology* 2017; **12**(1 Supplement 1): S727.

74. Inoue Y, Tsujino K, Sulaiman NS, et al. Re-evaluation of prophylactic cranial irradiation in limited-stage small cell lung cancer: a propensity score matched analysis. *J Radiat Res* 2021; **62**(5): 877-83.
75. Iqbal MS, Carlow J, McDonald F, et al. Sequential chemotherapy followed by radical thoracic radiotherapy (50 Gy in 25 fractions) in limited stage small cell lung cancer. *Ecancermedicalscience* 2020; **14**: 1019.
76. Jacobs RH, Greenburg A, Bitran JD. A 10-year experience with combined modality therapy for Stage III small cell lung carcinoma. *Cancer* 1986; **58**(10): 2177-84.
77. Janardanan Nair V, Sirisegaram A, Nicholas G, Mallick R, Laurie S, MacRae R. Higher dose thoracic radiation therapy is associated with improved overall survival in limited-stage small cell lung cancer: Analysis of the 15-year experience from a retrospective cohort of 296 patients. *International Journal of Radiation Oncology Biology Physics* 2012; **84**(3 SUPPL. 1): S577.
78. Jat RR, Rai RK, Jakhar SL, Beniwal SK, Sharma N, Kumar HS. Experience of prophylactic cranial irradiation in extensive stage small cell lung cancer at a regional cancer center in India. *Annals of Oncology* 2019; **30**: ii24-.
79. Jing W, Wang H, An C, Yu J, Zhu H. PD-l1 expression level could predict the survival of patients with resectable small cell lung cancer. *Journal of Thoracic Oncology* 2017; **12**(1 Supplement 1): S1484-S5.
80. Jo KW, Kong DS, Lim DH, Ahn YC, Nam D-H, Lee J-I. The role of radiosurgery in patients with brain metastasis from small cell lung carcinoma. *Journal of Korean Neurosurgical Society* 2011; **50**(2): 99-102.
81. Jo IY, Noh JM, Ahn YC, Pyo H, Oh D. Is Post-chemotherapy tumor volume sufficient RT target volume in patients with LD-SCLC? *Journal of Thoracic Oncology* 2017; **12**(1 Supplement 1): S1044.
82. Jones GS, Khakwani A, Pascoe A, et al. Factors associated with survival in small cell lung cancer: an analysis of real-world national audit, chemotherapy and radiotherapy data. *Ann Palliat Med* 2021; **10**(4): 4055-68.
83. Jove J, Arellano A, Ballester R, et al. How does influence changes in standard treatment in outcome results for limited stage small-cell lung cancer patients in a radiation oncology department? *Journal of Thoracic Oncology* 2011; **6**(6 SUPPL. 2): S1351.
84. Kamath SS, McCarley DL, Zlotecki RA. Decreased metastasis and improved survival with early thoracic radiotherapy and prophylactic cranial irradiation in combined-modality treatment of limited-stage small cell lung cancer. *Radiation oncology investigations* 1998; **6**(5): 226-32.
85. Kang HS, Lim JU, Yeo CD, Park CK, Lee SH, Kim SJ. Characteristics and clinical outcomes of patients with nonsmoking small cell lung cancer in Korea. *BMC Pulm Med* 2022; **22**(1): 200.
86. Kasmann L, Janssen S, Rades D. Karnofsky Performance Score, Radiation Dose and Nodal Status Predict Survival of Elderly Patients Irradiated for Limited-disease Small-cell Lung Cancer. *Anticancer research* 2016; **36**(8): 4177-80.
87. Kasmann L, Bolm L, Janssen S, Rades D. Prognostic Factors and Treatment of Early-stage Small-cell Lung Cancer. *Anticancer research* 2017; **37**(3): 1535-7.
88. Kasmann L, Bolm L, Rades D, Schild SE, Janssen S. Neutrophil-to-Lymphocyte Ratio Predicts Outcome in Limited Disease Small-cell Lung Cancer. *Lung* 2017: 1-8.

89. Keller A, Rodriguez-Lopez JL, Beriwal S. Utilization of Prophylactic Cranial Irradiation for Extensive Stage Small Cell Lung Cancer-Is There a Benefit in the MRI Screening Era? *International Journal of Radiation Oncology Biology Physics* 2020; **108**(3 Supplement): e82-e3.
90. Keller A, Ghanta S, Rodríguez-López JL, Patel A, Beriwal S. Utility of Prophylactic Cranial Irradiation for Extensive-Stage Small-Cell Lung Cancer in the MRI Screening Era. *Clin Lung Cancer* 2021; **22**(6): e808-e16.
91. Khaira M, Schellenberg D, Lee CW, Joffres M. An examination of the incidence and treatment of brain metastases after prophylactic cranial irradiation (PCI) forsmall cell lung cancer (SCLC). *Radiotherapy and Oncology* 2010; **96**(SUPPL. 2): S56.
92. Sequential or concomitant chemotherapy in limited stage small-cell lung cancer. *Swiss medical weekly* 2011; **141**(MAY).
93. Kim H-J, Choi C-M, Kim S-G. The Younger Patients Have More Better Prognosis in Limited Disease Small Cell Lung Cancer. *Tuberculosis and respiratory diseases* 2016; **79**(4): 274-81.
94. Kim TG, Pyo H, Ahn YC, Noh JM, Oh D. Role of prophylactic cranial irradiation for elderly patients with limited-disease small-cell lung cancer: inverse probability of treatment weighting using propensity score. *Journal of radiation research* 2019; **60**(5): 630-8.
95. Koh M, Song SY, Jo JH, et al. The value of prophylactic cranial irradiation in limited-stage small cell lung cancer: Should it always be recommended? *Radiation oncology journal* 2019; **37**(3): 156-65.
96. Komaki R, Cox JD, Holoye PY, Byhardt RW. Changes in the relative risk and sites of central nervous system metastasis with effective combined chemotherapy and radiation therapy for small cell carcinoma of the lung. *American journal of clinical oncology* 1983; **6**(5): 515-21.
97. Komaki RU, Wei X, Allen PK, et al. Does prophylactic cranial irradiation improve overall survival of elderly patients with limited-stage small cell lung cancer? *Journal of Thoracic Oncology* 2015; **10**(9 SUPPL. 2): S627.
98. Komaki RU, Allen PK, Wei X, Welsh JW, Lin SH, Cox JD. Completing thoracic radiation therapy with concurrent chemotherapy within 6 weeks is important for reducing distant disease in patients with limited-stage small cell lung cancer. *International Journal of Radiation Oncology* 2016; **96**(2 Supplement 1): E466-E7.
99. Kosmidis PA, Samantas E, Fountzilas G, Pavlidis N, Apostolopoulou F, Skarlos D. Cisplatin/etoposide versus carboplatin/etoposide chemotherapy and irradiation in small cell lung cancer: A randomized phase III study. *Seminars in oncology* 1994; **21**(3 SUPPL. 6): 23-30.
100. Kou P, Wang H, Yang D, Zhang Y, Yu J. Application of prophylactic cranial irradiation in limited-stage small-cell lung cancer: which patients could benefit? *Future oncology (London, England)* 2019; **15**(1): 3237-45.
101. Lee TH, Chung JH, Wu HG, et al. Efficacy of Prophylactic Cranial Irradiation According to the Risk of Extracranial Recurrence in Limited-Stage Small Cell Lung Cancer. *Cancer Res Treat* 2023; **55**(3): 875-84.
102. Levy A, Le Pechoux C, Mistry H, et al. Prophylactic Cranial Irradiation for Limited-Stage Small-Cell Lung Cancer Patients: secondary Findings From the Prospective Randomized Phase 3 CONVERT Trial. *Journal of Thoracic Oncology* 2018; **(no pagination)**.
103. Lewinski T, Zulawski M, Meder J, et al. [Elective (prophylactic) brain irradiation in small cell lung cancer patients treated with chemotherapy combined with surgery]. *Elektywne*

(profilaktyczne) napromienianie mozgu u chorych na drobnokomorkowego raka pluca leczonych chemicznie w skojarzeniu z chirurgia 1990; **40**(4): 254-9.
104. Li J, Chen P, Bao Q-L, et al. Survival and prognostic factors in small cell lung cancer. *Medical Oncology* 2010; **27**(1): 73-81.
105. Li J, Ding C, Yang C, Wang S, Qiao X. Prophylactic cranial irradiation confers favourable prognosis for patients with limited-stage small cell lung cancer in the era of MRI: A propensity score-matched analysis. *J Med Imaging Radiat Oncol* 2021; **65**(6): 778-85.
106. Liengswangwong V, Bonner JA, Shaw EG, et al. Prophylactic cranial irradiation in limited-stage small cell lung cancer. *Cancer* 1995; **75**(6): 1302-9.
107. Lim YJ, Song C, Kim HJ. Survival impact of prophylactic cranial irradiation in small-cell lung cancer in the modern era of magnetic resonance imaging staging. *Radiat Oncol* 2022; **17**(1): 26.
108. Lim W-T, Leong S-S, Tan E-H, et al. Survival of small-cell lung cancer and its determinants of outcome in Singapore. *Annals of the Academy of Medicine Singapore* 2007; **36**(3): 181-8.
109. Lin X, Xiao Z, Hu Y, Zhang X, Fan W. Combining 18F-FDG PET/CT and Serum Lactate Dehydrogenase for Prognostic Evaluation of Small Cell Lung Cancer. *Frontiers in pharmacology* 2020; **11**: 592768.
110. Lishner M, Feld R, Payne DG, et al. Late neurological complications after prophylactic cranial irradiation in patients with small-cell lung cancer: the Toronto experience. *Journal of clinical oncology : official journal of the American Society of Clinical Oncology* 1990; **8**(2): 215-21.
111. Liu X, Li X, Pang Y, Li Q, Wang X, Shen W. The results of treatment in 100 patients with limited-stage small cell lung cancer. *Cancer* 1993; **71**(2): 326-31.
112. Liu X, Jiang T, Li W, et al. Characterization of never-smoking and its association with clinical outcomes in Chinese patients with small-cell lung cancer. *Lung cancer (Amsterdam, Netherlands)* 2018; **115**: 109-15.
113. Lok BH, Ma J, Foster A, et al. Factors influencing the utilization of prophylactic cranial irradiation in patients with limited-stage small cell lung cancer. *Advances in radiation oncology* 2017; **2**(4): 548-54.
114. Longo V, Pizzutilo P, Catino A, et al. Prognostic factors for survival in extensive-stage small cell lung cancer: An Italian real-world retrospective analysis of 244 patients treated over the last decade. *Thorac Cancer* 2022; **13**(24): 3486-95.
115. Ludbrook JJS, Joe H, Lim J, et al. Do age and comorbidity impact treatment allocation and outcomes in limited stage small-cell lung cancer? A community-based population analysis. *International Journal of Radiation Oncology Biology Physics* 2003; **55**(5): 1321-30.
116. Ma X, Zhang Z, Chen X, et al. Prognostic factor analysis of patients with small cell lung cancer: Real-world data from 988 patients. *Thorac Cancer* 2021; **12**(12): 1841-50.
117. Kenneth Macdonald O, Suntharalingam M, Patel S. Evaluation of the use of prophylactic cranial irradiation in small cell lung cancer. *Cancer* 2009; **115**(4): 842-50.
118. Mamesaya N, Wakuda K, Miyawaki E, et al. Efficacy of prophylactic cranial irradiation in patients with limited-disease small-cell lung cancer who were confirmed to have no brain metastasis via magnetic resonance imaging after initial chemoradiotherapy. *Oncotarget* 2018; **9**(25): 17664-74.

119. Manapov F, Klocking S, Niyazi M, et al. Chemoradiotherapy duration correlates with overall survival in limited disease SCLC patients with poor initial performance status who successfully completed multimodality treatment. *Strahlentherapie und Onkologie : Organ der Deutschen Rontgengesellschaft [et al]* 2012; **188**(1): 29-34.
120. Matutino A, Mak MP, Takahashi TK, et al. Prophylactic Cranial Irradiation for Extensive-Stage Small-Cell Lung Cancer: A Retrospective Analysis. *Journal of global oncology* 2018; **4**: 1-7.
121. Nakahara Y, Takagi Y, Okuma Y, et al. Neurotoxicity due to prophylactic cranial irradiation for small-cell lung cancer: A retrospective analysis. *Molecular and clinical oncology* 2015; **3**(5): 1048-52.
122. Nakamura M, Onozawa M, Motegi A, et al. Impact of PCI on prognosis of LD-SCLC through pattern of brain metastases as a first recurrence site. *Journal of Thoracic Oncology* 2017; **12**(11 Supplement 2): S2330.
123. Naidoo M, Lin F, Jameson M, Shelly K, Srivastava A. Prophylactic cranial irradiation in patients with extensive-stage small-cell lung cancer (ES-SCLC): An updated tertiary cancer centre experience. *Annals of Oncology* 2018; **29**(Supplement 9).
124. Ng M, Chong J, MacManus M, et al. Tolerability of accelerated chest irradiation and impact on survival of prophylactic cranial irradiation in patients with limited-stage small cell lung cancer: Review of a single institution's experience. *Journal of Thoracic Oncology* 2007; **2**(6): 506-13.
125. Nicholls L, Keir GJ, Murphy MA, Mai T, Lehman M. Prophylactic cranial irradiation in small cell lung cancer: A single institution experience. *Asia-Pacific journal of clinical oncology* 2016; **12**(4): 415-20.
126. Niiranen A, Holsti P, Salmo M. Treatment of small cell lung cancer. Two-drug versus four-drug chemotherapy and loco-regional irradiation with or without prophylactic cranial irradiation. *Acta oncologica (Stockholm, Sweden)* 1989; **28**(4): 501-5.
127. Ohonoshi T, Ueoka H, Kawahara S, et al. Comparative study of prophylactic cranial irradiation in patients with small cell lung cancer achieving a complete response: a long-term follow-up result. *Lung cancer (Amsterdam, Netherlands)* 1993; **10**(1-2): 47-54.
128. Ozawa Y, Omae M, Fujii M, et al. Management of brain metastasis with magnetic resonance imaging and stereotactic irradiation attenuated benefits of prophylactic cranial irradiation in patients with limited-stage small cell lung cancer. *BMC cancer* 2015; **15**: 589.
129. Park S, Noh JM, Choi YL, et al. Durvalumab with chemoradiotherapy for limited-stage small-cell lung cancer. *Eur J Cancer* 2022; **169**: 42-53.
130. Park SY, Park S, Lee GD, et al. The Role of Adjuvant Therapy Following Surgical Resection of Small Cell Lung Cancer: A Multi-Center Study. *Cancer Res Treat* 2023; **55**(1): 94-102.
131. Patel SA, Bowen SR, Gamboa C, Baik C, Zeng J. Improved survival with prophylactic cranial irradiation in modern cohort of limited-stage small cell lung cancer patients with MRI brain imaging. *International Journal of Radiation Oncology Biology Physics* 2013; **87**(2 SUPPL. 1): S552.
132. Pezzi TA, Fang P, Gjyshi O, et al. Rates of Overall Survival and Intracranial Control in the Magnetic Resonance Imaging Era for Patients With Limited-Stage Small Cell Lung Cancer With and Without Prophylactic Cranial Irradiation. *JAMA Netw Open* 2020; **3**(4): e201929.

133. Prelaj A, Del Bene G, Berrios JRG, et al. Evaluation of the efficacy of cisplatin-etoposide and the role of thoracic radiotherapy and prophylactic cranial irradiation in LCNEC. *ERJ open research* 2017; **3**(1): 00128-2016.
134. Qi C, Li W, Li H, et al. Benefits of Prophylactic Cranial Irradiation in the MRI Era for Patients With Limited Stage Small Cell Lung Cancer. *Front Oncol* 2022; **12**: 833478.
135. Qiu G, Du X, Zhou X, et al. Prophylactic cranial irradiation in 399 patients with limited-stage small cell lung cancer. *Oncology letters* 2016; **11**(4): 2654-60.
136. Resio BJ, Hoag J, Chiu A, et al. Prophylactic cranial irradiation is associated with improved survival following resection for limited stage small cell lung cancer. *Journal of thoracic disease* 2019; **11**(3): 811-8.
137. Rosen ST, Makuch RW, Lichter AS, et al. Role of prophylactic cranial irradiation in prevention of central nervous system metastases in small cell lung cancer. Potential benefit restricted to patients with complete response. *American Journal of Medicine* 1983; **74**(4): 615-24.
138. Rosenstein M, Armstrong J, Kris M, et al. A reappraisal of the role of prophylactic cranial irradiation in limited small cell lung cancer. *International journal of radiation oncology, biology, physics* 1992; **24**(1): 43-8.
139. Rubenstein JH, Dosoretz DE, Katin MJ, et al. Low doses of prophylactic cranial irradiation effective in limited stage small cell carcinoma of the lung. *International journal of radiation oncology, biology, physics* 1995; **33**(2): 329-37.
140. Rule WG, Ashman JB, Vora SA, et al. Prophylactic cranial irradiation in elderly patients with small cell lung cancer: Findings from a North Central Cancer Treatment Group pooled analysis. *Journal of geriatric oncology* 2015; **6**(2): 119-26.
141. Sakin A, Yasar N, Arici S, et al. Effect of Pretreatment Platelet Parameters on Survival in Limited Disease Small Cell Lung Cancer. *Asian Pacific journal of cancer prevention : APJCP* 2019; **20**(6): 1879-85.
142. Salama JK, Gu L, Wang X, et al. Positive interaction between prophylactic cranial irradiation and maintenance sunitinib for untreated extensive-stage small cell lung cancer patients after standard chemotherapy: A secondary analysis of CALGB 30504 (ALLIANCE). *Journal of Thoracic Oncology* 2016; **11**(3): 361-9.
143. Sas-Korczynska B, Korzeniowski S, Wojcik E. Comparison of the effectiveness of "late" and "early" prophylactic cranial irradiation in patients with limited-stage small cell lung cancer. *Strahlentherapie und Onkologie : Organ der Deutschen Rontgengesellschaft [et al]* 2010; **186**(6): 315-9.
144. SchnOller L, Kasmann L, Taugner J, Abdo R, Eze C, Manapov F. Prognostic Role of Lung Immune Scores for Prediction of Survival in Limited-stage Small Cell Lung Cancer. *In vivo (Athens, Greece)* 2021; **35**(2): 929-35.
145. Scotti V, Meattini I, Franzese C, et al. Radiotherapy timing in the treatment of limited-stage small cell lung cancer: The impact of thoracic and brain irradiation on survival. *Tumori* 2014; **100**(3): 289-95.
146. Sculier JP, Feld R, Evans WK, et al. Neurologic disorders in patients with small cell lung cancer. *Cancer* 1987; **60**(9): 2275-83.

147. Seydel HG, Creech R, Pagano M. Prophylactic versus no brain irradiation in regional small cell lung carcinoma. *American Journal of Clinical Oncology: Cancer Clinical Trials* 1985; **8**(3): 218-23.
148. Sharma S, McMillan MT, Doucette A, et al. Effect of Prophylactic Cranial Irradiation on Overall Survival in Metastatic Small-Cell Lung Cancer: A Propensity Score-Matched Analysis. *Clinical lung cancer* 2018; **19**(3): 260.
149. Shaw EG, Su JQ, Eagan RT, Jett JR, Maksymiuk AW, Deigert FA. Prophylactic cranial irradiation in complete responders with small-cell lung cancer: analysis of the Mayo Clinic and North Central Cancer Treatment Group data bases. *Journal of clinical oncology : official journal of the American Society of Clinical Oncology* 1994; **12**(11): 2327-32.
150. Sheikh S, Dey A, Datta S, et al. Role of radiation in extensive stage small cell lung cancer: a National Cancer Database registry analysis. *Future Oncol* 2021; **17**(21): 2713-24.
151. Shioyama Y, Nagata Y, Kimura T, et al. Multi-institutional retrospective study of stereotactic body radiation therapy for stage i small cell lung cancer: Japan radiation oncology study group (JROSG). *International Journal of Radiation Oncology Biology Physics* 2015; **93**(3 SUPPL. 1): S101.
152. Shirvani SM, Juloori A, Gomez D, et al. Patterns of failure for limited-stage small cell lung cancer following definitive radiation therapy in the modern ERA. *International Journal of Radiation Oncology Biology Physics* 2012; **84**(3 SUPPL. 1): S595-S6.
153. Simo M, Vaquero L, Ripolles P, et al. Brain damage following prophylactic cranial irradiation in lung cancer survivors. *Brain imaging and behavior* 2016; **10**(1): 283-95.
154. Siker ML, Devisetty K, Suboc T, Choong NW, Gore EM. Extensive stage small-cell lung cancer: Patterns of failure after chest and cranial radiotherapy. *International Journal of Radiation Oncology Biology Physics* 2011; **81**(2 SUPPL. 1): S135-S6.
155. Slotman BJ, Njo KH, de Jonge A, Meyer OW, Karim AB. Consolidative thoracic radiotherapy and prophylactic cranial irradiation in limited disease small cell lung cancer. *Lung cancer (Amsterdam, Netherlands)* 1993; **10**(3-4): 199-208.
156. Slotman BJ, Mauer ME, Bottomley A, et al. Prophylactic cranial irradiation in extensive disease small-cell lung cancer: short-term health-related quality of life and patient reported symptoms: results of an international Phase III randomized controlled trial by the EORTC Radiation Oncology and Lung Cancer Groups. *Journal of Clinical Oncology* 2009; **27**(1): 78-84.
157. Soon YY, Koh WY, Leong CN, et al. Adoption of prophylactic cranial irradiation for extensive stage small cell lung cancer: A population based outcomes study. *Journal of Thoracic Oncology* 2017; **12**(11 Supplement 2): S2049.
158. Souhami RL, Law K. Longevity in small cell lung cancer. A report to the Lung Cancer Subcommittee of the United Kingdom Coordinating Committee for Cancer Research. *British journal of cancer* 1990; **61**(4): 584-9.
159. Stanic K, Vrankar M, But-Hadzic J. Consolidation radiotherapy for patients with extended disease small cell lung cancer in a single tertiary institution: impact of dose and perspectives in the era of immunotherapy. *Radiology and oncology* 2020; **54**(3): 353-63.
160. Stolten MD, Clyde JW, Budnik J, Milano MT. Improved Overall Survival in Patients Receiving Prophylactic Cranial Irradiation for Node-Negative Limited Stage Small Cell Lung Cancer. *International Journal of Radiation Oncology Biology Physics* 2020; **108**(3 Supplement): e156.

161. Sun B, Yao N, Wei L, Xu S, Cao J. PO-1185 Development of a nomogram for predicting brain metastasis of small cell lung cancer. *Radiotherapy and Oncology* 2021; **161**: S983-S4.
162. Suzuki R, Lin SH, Wei X, et al. Prognostic significance of pretreatment total lymphocyte count and neutrophil-to-lymphocyte ratio in extensive-stage small-cell lung cancer. *Radiotherapy and oncology : journal of the European Society for Therapeutic Radiology and Oncology* 2018; **126**(3): 499-505.
163. Takahashi T, Harada H, Yamanaka T, et al. Prophylactic cranial irradiation versus observation in patients with extensive-disease small-cell lung cancer: a multicentre, randomised, open-label, phase 3 trial. *The Lancet Oncology* 2017; **18**(5): 663-71.
164. Takamura K, Yokouchi H, Munakata M, et al. Clinical and molecular profiling of surgically resected small cell lung cancer. *Journal of Thoracic Oncology* 2015; **10**(9 SUPPL. 2): S194.
165. Tai P, Assouline A, Joseph K, Stitt L, Yu E. Prophylactic cranial irradiation for patients with limited-stage small-cell lung cancer with response to chemoradiation. *Clinical lung cancer* 2013; **14**(1): 40-4.
166. Tendler S, Lewensohn R, Viktorsson K, et al. P2.12-06 Factors of Importance for Survival After Platinum Re-Challenge in Platinum-Sensitive Small-Cell Lung Cancer Patients. *Journal of Thoracic Oncology* 2019; **14**(10 Supplement): S814.
167. Teng F, Li J, Xing P, Hao X. EP1.12-11 Effect of Treatment Mode on Prognosis of Resectable Limited-Stage Small Cell Lung Cancer. *Journal of Thoracic Oncology* 2019; **14**(10 Supplement): S1020.
168. Teng F, Xing P, Yang K, et al. P63. 15 Clinical Analysis of 89 Female Patients With Small Cell Lung Cancer. *Journal of Thoracic Oncology* 2021; **16**(10): S1188-S9.
169. Truong PT, Dar AR, Yu EW, Stitt LW, Videtic GMM. Shifting from hypofractionated to "conventionally" fractionated thoracic radiotherapy: A single institution's 10-year experience in the management of limited-stage small-cell lung cancer using concurrent chemoradiation. *International Journal of Radiation Oncology Biology Physics* 2003; **57**(3): 709-16.
170. Turaka A, Li T, Naik MJ, et al. Effectiveness and timing of prophylactic cranial irradiation (PCI) in limited stage small cell lung carcinoma (LS-SCLC) patients: A single institution experience. *International Journal of Radiation Oncology Biology Physics* 2013; **87**(2 SUPPL. 1): S518.
171. Twijnstra A, Boon PJ, Lormans AC, ten Velde GP. Neurotoxicity of prophylactic cranial irradiation in patients with small cell carcinoma of the lung. *European journal of cancer & clinical oncology* 1987; **23**(7): 983-6.
172. Twijnstra A, Ganzevles PGJ, De Geus BWJ, Van Vonderen RGMW, Wilmink JT, Van Oosterhout AGM. Sequelae in long-term survivors of small cell lung cancer. *International Journal of Radiation Oncology Biology Physics* 1996; **34**(5): 1037-44.
173. Twijnstra A, Ten Velde GPM, Wilmink JT, Volovics A, Van De Pol M. Efficacy and safety of prophylactic cranial irradiation in patients with small cell lung cancer. *Journal of neuro-oncology* 1997; **35**(2): 153-60.
174. Ueoka H, Ohnoshi T, Numata T, et al. [Patterns of relapse in patients with small cell lung cancer--the effect of chest irradiation and prophylactic cranial irradiation combined with intensive chemotherapy on long-term survival]. *Nihon Kyobu Shikkan Gakkai zasshi* 1990; **28**(12): 1581-7.

175. Ueki K, Matsuo Y, Kishi N, et al. Usefulness of pro-gastrin-releasing peptide as a predictor of the incidence of brain metastasis and effect of prophylactic cranial irradiation in patients with limited-stage small-cell lung cancer. *J Radiat Res* 2022; **63**(4): 636-45.
176. Ulsperger E, Karrer K, Denck H. Multimodality treatment for small cell bronchial carcinoma. Preliminary results of a prospective, multicenter trial. The ISC-Lung Cancer Study Group. *European journal of cardio-thoracic surgery : official journal of the European Association for Cardio-thoracic Surgery* 1991; **5**(6): 306-10.
177. Uprety D, Arjyal L, Vallatharasu Y, et al. Utilization of Surgery and Its Impact on Survival in Patients With Early Stage Small-cell Lung Cancer in the United States. *Clinical lung cancer* 2019.
178. Van Der Linden YM, Van Kempen ML, Struikmans H, et al. Prophylactic cranial irradiation in limited disease small-cell lung cancer in complete remission: A retrospective analysis. *Respiratory medicine* 2001; **95**(3): 235-6.
179. PS V, MH G, Krishna KJ, CD S, AL L. Outcomes and Prognostic Factors of Extensive Stage Small Cell Lung Cancer: A Retrospective Study. *South Asian Journal of Cancer* 2023.
180. Wang F, Rong J, Li H, et al. The CRP combined with LDH was an independent prognostic factor for limited stage SCLC. *Journal of Thoracic Oncology* 2015; **10**(9 SUPPL. 2): S786-S7.
181. Wang D, Xu L, Zhao L, et al. [Concurrent Chemoradiotherapy with Original Chemotherapy Regimens may not be Suitable for Patients Who Failed to Respond to Induction Chemotherapyin Limited-stage Small Cell Lung Cancer]. *Zhongguo fei ai za zhi = Chinese journal of lung cancer* 2016; **19**(12): 871-8.
182. Wang L, Cao L, Jiang R, Huang D. Prognostic Significance of Combined Biomarkers in Small Cell Lung Cancer. *Clinical laboratory* 2020; **66**(9).
183. Wei X, Allen PK, Liao Z, et al. Nadir white blood cell, absolute neutrophil, and absolute lymphocyte counts during concurrent chemoradiation therapy are associated with worse outcomes in patients with limited-stage small cell lung cancer. *International Journal of Radiation Oncology Biology Physics* 2014; **90**(1 SUPPL. 1): S623.
184. Wei X, Allen PK, Welsh JW, Lin SH, Cox JD, Komaki RU. Immediately concurrent chemoradiation therapy is associated with improved 2-year overall survival in patients with limited small-cell lung cancer. *International Journal of Radiation Oncology* 2016; **96**(2 Supplement 1): E454-E5.
185. Wei LJ, Hou Q, Yao NN, et al. [Construction of a nomogram model for predicting 2-year survival rate of small cell lung cancer based on more comprehensive variables]. *Zhonghua Yi Xue Za Zhi* 2022; **102**(17): 1283-9.
186. Wheless WH, Hughes RT, Soike M, et al. Limited-Stage Small Cell Lung Cancer: Is Prophylactic Cranial Irradiation Necessary? *International Journal of Radiation Oncology Biology Physics* 2019; **105**(1 Supplement): E550.
187. Wierzchowski M, Sprawka A, Kepka L. Isolated nodal failure after chemo-radiotherapy in limited disease small cell lung cancer (LD-SCLC). *Reports of Practical Oncology and Radiotherapy* 2009; **14**(2): 58-63.
188. Winther-Larsen A, Hoffmann L, Moeller DS, Khalil AA, Knap MM. Evaluation of factors associated with loco-regional failure and survival in limited disease small cell lung cancer patients treated with chemoradiotherapy. *Acta oncologica (Stockholm, Sweden)* 2015; **54**(9): 1574-81.

189. Work E, Bentzen SM, Nielsen OS, Fode K, Michalski W, Palshof T. Prophylactic cranial irradiation in limited stage small cell lung cancer: survival benefit in patients with favourable characteristics. *European journal of cancer (Oxford, England : 1990)* 1996; **32A**(5): 772-8.
190. Wu AJ, Gillis A, Foster A, et al. Patterns of failure in limited-stage small cell lung cancer: Implications of TNM stage for prophylactic cranial irradiation. *Radiotherapy and Oncology* 2017; **125**(1): 130-5.
191. Wu D, Fang J, Nie J, et al. [Effects of Local Radiation Combined with Chemotherapy in the treatment of Patients with Extensive-stage Small Cell Lung Cancer]. *Zhongguo fei ai za zhi = Chinese journal of lung cancer* 2015; **18**(5): 272-9.
192. Wu Q, Chen M, Peng F, et al. A study of the prognosis of patients with limited-stage small cell lung cancer who did or did not receive prophylactic cranial irradiation after effective chemoradiotherapy. *Frontiers in Oncology* 2023; **13**.
193. Wzietek I, Bialas M, Nowara E, Tukiendorf A, Bentzen S, Suwinski R. Does routine clinical practice reproduce the outcome of large prospective Trials? the analysis of institutional database on patients with limited-disease small-cell lung cancer. *Cancer investigation* 2014; **32**(1): 1-7.
194. Xanthopoulos E, Corradetti MN, Mitra N, et al. Prophylactic cranial irradiation in extensive-stage small cell lung cancer. *International Journal of Radiation Oncology Biology Physics* 2013; **87**(2 SUPPL. 1): S547.
195. Xiaodong H, Huo B, Wang L, Cao Q, Wang H. Clinical Efficacy Analysis of CT-Guided 125I Radioactive Seeds Implantation for Limited-Stage Small Cell Lung Cancer. *Brachytherapy* 2019; **18**(3 Supplement): S88.
196. Xingwen F, Pan L, Wang Y, Ye S, Wu K. Limited Stage Small Cell Lung Cancer Patients With Complete Response After Thoracic Chemoradiotherapy Still Benefit From Prophylactic Cranial Irradiation in the MRI Era. *International Journal of Radiation Oncology, Biology, Physics* 2021; **111**(3): e469.
197. Xu J, Jin B, Lou Y, et al. Prophylactic Cranial Irradiation for Patients with Surgically Resected Small Cell Lung Cancer. *Journal of Thoracic Oncology* 2017; **12**(2): 347-53.
198. Xu-Yi L. Therapeutic impact on prognosis of patients with disseminated small cell lung cancer. *Chinese Journal of Clinical Oncology* 1994; **21**(1): 28-33.
199. Yan M, Toh TS, Lindsay PE, et al. Limited-stage small cell lung cancer: Outcomes associated with prophylactic cranial irradiation over a 20-year period at the Princess Margaret Cancer Centre. *Clin Transl Radiat Oncol* 2021; **30**: 43-9.
200. Yang DX, Park HSM, Jairam V, et al. Association Between Prophylactic Cranial Irradiation and Improved Overall Survival in Small Cell Lung Cancer. *International Journal of Radiation Oncology Biology Physics* 2020; **108**(3 Supplement): e106.
201. Yao Y, Yao N, Qin Z, et al. Extensive-stage small cell lung cancer: Is prophylactic cranial irradiation necessary in the era of immunotherapy with MRI surveillance? *Precision Radiation Oncology* 2023; **7**(2): 111-7.
202. Yavaş G, Korkmaz Kirakli E, Dağdelen M, et al. Role of Consolidative Thoracic Radiotherapy for Extensive-stage Small Cell Lung Cancer: Trod Thoracic Oncology Study Group 08-006 Multi-institutional Study. *Turkish Journal of Oncology/Türk Onkoloji Dergisi* 2022; **37**(1).
203. Yildirim HC, Arzu Ergen S, Tiken EE, et al. Limited stage small cell lung cancer: Treatment results and prognostic factors. *Turk Onkoloji Dergisi* 2015; **30**(4): 188-94.

204. Yilmaz A, Tekin SB, Bilici M, Yilmaz H. The Significance of Controlling Nutritional Status (CONUT) Score as a Novel Prognostic Parameter in Small Cell Lung Cancer. *Lung* 2020; **198**(4): 695-704.

205. Yin K, Song D, Zhang H, Cai F, Chen J, Dang J. Efficacy of surgery and prophylactic cranial irradiation in stage II and III small cell lung cancer. *Journal of Cancer* 2018; **9**(19): 3500-6.

206. Yokouchi H, Ishida T, Yamazaki S, et al. Prognostic impact of clinical variables on surgically resected small-cell lung cancer: Results of a retrospective multicenter analysis (FIGHT002A and HOT1301A). *Lung cancer (Amsterdam, Netherlands)* 2015; **90**(3): 548-53.

207. Yu Y, Cao S, Fu P, et al. Radiation Induced Lymphopenia and Overall Survival in Patients with Limited Stage Small Cell Lung Cancer Receiving Definitive Chemoradiation. *International Journal of Radiation Oncology Biology Physics* 2019; **105**(1 Supplement): E550.

208. Yu J, Ouyang W, Yang Y, et al. Prophylactic cranial irradiation for extensive-stage small cell lung cancer: Analysis based on active brain MRI surveillance. *Clinical and translational radiation oncology* 2020; **25**: 16-21.

209. Yu H, Zhang J, Zhang Z, et al. One Cycle of Concurrent Chemotherapy vs. Two Cycles of Concurrent Chemotherapy With Radiation Therapy in Patients With Limited-Stage Small Cell Lung Cancer. *Front Oncol* 2021; **11**: 785022.

210. Yu L, Xu J, Qiao R, Han B, Zhong H, Zhong R. 148P Pathological stage N1 limited-stage small-cell lung cancer patients can benefit from surgical resection. *Annals of Oncology* 2022; **33**: S101.

211. Yuqiong L, Qiang H, Fan X, Chu T. 56P Long-time second-hand smoking as an independent prognostic factor in female patients with small cell lung cancer: A Chinese cohort study using propensity score matching. *Journal of Thoracic Oncology* 2021; **16**(4): S726.

212. Zahra A, Chang T, Hejleh TA, et al. Once Daily High-dose Radiation (>=60 Gy) Treatment in Limited Stage Small Cell Lung Cancer. *Journal of oncology translational research* 2016; **2**(1).

213. Zemanova M, Petruzelka L, Honova H. Prognostic factors in set of limited disease small-cell lung cancer patients treated with chemoradiotherapy. *Journal of Thoracic Oncology* 2011; **6**(6 SUPPL. 2): S1346-S7.

214. Zhang J, Fan M, Liu D, et al. Hypo- or conventionally fractionated radiotherapy combined with chemotherapy in patients with limited stage small cell lung cancer. *Radiation oncology (London, England)* 2017; **12**(1): 51.

215. Zhang J, Li H, Chen Q, et al. P2.12-22 Risk Factors for BM Incidence in SCLC: A Predictive Model for SCLC Patients on Brain Metastasis. *Journal of Thoracic Oncology* 2019; **14**(10 Supplement): S822.

216. Zhang JQ, Wang YY, Xu KP, et al. [Prognostic evaluation of nutritional indicators in patients with limited-stage small cell lung cancer]. *Zhonghua zhong liu za zhi [Chinese journal of oncology]* 2019; **41**(12): 937-42.

217. Zhang C, Liu X, Zhou Z, et al. Prophylactic Cranial Irradiation in Patients with Limited-Stage Small-Cell Lung Cancer without Brain Metastases: A Retrospective Cohort Study. *International Journal of Radiation Oncology*Biology*Physics* 2022; **114**(3, Supplement): e413-e4.

218. Zhou M, Wang Z, Yao Y, Zhou H, Liu M, Sun J. Neuron-specific enolase and response to initial therapy are important prognostic factors in patients with small cell lung cancer. *Clinical &*

translational oncology : official publication of the Federation of Spanish Oncology Societies and of the National Cancer Institute of Mexico 2017; **19**(7): 865-73.
219. Zhou N, Bott M, Park BJ, et al. Predictors of survival following surgical resection of limited-stage small cell lung cancer. *The Journal of thoracic and cardiovascular surgery* 2021; **161**(3): 760-71.e2.
220. Zhu H, Guo H, Shi F, et al. Prophylactic cranial irradiation improved the overall survival of patients with surgically resected small cell lung cancer, but not for stage I disease. *Lung cancer (Amsterdam, Netherlands)* 2014; **86**(3): 334-8.
221. Jiang L, Zhao Y, Qian J, Zhuang Y, Gao X. Retrospectively analysis of factors influencing the relapse (or progression) of newly diagnosed nonoperative small cell lung cancer. *Chinese Journal of Lung Cancer* 2015; **18**(9): 529-36.
222. S. ZDAZSDLHLMDSGMSMAR. Prophylactic cranial irradiation in extensive disease small cell lung cancer. *Oncology Research and Treatment* 2022; **45**(Suppl. 3): 6-285.
223. Zongmei Z, Zhang W, Xin D, et al. Clinical outcomes of extensive-stage small cell lung cancer patients treated with thoracic intensive modified radiation therapy (IMRT). *International Journal of Radiation Oncology Biology Physics* 2015; **93**(3 SUPPL. 1): E401.
224. Jeong J-U, Ahn S-J, Yoon MS, et al. Treatment time to the end of thoracic radiotherapy has more predictive power for survival than radiation dose intensity in patients with limited-stage small-cell lung cancer receiving concurrent chemoradiation of more than 45 Gy. *Oncology letters* 2020; **19**(1): 239-46.
225. Eze C, Roengvoraphoj O, Niyazi M, et al. Treatment Response and Prophylactic Cranial Irradiation Are Prognostic Factors in a Real-life Limited-disease Small-cell Lung Cancer Patient Cohort Comprehensively Staged With Cranial Magnetic Resonance Imaging. *Clinical lung cancer* 2017; **18**(4): e243-e9.
226. Takashi S, Takashi Seto TTTYHHHNHSMNKNKTOIKTHYMTHSKGN, Department of Thoracic Oncology NKCCFJ, et al. Prophylactic Cranial Irradiation Has a Detrimental Effect on the Overall Survival of Patients with Extensive Disease Small Cell Lung Cancer: Results of a Japanese Randomized Phase III Trial %U https://meetinglibrary.asco.org/record/93695/abstract %W ASCO Meeting Library. 2014.
227. Videtic GM, Fung K, Tomiak AT, et al. Using treatment interruptions to palliate the toxicity from concurrent chemoradiation for limited small cell lung cancer decreases survival and disease control. *Lung cancer (Amsterdam, Netherlands)* 2001; **33**(2-3): 249-58.
