# Supplementary material for: Re-examining prophylactic cranial irradiation in small cell lung cancer: a systematic review and meta-analysis
Source: eClinicalMedicine. 2024 Jan 3;67:102396. doi: 10.1016/j.eclinm.2023.102396 (PMC10796984; doi:10.1016/j.eclinm.2023.102396)
Supplement: Study Protocol [file mmc3.pdf]

Treatment outcomes associated with prophylactic intracranial radiation for small cell lung cancer: protocol for a systematic review and meta-analysis

Authors: Karolina Gaebe,[1,2] Anders W. Erickson,[3] Andrew N. Youssef,[4] Sunit Das[1,5*]

*Corresponding author: Sunit Das, sunit.das@utoronto.ca

[1] Institute of Medical Science, Faculty of Medicine, University of Toronto, 1 King's College Circle, Toronto, Ontario, M5S 3K1, Canada.
[2] Division of Endocrinology, The Hospital for Sick Children, 555 University Ave, Toronto, Ontario, M5G 1X8, Canada.
[3] Department of Laboratory Medicine and Pathobiology, Faculty of Medicine, University of Toronto, 1 King's College Circle, Toronto, Ontario, M5S 1A8, Canada.
[4] University of Western Ontario, 1151 Richmond St, London, Ontario, N6A 3K7, Canada.
[5] Division of Neurosurgery, St. Michael's Hospital, University of Toronto, 30 Bond Street, Toronto, Ontario, M5B 1W8, Canada.

Contributions: KG is the guarantor. KG and AE drafted the protocol. KG developed the search strategy and criteria for study selection and data extraction. SD provided expertise on intracranial metastatic disease from small cell lunger cancer. All authors read, provided feedback, and approved the final protocol.

Funding: KG is supported by the Graduate Diploma in Health Research (GDipHR) program at the University of Toronto. AE is supported by the MD/PhD studentship at the University of Toronto. SD is supported by an Early Researcher Award from the Province of Ontario. Access to scientific databases and Covidence software was provided through the institutional licenses of the University of Toronto. This study has no sponsor.

Role of Funding Source: N/A. Study design, data collection, management, analysis, and interpretation, as well as manuscript writing, editing, and publication were performed and decided upon solely by the original investigators.

Registration: This protocol was drafted using the Preferred Reporting Items for Systematic Review and Meta-analysis Protocols (PRISMA-P) guidelines and submitted to the International Prospective Register of Systematic Reviews (PROSPERO) April 16, 2021 (ID: 249466) and Open Science Framework (OSF) April 16, 2021 (DOI: 10.17605/OSF.IO/BC359).

Amendment 1 (April 29, 2021): A third reviewer, Bhagyashree Sharma[1], was added to the review team. The reviewer will participate in all steps of the review process
Amendment 2 (June 30, 2021): The Covidence Data Extraction 2.0 tool will be used instead of an online data extraction form. Data collection will be carried out in duplicate.

Table of Contents

Introduction *3*

Rationale **3**

Objective **4**

Methods *4*

Eligibility criteria **4**

Study design 4

PICO 5

Timing 6

Language/setting 6

Information sources **6**

Search strategy **7**

Study records **7**

Data management 7

Selection process 7

Data collection process 7

Data items **8**

Outcomes and prioritization **8**

Primary outcome 8

Secondary outcomes 8

Risk of bias individual studies **9**

Data synthesis **9**

Criteria for synthesis 9

Data measures 9

Missing data 10

Assessment of heterogeneity 10

Subgroup and sensitivity analyses 10

Meta-biases **11**

Confidence in cumulative assessments **11**

Acknowledgements **11**

References *12*

Appendix *13*

Appendix 1 – Draft MEDLINE search (OVID interface) **13**

Appendix 2 – Draft screening criteria for inclusion/exclusion of studies **14**

Appendix 3 - Draft data extraction **15**

Appendix 4 – Outcome definitions **17**

Appendix 5 – GRADE summary of findings template **18**

Introduction

Rationale

Small cell lung cancer (SCLC) is a sub-type of lung cancer that is characterized by its rapid growth rate and high incidence of metastasis. [1] Patients with SCLC are at a particularly high risk for intracranial metastatic disease (IMD); 10-15% of patients present with IMD at the time of diagnosis and the cumulative risk of developing brain metastases at 2 years is around 50 %. [2, 3] Many patients with SCLC will develop brain metastases as the sole site of relapse. [4] Despite advances in systemic treatment, prognosis for patients with SCLC remains poor particularly in patients with extensive stage (ES) disease compared to limited stage (LS) disease. [1]

Prophylactic cranial irradiation (PCI) has been recommended in this patient group after evidence showing improved overall survival (OS) and reduced incidence of IMD in patients with LS and ES disease compared to observation alone. [5-7] However, much of the current evidence was collected in an era where routine imaging was not part of clinical practice. In the hallmark trial by the European Organisation for Research and Treatment of Cancer (EORTC), for example, which established the efficacy of PCI in ES SCLC compared with observation, brain imaging was not a standard component of patient staging. [7] Therefore, the study may have included a substantial number of patients who had asymptomatic IMD. In contrast, several recent trials and meta-analyses have demonstrated no overall survival benefit with the use of PCI in patients with ES disease with mandated brain imaging, calling the practice of PCI into question in the modern era. [8, 9] Similarly, much of the evidence surrounding the efficacy of PCI in LS SCLC was established almost over two decades ago. [10, 11] More recent evidence suggests that in patients with LS disease who receive brain MRI staging, PCI may not be associated with a decreased risk of developing IMD and may not improve OS. [12] Additionally, while PCI may be effective in reducing incidence of IMD, it is also associated with a significant decline in neurocognitive function that may become more relevant to clinical decision-making as systemic treatments advance and survival for patients with SCLC improves. [13] Several meta-analyses have attempted to re-evaluate the use of PCI in SCLC, but due to restrictive eligibility criteria, these studies only included a limited number of trials.

Given the dual considerations of potential benefit and neurotoxicity associated with PCI, further investigation into PCI is warranted. Most studies limit their scope to patients with either ES or LS SCLC, and comparative assessment is lacking. Overall, little is known about the impact of PCI on safety and efficacy outcomes among the larger population of patients with SCLC. This study aims to address this uncertainty through systemic review and meta-analysis of the available literature.

Objective

The aim of this systematic review and meta-analysis is to assess the efficacy and neurotoxicity of PCI compared to observation in the management of IMD in patients with LS and ES SCLC by addressing the following questions:

1) What is the impact of PCI on OS and incidence of IMD in patients with SCLC, compared with other available treatment options?
2) What are the adverse events associated with administration of PCI in patients with SCLC compared with other available treatment options?
3) What clinical or study characteristics may explain the heterogeneity observed in the results from questions 1 and 2?

Methods

Eligibility criteria

Study design

The following studies will be included:

- Randomized controlled trials (RCTs)
- Non-randomized controlled trials (nRCTs)
- Retrospective cohort studies
- Prospective cohort studies

The following studies will be excluded:

- Case-control studies
- Cross-sectional studies
- Case reports/series
- Single-arm retrospective cohort studies[1]
- Systematic reviews/meta-analyses
- Society or treatment guidelines
- Reviews, commentaries, opinion pieces

[1] Studies labelled as "single-arm retrospective cohort studies" will be excluded from this review as these lack comparators.

PICO

Participants

We will include studies examining adult patients (age 18 or older) with histologically or cytologically confirmed SCLC in any response and any stage. Patients must have had no prior clinical diagnosis of IMD. Prior radiation to the brain, if any, must have been administered > 15 years before SCLC diagnosis. Studies reporting on other malignancies will be included provided data for SCLC is reported separately. In these cases, only data from patients with SCLC will be included.

Intervention

Inclusion:
Of interest for this analysis is PCI for the prolongation of OS and prevention of IMD. We will include studies that report on PCI as monotherapy compared to observation alone, as well as studies that compare PCI in combination with other treatment modalities. Furthermore, comparative analyses of PCI with PCI and hippocampal avoidance (HA) will be included and their results will be reported separately. If results are published on the same population, the most complete and up-to-date publication will be selected.
Exclusion:
Exploratory studies that assess the changes in patient status or tumor markers (e.g. brain volume, or circulating levels of tumor cells) before and after administration of PCI will not be included. Furthermore, studies where both treatment arms receive identical PCI regimen per study protocol will be excluded.

Comparison

Of interest are no prophylactic interventions for the treatment of IMD. Studies investigating treatments for systemic disease that include analyses for the addition of PCI will be included. In these cases, data from the relevant sub-analyses will be included while data from the main analysis will be excluded. A separate analysis comparing PCI to PCI with HA will also be performed.

Outcomes

Of interest are endpoints related to the efficacy and safety of PCI in the prevention of IMD in patients with SCLC. If reported on, data on these will be extracted and included in the meta-analysis:

- Overall survival (OS)
- Time-from-enrolment OS
- Post-IMD OS
- Incidence of IMD
- Intracranial progression-free survival (iPFS)
- Disease-free survival (DFS)
- Progression-free survival (PFS)
- Incidence of IMD as first site of recurrence

- Radiotherapy-free survival (RFS)
- Neurocognitive decline score
- Adverse events grade 1-5 (per CTCAE 5.0)

The primary outcome is OS, as this is most relevant for clarifying the role of PCI in the management of IMD in reference to comparator treatments. Time-from enrolment OS and post-IMD OS will also be collected and reported on. The secondary outcomes will be incidence of IMD, intracranial progression-free survival (iPFS), and adverse events (AE). Full definition of outcomes can be found in the appendix.

Timing

Length of study follow-up time or year of publication will not influence inclusion or exclusion of any study.

Language/setting

We will consider articles published in English as journal articles or conference abstracts. There will be no restriction by type of study setting.

Information sources

Literature search strategies will be developed using subject headings and text words related to SCLC, brain metastases, and PCI. We will search MEDLINE (OVID interface, all available years), EMBASE (OVID interface, all available years), and the Cochrane Central Register of Controlled Trials (CENTRAL, Wiley interface, current issue). The results of the literature search will be limited to the English language and human subjects. We will also scan the reference lists of included studies and relevant reviews/meta-analyses to ensure saturation and inclusion of key studies.

Grey literature sources will include ClinicalTrials.gov, Google Scholar (first 150 results), PROSPERO, and the International Clinical Trials Registry Platform of the World Health Organisation. The home pages of the following societies will be hand searched for relevant conference abstracts: Society for Neuro-oncology (SNO), American Lung Association, American Society of Clinical Oncology (ASCO), and European Society of Medical Oncology (ESMO). The search will be performed on April 17, 2021.

Search strategy

Specific search strategies for the included databases were created with the assistance of a Health Sciences Librarian with expertise in conducting scoping and systematic reviews. A draft MEDLINE search is included in Appendix 1. After the MEDLINE search is finalized, it will be adapted for use in Embase and CENTRAL. Grey literature sources and society home pages will be searched using combinations of the following keywords: “small cell lung cancer” and “brain metastases” and “prophylactic cranial irradiation”. No study design, date or language limits will be imposed on the search, although no studies in languages other than English will be included due to resources limitations.

Study records

Data management

Search results will be exported into EndNote for storage and duplicates removed using that software’s duplicate removal feature. Results will then be uploaded to Covidence, and remaining duplicates removed using that software’s native duplicate removal feature. Further remaining duplicates will then be manually removed, and results will be sorted in Covidence.

Selection process

The team will develop and test screening questions based on the eligibility criteria. Draft screening criteria are included in Appendix 2. Prior to the formal selection process, screening criteria will be tested on a set of 100 publications to refine screening questions and eligibility criteria as needed after discussion of results. The final screening tool will be uploaded to Open Science Framework, with amendments noted.

Then, two reviewers (KG and AY) will independently screen the titles and abstracts generated from the search in duplicate. Full text articles will be obtained for all publications that meet the eligibility criteria or where there is uncertainty based on review of abstracts. Subsequently, reviewers will independently screen full text articles in duplicate according to eligibility criteria. Discrepancies will be resolved through discussion. Reasons for the exclusion of full text articles will be reported. None of the selection process will be blinded. Disagreements will be resolved by discussion between reviewers, without third party arbitration, due to resource constraints. Cohen’s κ statistic for inter-rater reliability will be calculated for both title-and-abstract review and full text review.

Data collection process

A charting form will be prospectively developed by reviewers as a Google Sheets spreadsheet to determine which variables to extract, with definitions for each variable. A draft data extraction table is available in Appendix 3. We will conduct a calibration exercise on a small subset of

articles to ensure consistency between authors. Then, two reviewers (KG and AY) will extract data independently and in duplicate from each included article. A central data extraction spreadsheet will be populated by the two reviewers with extracted data after collection. Disagreements will be resolved by discussion between reviewers, without third party arbitration, due to resource constraints. Extracted data will include study details, patient demographic information, intervention details, and therapy response as well as safety outcomes.

Data items

A draft data extraction table is available in Appendix 3. Draft outcome definitions are available in Appendix 4. Extracted data will include, but are not limited to: study details (author, size, design, country, response criteria, publication type, follow-up duration, funding), patient and disease characteristics (age, sex, race, smoking status, disease extent), information on systemic therapy (type, intensity, duration), intervention and comparator details (regimen name, dose, frequency), response outcomes (OS, IMD, iPFS), and safety outcomes (adverse event rates and grades). All variables and characteristics for extraction will be determined prior to study selection and the finalized data extraction table with outcome definitions will be uploaded to Open Science Framework with amendments noted. When necessary, means and measures of dispersion will be approximated from figures in the reports.

Outcomes and prioritization

Primary outcome

Given that median survival of patients with SCLC is less than one year, the primary outcome will be median overall survival, the time between diagnosis of SCLC and death due to any cause. [14] However, all values reported for OS will be extracted independent of reporting format or time point. Overall survival estimates will be pooled separately as hazard ratios and differences in median survival months will be compared. In the event that a plurality or majority of studies report 1-year OS or OS at a time point other than one year, the more common time point or format will be used instead of overall survival, and the change noted in the final manuscript.

Secondary outcomes

1. IMD incidence, defined as incidence of IMD based on radiographic (CT or MRI) or symptomatic evidence suggestive of IMD. Values will be reported as IMD incidence rates at any time point (e.g. IMD incidence at 1-year) and hazard ratios will be collected. Values will be pooled using hazard ratio and mean difference in incidence rates.
2. Intracranial progression-free survival (iPFS), defined as median number of months until radiologic or symptomatic evidence of IMD. Values reported for median iPFS, iPFS at any time point (e.g. iPFS at 1-year), and hazard ratio will be collected. iPFS values will be

pooled using hazard ratio and mean difference in months where numbers and reporting format allow.

3. Adverse event (AE) rates, defined as the proportion of patients experiencing CTCAE (version 5.0) graded adverse events during treatment or follow-up. Where available, information on AE type, grade, and number of patients will be collected. CNS adverse events as well as changes in cognitive function, as measured by the Hopkins Verbal Learning Test – Revised (HVLT-R) are of particular interest.

OS is chosen as the primary endpoint as even patients with LS disease and good response to systemic therapy often develop IMD as their sole site of metastases, rendering IMD a major contributor to early mortality. [4] Therefore, OS is relevant in clarifying the role of PCI in patients with SCLC. IMD incidence, iPFS, and AE rates are included to provide a more complete understanding of the efficacy of PCI as IMD prophylaxis and its toxicity in this population.

Risk of bias individual studies

Risk assessment for RCTs will be undertaken using the Cochrane Collaboration Risk of Bias (RoB 2) tool (Table 8.5.a, the Cochrane Handbook for Systematic Reviews of Interventions), which covers: sequence generation, allocation concealment, blinding, incomplete outcome data (e.g. dropouts and withdrawals) and selective outcome reporting. Risk assessment for non-randomized studies will be undertaken using the Newcastle-Ottawa scale, which assesses the selection of the study groups, the comparability of the groups, and the ascertainment of either the exposure or outcome of interest, for case-control or cohort studies, respectively. Risk assessments will be scored independently by the two reviewers (KG and AY) using the tool-specific judgement criteria. Disagreement between the two reviewers will be resolved through discussion. Results of the assessments will be included as supplementary figures in the final publication.

Data synthesis

Criteria for synthesis

Meta-analyses using random-effect models will be performed on all outcomes which were reported on by at least 3 studies and where homogeneity in terms of study design and patient characteristics between studies exists. All analyses will be conducted using the statistical programming language R.

Data measures

Treatment outcomes for categorical data will be synthesized using risk ratios (RR) with 95% confidence intervals (CI). For synthesis of continuous outcomes, standardized or weighted mean differences with 95% CI will be used. Hazard ratios will be analyzed by inverse variance weighting with 95% CI.

The main analysis will pool primary and secondary outcomes of PCI versus observation. Secondary analysis will focus on primary and secondary outcomes of PCI versus PCI with HA. When a study has more than two treatment groups, additional treatment arms that are not relevant to the current analysis will not be taken into account.

Missing data

Missing data will not be imputed. When possible, hazard ratios will be calculated from Kaplan-Meier plots using R. Leave-out-one analyses will be conducted to determine the summary effect sizes of included studies for each outcome.

Assessment of heterogeneity

Clinical heterogeneity of studies will be assessed by considering the variability in participant characteristics (e.g. patient age, disease status) and study details (e.g. co-interventions, study type). I^2, τ and Q statistics will be used to test for heterogeneity across included studies. If high levels of heterogeneity ($I^2 \geq 50\%$ or $p > 0.1$) among trials exist, study design and characteristics will be analysed, and subgroup and sensitivity analyses will be performed to explain sources of heterogeneity.

Subgroup and sensitivity analyses

Subgroup analyses will be performed to explore potential sources of heterogeneity and will include the following:

- Patient characteristics (e.g. age, sex, cancer stage)
- Study design (e.g. RCT vs cohort study)
- Year of publication
- Use of baseline MRI/CT (yes vs no)
- Systemic therapy regimen and treatment response to first line therapy (e.g. CR, PR, PD)
- Type of PCI (e.g. PCI vs PCI with HA)
- SCLC disease subtypes (e.g. SCLC-A, SCLC-N)
- Post-IMD treatment (e.g. WBRT vs SRS, systemic therapy)
- Post-PCI treatment (e.g. by type of systemic therapy regimen)
- Post-PCI observation frequency

Sensitivity analyses will be performed to explore potential sources of heterogeneity, including

- Quality components (e.g. full-text vs abstracts)
- Risk of bias (e.g. omitting studies deemed to be at high risk for bias)
- Leave-out-one analyses
- Fixed- vs. random-effects models

A narrative synthesis in the manuscript text will described characteristics and findings of included studies. Results will be prioritized by outcome measures and subsequently by

subgroup comparisons. Inclusion of studies at high risk for bias will be determined after sensitivity analysis. Where quantitative synthesis is not appropriate, results will be summarized as tables and addressed in the main body of the manuscript.

Meta-biases

Visual and statistical methods for the assessment of publication biases will include visual inspection of Funnel plots and the use of Egger's regression test where more than 10 studies are available for a particular outcome. Selective reporting (outcome bias) will be evaluated using scoring from the RoB 2 tool and the Newcastle-Ottawa scale. Where possible, biases will also be assessed with reference to protocols available at ClinicalTrials.gov and the International Clinical Trials Registry Platform of the World Health Organisation. Estimates from fixed and random effects models will be compared to assess for small sample biases.

Confidence in cumulative assessments

The quality of evidence for all outcomes will be judged using the Grading of Recommendations Assessment, Development and Evaluation (GRADE) framework. A template of the summary of findings table is available in Appendix 5.

Acknowledgements

We thank Julia Martyniuk, Liason & Education Librarian at the University of Toronto, for consultation on the literature search strategies and protocol. We thank Dr. Benjamin Lok for his advice on methodology and peer review of our proposal.

References

1. van Meerbeeck, J.P., D.A. Fennell, and D.K. De Ruysscher, *Small-cell lung cancer.* Lancet, 2011. **378**(9804): p. 1741-55.
2. Hochstenbag, M.M., et al., *Asymptomatic brain metastases (BM) in small cell lung cancer (SCLC): MR-imaging is useful at initial diagnosis.* J Neurooncol, 2000. **48**(3): p. 243-8.
3. Patchell, R.A., *Brain metastases.* Neurol Clin, 1991. **9**(4): p. 817-24.
4. Arriagada, R., et al., *Prophylactic cranial irradiation for patients with small-cell lung cancer in complete remission.* J Natl Cancer Inst, 1995. **87**(3): p. 183-90.
5. Expert Panel Thoracic, M., et al., *American Radium Society Appropriate Use Criteria on Radiation Therapy for Extensive-Stage SCLC.* J Thorac Oncol, 2021. **16**(1): p. 54-65.
6. Simone, C.B., 2nd, et al., *Radiation Therapy for Small Cell Lung Cancer: An ASTRO Clinical Practice Guideline.* Pract Radiat Oncol, 2020. **10**(3): p. 158-173.
7. Slotman, B., et al., *Prophylactic cranial irradiation in extensive small-cell lung cancer.* N Engl J Med, 2007. **357**(7): p. 664-72.
8. Takahashi, T., et al., *Prophylactic cranial irradiation versus observation in patients with extensive-disease small-cell lung cancer: a multicentre, randomised, open-label, phase 3 trial.* Lancet Oncol, 2017. **18**(5): p. 663-671.
9. Yin, X., et al., *Prophylactic cranial irradiation in small cell lung cancer: a systematic review and meta-analysis.* BMC Cancer, 2019. **19**(1): p. 95.
10. Aupérin, A., et al., *Prophylactic cranial irradiation for patients with small-cell lung cancer in complete remission. Prophylactic Cranial Irradiation Overview Collaborative Group.* N Engl J Med, 1999. **341**(7): p. 476-84.
11. Meert, A.P., et al., *Prophylactic cranial irradiation in small cell lung cancer: a systematic review of the literature with meta-analysis.* BMC Cancer, 2001. **1**: p. 5.
12. Pezzi, T.A., et al., *Rates of Overall Survival and Intracranial Control in the Magnetic Resonance Imaging Era for Patients With Limited-Stage Small Cell Lung Cancer With and Without Prophylactic Cranial Irradiation.* JAMA network open, 2020. **3**(4): p. e201929-e201929.
13. Wolfson, A.H., et al., *Primary analysis of a phase II randomized trial Radiation Therapy Oncology Group (RTOG) 0212: impact of different total doses and schedules of prophylactic cranial irradiation on chronic neurotoxicity and quality of life for patients with limited-disease small-cell lung cancer.* Int J Radiat Oncol Biol Phys, 2011. **81**(1): p. 77-84.
14. Wang, S., et al., *Survival changes in patients with small cell lung cancer and disparities between different sexes, socioeconomic statuses and ages.* Sci Rep, 2017. **7**(1): p. 1339.

Appendix

Appendix 1 – Draft MEDLINE search (OVID interface)

Ovid MEDLINE: Epub Ahead of Print, In-Process & Other Non-Indexed Citations, Ovid MEDLINE® Daily and Ovid MEDLINE® <1946-Present>

#	Search Term
1	("small cell lung cancer" or "small-cell lung cancer" or "small-cell lung carcinoma" or "small cell lung carcinoma" or "SCLC").tw,kf.
2	Small Cell Lung Carcinoma/
3	Carcinoma, Small Cell/
4	Lung Neoplasms/
5	3 and 4
6	1 or 2 or 5
7	(("prophyla*" and ("crani*" or "intracrani*" or "brain" or "CNS") and ("radiation" or "irradiation" or "radiotherapy")) or "PCI").tw,kf.
8	Brain Neoplasms/
9	Central Nervous System Neoplasms/
10	exp Brain/
11	8 or 9 or 10
12	exp Neoplasm Metastasis/
13	11 and 12
14	exp Lung Diseases/ or exp Lung/
15	exp Neoplasms/
16	14 and 15
17	3 and 16
18	6 or 17
20	exp Radiotherapy/
21	13 and 20
22	7 or 21
23	18 and 22

Appendix 2 – Draft screening criteria for inclusion/exclusion of studies

Does this study examine PCI in patients with SCLC?	
**Include**	**Exclude**
• The study, or portion of the study, assesses patient outcomes following PCI in patients with LS and/or ES SCLC	• Studies without a comparator arm<br>• NSCLC studies<br>• Administration of PCI through means other than WBRT or WBRT with HA

Year, language, setting, length	
**Include**	**Exclude**
• English<br>• Any available year of publication<br>• All settings<br>• Any study length	• Non-English studies

Study Population	
**Include**	**Exclude**
• Adult human population (age ≥ 18 years)<br>• In cases where a study of patients with other types of malignancies (e.g. NSCLC) publishes a subgroup analysis of PCI in patients with SCLC, only data from the subgroup analysis will be used, and data from the main analysis will not be used.	• Non-human studies

Therapy	
**Include**	**Exclude**
• Studies reporting on PCI or PCI with HA in patients with SCLC	• Studies that do not include comparative assessments of PCI in patients with SCLC

Study type	
**Include**	**Exclude**
• Randomized controlled trials (RCTs)<br>• Non-randomized controlled trials (nRCTs)	• Case control studies<br>• Cross-sectional studies<br>• Case reports/series

• Retrospective cohort studies<br>• Prospective cohort studies	• Single-arm retrospective cohort studies<br>• Systematic reviews/meta-analyses<br>• Society guidelines or use criteria<br>• Reviews<br>• Additional types: organization reports, research thesis (e.g. PhD, Master's, undergraduate), best practices, protocols, theoretical models, interviews/observation pieces, commentaries, letters to the editor, opinion pieces

**Outcomes**	
**Include**	**Exclude**
Studies that provide any of:<br>• Overall survival (OS)<br>• Time-from enrolment OS<br>• Post-IMD OS<br>• Incidence of intracranial metastatic disease (IMD)<br>• Intracranial progression-free survival (iPFS)<br>• Radiotherapy-free survival<br>• Adverse event rates<br>• Neurocognitive function scores	Studies NOT measuring any of the provided outcomes

Appendix 3 - Draft data extraction

**Study details**	
First author (last name)	
Year	
Country	
Trial name	e.g. CLEOPATRA, NA
Study type	e.g. RCT, nRCT
Publication type	e.g. abstract, article
**Baseline patient characteristics**	
Patients total (n)	
Age, median (years)	e.g. Tx: 75, control: 55
Female (%)	
Smoker (%)	
Brain imaging prior to enrolment (y/n)	

Extend of disease	e.g. LS, ES
Largest dimension of primary tumor, median (IQR) (cm)	e.g. tx: 4.15 (2.9-5.9), control: 4.23 (2.3-6.2)
Chemotherapy regimen	e.g. standard chemotherapy, platinum-based chemotherapy
Response to primary treatment	e.g. CR, PR, NA
Duration of follow-up (months)	e.g. Tx: 75, control: 55
Performance status evaluation criteria used	e.g. ECOG, KPS
Response evaluation criteria used	e.g. RECIST 1.1, WHO
Response to systemic therapy (CR, PR, none/PD), %	e.g. tx: 50, 40, 10; control: 40, 50, 10
Neurocognitive decline tool	e.g. HVLT-R
**Intervention details**	
PCI schedule (Gy/fractions)	e.g. 25/10, 30/15
Comparator	e.g. observation, PCI with HA
**Outcomes**	
OS, median (months)	e.g. tx: 7.2, control: 3.2
OS, 1-year (%)	
OS, 2-year (%)	
OS, 3-year (%)	
OS (hazard ratio)	
Time-from-enrolment OS, median (months)	
Time-from-enrolment OS, 1-year (%)	
Time-from-enrolment OS, 2-year (%)	
Time-from-enrolment OS, 3-year (%)	
Time-from-enrolment OS (hazard ratio)	
Post-IMD OS, median (months)	
Post-IMD OS, 1-year (%)	
Post-IMD OS, 2-year (%)	
Post-IMD OS, 3-year (%)	
Post-IMD OS (hazard ratio)	
IMD incidence, 1-year (%)	
IMD incidence, 2-year (%)	
IMD incidence, 3-year (%)	
IMD incidence (hazard ratio)	
iPFS, median (months)	
iPFS, 1-year (%)	
iPFS, 2-year (%)	
iPFS, 3-year (%)	
iPFS (hazard ratio)	
DFS, median (months)	
DFS, 1-year (%)	

DFS, 2-year (%)	
DFS, 3-year (%)	
DFS (hazard ratio)	
PFS, median (months)	
PFS, 1-year (%)	
PFS, 2-year (%)	
PFS, 3-year (%)	
PFS (hazard ratio)	
Incidence of IMD as the first site of recurrence (%)	
RFS, median (months)	
RFS, 1-year (%)	
RFS, 2-year (%)	
RFS, 3-year (%)	
RFS (hazard ratio)	
Neurocognitive decline score	e.g. HVLT-R decline at 4 months
Grade 1 adverse events (n, %)	
Grade 2 adverse events (n, %)	
Grade 3 adverse events (n, %)	
Grade 4 adverse events (n, %)	
Grade 5 adverse events (n, %)	

SD: standard deviation; IQR: interquartile range; OS: overall survival; iPFS: intracranial progression-free survival; DFS: disease-free survival; PFS: progression-free survival; RFS: radiotherapy-free survival; HVLT-R: Hopkins Verbal Learning Test – Revised

Appendix 4 – Outcome definitions

OS	Length of time from diagnosis of SCLC to death due to any cause or loss to follow-up (censored)
Time-from-enrolment OS	Length of time from study enrolment to death due to any cause or loss to follow-up (censored)
Post-IMD OS	Length of time from end of PCI to death due to any cause or loss to follow-up (censored)
IMD incidence	Newly diagnosed cases of IMD based on either CT or MRI of the brain or symptoms suggestive of IMD
iPFS	Length of time from diagnosis of SCLC to radiologic or symptomatic diagnosis of IMD, death or loss of follow-up

DFS	Length of time after primary treatment for SCLC without any signs or symptoms of disease
PFS	Length of time from diagnosis of SCLC to progression or death due to any cause
RFS	Length of time from administration of PCI until salvage radiotherapy

Appendix 5 – GRADE summary of findings template

<table>
<tr><th colspan="7">Prophylactic intracranial irradiation for patients with small cell lung cancer</th></tr>
<tr><td colspan="7">Population: Patients with small cell lung cancer and no evidence of intracranial metastatic disease
Settings: primary care, community, outpatients
Intervention: prophylactic cranial irradiation
Comparison: observation</td></tr>
<tr><td rowspan="2">Outcome</td><td colspan="2">Illustrative comparative risk (95% CI)</td><td rowspan="2">Relative Effect (95% CI)</td><td rowspan="2">No of participants (studies)</td><td rowspan="2">Quality of the evidence (GRADE)</td><td rowspan="2">Comments</td></tr>
<tr><td>Assumed risk
Observation</td><td>Corresponding risk
PCI</td></tr>
<tr><td></td><td></td><td></td><td></td><td></td><td>⊕⊕○○</td><td></td></tr>
<tr><td></td><td></td><td></td><td></td><td></td><td></td><td></td></tr>
<tr><td colspan="7">Footnotes to be inserted here</td></tr>
</table>
